# Cytotoxic Compounds from Marine Fungi: Sources, Structures, and Bioactivity

**DOI:** 10.3390/md22020070

**Published:** 2024-01-28

**Authors:** Yukang Gao, Jianjian Wang, Pornphimon Meesakul, Jiamin Zhou, Jinyan Liu, Shuo Liu, Cong Wang, Shugeng Cao

**Affiliations:** 1 Key Laboratory of Chemistry and Engineering of Forest Products, State Ethnic Affairs Commission, Guangxi Key Laboratory of Chemistry and Engineering of Forest Products, Key Laboratory of Universities in Guangxi for Excavation and Development of Ancient Ethnomedicinal Recipes, Guangxi Collaborative Innovation Center for Chemistry and Engineering of Forest Products, Guangxi Minzu University, Nanning 530006, China; 18262343358@163.com (Y.G.); wangjianjian0114@163.com (J.W.); m14795747203@163.com (J.Z.); cheese77877@163.com (J.L.); 18231488462@163.com (S.L.); 2 Department of Pharmaceutical Sciences, Daniel K. Inouye College of Pharmacy, University of Hawai’i at Hilo, Hilo, HI 96720, USA; pmeesak@hawaii.edu

**Keywords:** marine fungi, chemical structures, marine natural products, antitumor activity

## Abstract

Marine fungi, such as species from the *Penicillium* and *Aspergillus* genera, are prolific producers of a diversity of natural products with cytotoxic properties. These fungi have been successfully isolated and identified from various marine sources, including sponges, coral, algae, mangroves, sediment, and seawater. The cytotoxic compounds derived from marine fungi can be categorized into five distinct classes: polyketides, peptides, terpenoids and sterols, hybrids, and other miscellaneous compounds. Notably, the pre-eminent group among these compounds comprises polyketides, accounting for 307 out of 642 identified compounds. Particularly, within this collection, 23 out of the 642 compounds exhibit remarkable cytotoxic potency, with IC_50_ values measured at the nanomolar (nM) or nanogram per milliliter (ng/mL) levels. This review elucidates the originating fungal strains, the sources of isolation, chemical structures, and the noteworthy antitumor activity of the 642 novel natural products isolated from marine fungi. The scope of this review encompasses the period from 1991 to 2023.

## 1. Introduction

The realm of marine natural products encompasses a broad array of chemical compounds obtained from various marine sources, including algae, sponges, corals, cnidarians, bryozoans, mollusks, tunicates, echinoderms, marine microorganisms, phytoplankton, and various other miscellaneous origins [1]. While there is evidence that the overall count of marine natural products is on the rise, there is a discernible trend suggesting that the degree of novelty associated with these discoveries may be waning. Nevertheless, recent years have witnessed a substantial upswing in both the absolute quantity and the pace of discovery of marine natural products [2]. Notably, among the diverse spectrum of marine natural products, those originating from microorganisms have emerged as a significant wellspring of lead compounds known for their exceptional biological activities [3]. In recent years, researchers have increasingly come to recognize the tremendous value of marine fungi as prolific sources of marine natural products, primarily owing to the secondary metabolites they produce, distinguished by their unique structural characteristics and remarkable bioactive properties [4]. An examination of the literature regarding marine microbial natural products from 2010 to 2013 reveals a noteworthy pattern: the majority of these compounds, specifically 576 out of 859, have been isolated from marine fungi [5]. This review is dedicated to exploring three fundamental aspects: firstly, it provides an in-depth analysis of the origins of marine fungal strains, elucidating the distinctive environments and ecological niches from which these fungi are cultivated. Secondly, it offers detailed insights into the structural attributes of the secondary metabolites derived from marine fungi, underscoring their novelty and complexity. Lastly, the review meticulously scrutinizes the cytotoxic properties of a notable total of 642 compounds that have been isolated from marine fungi. These findings are thoroughly compiled from reports spanning the 1991–2023 period, offering a comprehensive and up-to-date exploration of this subject within scientific literature.

## 2. Structural Classes of Antitumor Secondary Metabolites from Marine Fungi

### 2.1. Polyketides

#### 2.1.1. Macrolides, Lactones, Pyrones, and Lactams

**Figure 1:**
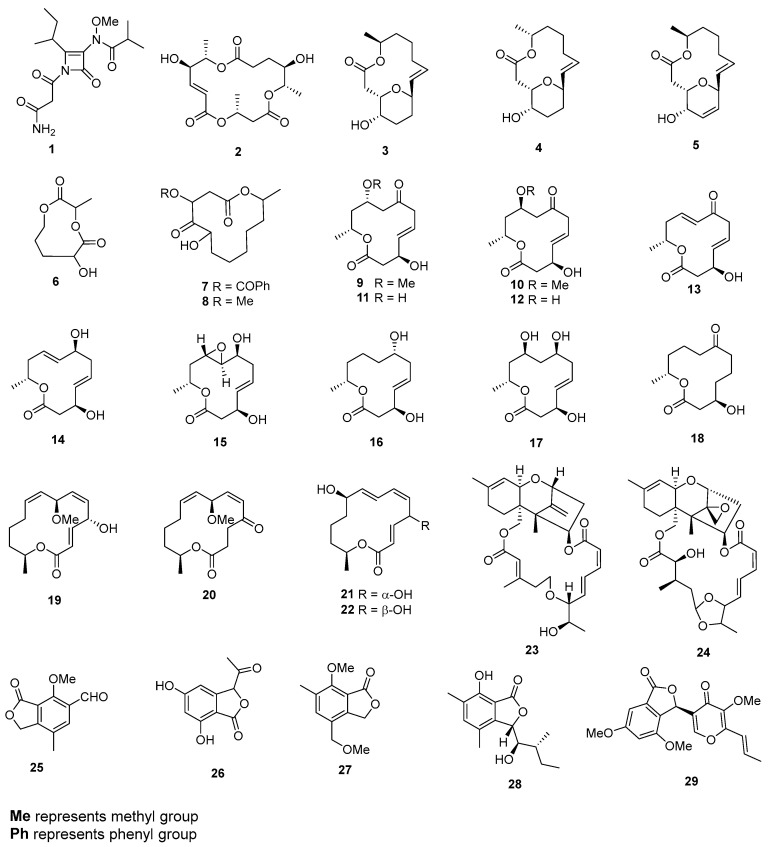
Structures of compounds **1**–**29**.

*Hyphomycetes* sp. has been found to yield a novel azetinone (α,β-unsaturated-β-lactam) named kasarin (**1**) (Figure 1), which exhibited cytotoxic effects against P388 cells with an IC_50_ value of 34 µg/mL [6]. From the fungus *Periconia byssaides* OUPS-N133, a 16-membered macrolide known as macrosphelide I (**2**) was isolated, and it displayed cytotoxicity against P388 cells with an ED_50_ value of 20.0 µg/mL [7]. Additionally, three newly discovered 14-membered macrolides, named aspergillides A–C (**3**–**5**), were isolated from a marine-derived fungus, *Aspergillus ostianus* strain 01F313. These compounds exhibited cytotoxic activity against mouse lymphocytic leukemia cells (L1210) with LD_50_ values of 2.1, 71.0, and 2.0 μg/mL, respectively [8]. A nine-membered lactone, cladospolide E (**6**), was isolated from *Cladosporium* sp. F14, and it displayed modest cytotoxicity towards HeLa, A435, A549, and K562 cells [9]. *Cladosporium* sp. L037 produced two new 12-membered macrolides, sporiolides A (**7**) and B (**8**), which demonstrated cytotoxicity against murine lymphoma L1210 cells, with IC_50_ values of 0.13 and 0.81 µg/mL, respectively [10]. *Dendrodochium* sp. produced ten new 12-membered macrolides known as dendrodolides A–E (**9**–**13**), G–I (**14**–**16**), and K–L (**17**–**18**). These compounds exhibited varying levels of growth-inhibitory activity against SMMC-7721 and HCT116 cells. Specifically, compounds **9**–**12**, and **15**–**17** displayed cytotoxicity against SMMC-7721 cells, with IC_50_ values of 19.2, 24.8, 18.0, 15.5, 21.8, 14.7, and 21.1 µg/mL, respectively. Additionally, compounds **11**, **13**, **14**, and **16**–**18** showed cytotoxicity against HCT116 cells, with IC_50_ values of 13.8, 5.7, 9.8, 11.4, 15.9, and 26.5 µg/mL, while the IC_50_ value of adriamycin as a positive drug for SMMC-7721 and HCT116 cells were 2.6 and 2.4 µg/mL, respectively [11]. From *Pestalotiopsis microspore*, 7-*O*-methylnigrosporolide (**19**) and pestalotioprolides D–F (**20**–**22**) were isolated. These compounds (**19**–**22**) displayed significant cytotoxicity against L5178Y cells, with IC_50_ values of 0.7, 5.6, 3.4, and 3.9 µM, respectively, whereas the IC_50_ value of kahalalide F as a positive drug for L5178Y cells was 4.3 µM. Additionally, compound **21** showed potent cytotoxicity against A2780 cells, with an IC_50_ value of 1.2 µM, while the IC_50_ value of cisplatin as a positive drug for A2780 cells was 1.2 µM [12]. A new macrocyclic trichothecene, 12,13-deoxyroridin E (**23**), was produced by *Myrothecium roridum* 98F42. This compound exhibited cytotoxic effects against L1210 and HL-60 cells, with IC_50_ values of 15 and 25 ng/mL, respectively [13]. A new macrocyclic trichothecene, named roridin R (**24**), was isolated from *Myrothecium* sp. TUF 02F6. This compound exhibited cytotoxic properties against L1210 cells, with an IC_50_ value of 0.45 μM [14]. Diaporthelactone (**25**), obtained from *Diaporthe* sp., demonstrated cytotoxic effects on both KB and Raji cell lines, displaying IC_50_ values of 6.25 and 5.51 µg/mL, respectively [15]. Acetophthalidin (**26**) was isolated from *Penicillium* sp. BM923, which completely inhibited the cell cycle progression of tsFT210 cells in the G2/M phase at a final concentration of 6.25 µg/mL [16]. *Penicillium* sp. ZH58 produced 4-(methoxymethyl)-7-methoxy-6-methyl-1(3H)-isobenzofuranone (**27**), which displayed cytotoxic activity against KB and KBV200 cells, yielding IC_50_ values of 6 and 10 µg/mL, respectively [17]. Chrysoarticulin C (**28**), isolated from *Chrysosporium articulatum*, showed cytotoxicity against K562 and A549, with IC_50_ values of 25.4 and 34.5 µM, whereas a positive control (doxorubicin) for K562 and A549 cells displayed IC_50_ values of 4.8 and 2.8 µM, respectively [18]. A phthalide derivative (**29**) was extracted from *Guignardia* sp. 4382. This compound exhibited cytotoxic effects on both KBv200 and KB cells, with IC_50_ values of 15.1 and 20.0 µg/mL, respectively, while the IC_50_ values of cisplatin as a positive control for these cells were 0.78 and 2.8 µM [19].

**Figure 2:**
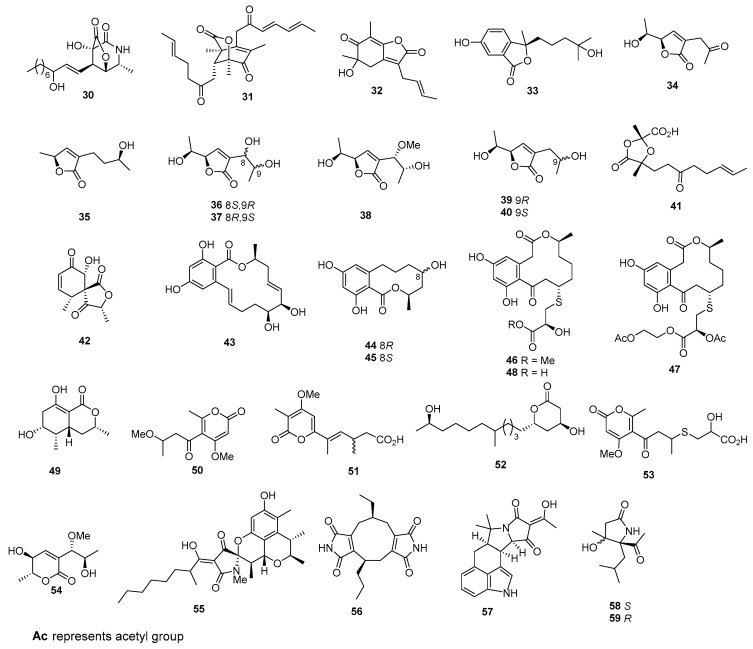
Structures of compounds **30**–**59**.

*Acremonium* sp. AWA16-1 yielded a γ-lactone-δ-lactam ring, named awajanomycin (**30**) (Figure 2), which inhibited the growth of the A549 cell, with an IC_50_ value of 27.5 µg/mL [20]. Dihydrotrichodermolide (**31**) and phialofurone (**32**) were isolated from *Phialocephala* sp. FL30r. These compounds exhibited cytotoxicity against K562 (IC_50_ values of 11.5 and 0.2 μM) and P388 (IC_50_ values of 22.9 and 22.4 μM) [21]. *Pseudallescheria boydii* yielded pseudaboydin A (**33**), which showed cytotoxic activity against SUNE1, HONE1, and GLC82, with IC_50_ values of 46.5, 37.1, and 87.2 µM [22]. Aspiketolactonol (**34**) and aspilactonols A–F (**35**–**40**) were isolated from *Aspergillus* sp. 16-02-1. These compounds exhibited significant cytotoxic activities, with inhibitory rate (IR%) values at 100 µg/mL between 10% and 79% against the human cancer cell lines K562, HL-60, HeLa, and BGC-823, while the positive control docetaxol inhibited these cell lines, with IR% values of 55.6%, 49.9%, 45.1%, and 61.5% at 100 μg/mL [23]. *Trichoderma citrinoviride* yielded citrinoviric acid (**41**), which exhibited moderate cytotoxic effects on the A-375 cell line, with an IC_50_ value of 85.7 μM [24]. *Verruculina enalia* BCC 22226 produced rosigenin analogues (**42**), which displayed cytotoxicity against MCF-7, NCI-H187, and Vero cell lines, with IC_50_ values of 17.88, 4.98, and 6.24 µg/mL [25]. *Aigiaus parvus* sp. BCC 5311 produced aigialomycin D (**43**), which exhibited cytotoxicity against Vero cells, as well as KB and BC-1, with IC_50_ values of 1.8, 3.0, and 18.0 µg/mL, while the positive control, ellipticine, inhibited these cell lines, with IC_50_ values of 1.0, 0.46, and 0.6 μg/mL, respectively [26]. The mangrove endophytic fungus Zh6-B1 yielded two new 10-membered resorcylic (**44**–**45**), which exhibited the antiproliferative activity against KV and MDR, with inhibitions from 42.4% to 41.6% at the concentration of 100 µM [27]. *Penicillium sumatrense* MA-92, a fungus obtained from the rhizosphere of the mangrove *Lumnitzera racemosa*, yielded sumalarins A−C (**46**–**48**), which showed potent cytotoxicity against MCF-7, HeLa, Huh 7, NCI-H460, SGC-7901, SW1990, and DU145, with IC_50_ values ranging from 3.8 to 11 µM, whereas the positive control inhibited these cell lines, with IC_50_ values ranging from 0.011 to 12 μg/mL [28]. Ramulosin derivative (**49**) was isolated from MF593, which showed 65% growth inhibition against HeLa cells at a concentration of 50 µg/mL [29]. Pyrenocine E (**50**) was isolated from *Penicillium waksmanii* Zaleski OUPS-N133. This compound exhibited cytotoxic activity against P388, with an ED_50_ value of 1.30 µg/mL [30]. *Petriella* sp. TUBS 7961 yielded α-pyrone derivative **51**, which showed active cytotoxic activity against L5178Y, with an ED_50_ of 0.2 µg/mL [31]. One polyketide derivative, named penicitide A (**52**), was isolated from *Penicillium chrysogenum* QEN-24S, which exhibited moderate cytotoxic activity against the human hepatocellular liver carcinoma cell line HepG2, with an IC_50_ value of 32 µg/mL [32]. *Penicillum citreonigrum* XT20-134 (MCCC 3A00956) produced 2-hydroxyl-3-pyrenocine-thio propanoic acid (**53**), which showed potent cytotoxicity to Bel7402, HT1080, Cne2, and A549 cell lines, with IC_50_ values of 7.63 ± 1.46, 10.22 ± 1.32, 73.14 ± 5.32, and 87.08 ± 7.32 µM, while the IC_50_ values of paclitaxel as a positive control against these cell lines were less than 1 µM [33].

Aspyronol (**54**) was isolated from *Aspergillus* sp. 16-02-1. This compound exhibited significant cytotoxic activities, with inhibitory rate (IR%) values at 100 µg/mL between 10 and 79% against human cancer cell lines K562, HL-60, HeLa, and BGC-823 [23]. Penicitrinine A (**55**) was isolated from *Penicillium citrinum*. This compound demonstrated cytotoxic effects on A-375, SPC-A1, and HGC-27 cancer cell lines, resulting in IC_50_ values of 20.1, 28.6, and 29.4 µM, respectively [34]. A new diimide derivative (**56**) was obtained from a combination of two mangrove fungi strains (nos. K38 and E33). This compound exhibited weak cytotoxic activity against Hep-2 and HepG2 cells, with IC_50_ values of 45 and 51 μg/mL [35]. Iso-*α*-cyclopiazonic acid (**57**) was isolated from *Aspergillus flavus*. Compound **57** showed cytotoxicity against A549, with an IC_50_ value of 42.2 μM [36].

**Figure 3:**
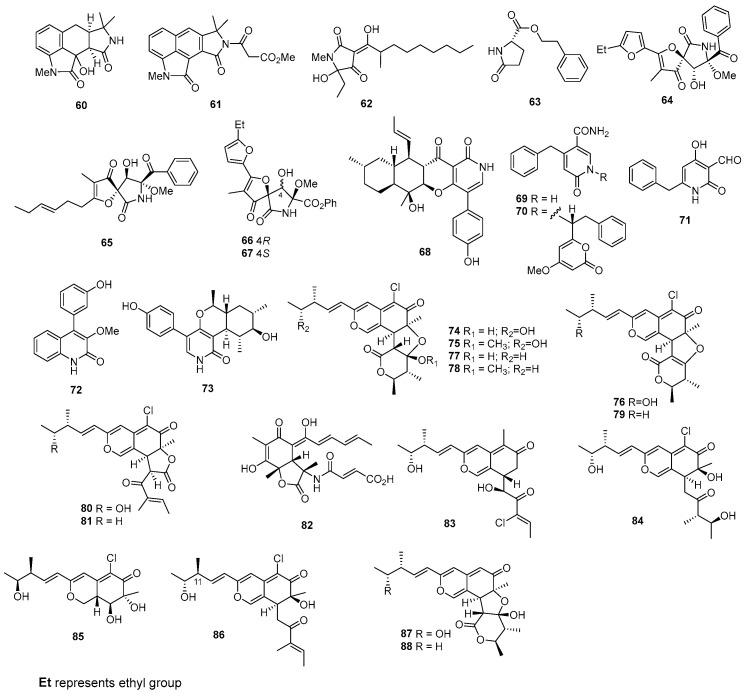
Structures of compounds **60**–**88**.

Monascuslactams C–D (**58**–**59**) were isolated from *Monascus albidus* BB3. Among these compounds, compound **58** showed cytotoxicity against SUNE1, HepG2, MDA-MB-231, and Ges-1, with IC_50_ values of 28.66 ± 1.10, 26.48 ± 0.10, 24.55 ± 3.63, and 14.54 ± 0.83 µM, while compound **59** showed cytotoxicity against SUNE1, HepG2, QGY7701, MDA-MB-231, ChangLiver, and Ges-1, with IC_50_ values of 17.28 ± 0.81, 12.55 ± 0.10, 32.90 ± 2.71, 12.67 ± 0.60, 34.83 ± 3.51, and 7.13 ± 0.52 µM. Meanwhile, the positive control, cisplatin, showed cytotoxic effects on SUNE1, HepG2, QGY7701, ChangLiver, and Ges-1, with IC_50_ values of 1.16 ± 0.23, 1.06 ± 0.03, 3.52 ± 0.11, 6.55 ± 0.51, and 1.06 ± 0.04 µM, whereas the positive control, adriamycin, exhibited cytotoxic activity against MDA-MB-231, with an IC_50_ value of 0.07 ± 0.03 µM [37]. Speradines B (**60**) and E (**61**) (Figure 3), two new tetracyclic oxindole alkaloids, were isolated from *Aspergillus oryzae*. These compounds displayed weak cytotoxicity against Hela, with IC_50_ values of 0.20 and 0.20 mM [38]. *Trichoderma citrinoviride* yielded penicillenol D (**62**), which exhibited moderate cytotoxic effects on the A-375 cell line, with an IC_50_ value of 32.6 μM [24]. Moreover, 5-oxo-l-prolinate (**63**) was isolated from *Aspergillus versicolor* ZBY-3, which showed cytotoxic activity against HeLa, with an IC_50_ value of 49.0 μg/mL [39]. *Aspergillus sydowi* D2-6 produced new heterospirocyclic γ-lactam, azaspirofuran A (**64**), which displayed cytotoxic activity against A549, with an IC_50_ value of 10 μM [40]. The strain *Aspergillus fumigatus* OUPS-T106B-5 produced cephalimysin A (**65**), along with cephalimysins C (**66**) and D (**67**). Cephalimysin A (**65**) demonstrated notable cytotoxicity against HL-60 and P388 cells, with IC_50_ values of 9.5 and 15.0 nM, respectively [41]. Cephalimysins C and D (**66** and **67**) have demonstrated cytotoxic effects against HL-60 and P388 cells, with IC_50_ values of 58.4 and 48.7 μM for cephalimysin C, and 53.5 and 51.5 μM for cephalimysin D, respectively, whereas the positive control, 5-fluorouracil, inhibited these cell lines, with IC_50_ values of 2.2 and 2.5 μM [42]. *Campylocarpon* sp. HDN13-307 yielded campyridone D (**68**), which were cytotoxic against the HeLa cell, with the IC_50_ value of 8.8 µM, while the positive control, adriamycin, showed cytotoxicity against the Hela cell, with an IC_50_ value of 0.6 μM [43]. Aspernigrins A and B (**69** and **70**) were derived from *Aspergillus niger*. These compounds effectively hindered the growth of human tumor cells at a concentration of 50 μg/mL [44]. A novel pyridone derivative, named carbonarone B (**71**), was isolated from the culture of the marine-derived fungus *Aspergillus carbonarius* WZ-4-11. This compound demonstrated cytotoxic effects against K562 cells, with an IC_50_ value of 27.8 µg/mL [45]. A novel phenylquinolinone (**72**) was isolated from *Aspergillus versicolor* Y31-2. This compound demonstrated moderate cytotoxicity against MCF-7 and SMMC-7721 cells, with IC_50_ values of 16.6 and 18.2 µM, respectively [46]. Chaunolidone A (**73**), isolated from *Chaunopycnis* sp. CMB-MF028, showed potent inhibitor of the human nonsmall-cell lung carcinoma cell NCI-H460, with the IC_50_ value of 0.09 µM [47]. 

Chaetomugilins A–C (**74**–**76**) [48,49], D–F (**77**–**79**) [49], and N–O (**80**–**81**) [50] are chloroazaphilone derivatives, were obtained from *Chaetomium globosum* OUPS-T106B-6. Compounds **74**–**79** displayed cytotoxic effects against HL-60 and P388 cells, with IC_50_ values ranging from 1.3 to 16.5 and 3.3 to 18.7 µM, whereas 5-fluorouracil, as a positive control, inhibited HL-60 and P388 cells, with IC_50_ values of 2.7 and 1.7 μM [48,49]. In addition, compounds **80** and **81** demonstrated cytotoxic effects against P388, HL-60, L1210, and KB cells. Compound **80** exhibited IC_50_ values of 2.3 µM for P388 and HL-60, and 10.6 µM for L1210 and KB, whereas compound **81** displayed IC_50_ values of 11.1 µM for P388 and HL-60, 10.1 µM for L1210. and 7.2 µM for KB, while 5-fluorouracil as a positive control inhibited P388, HL-60, L1210, and KB cells, with IC_50_ values of 1.7, 2.7, 1.1, and 7.7 μM [50]. A novel sorbicillin-derived compound named sorbicillactone A (**82**) was obtained from a strain of *Penicillium chrysogenum*. This compound exhibited potent cytotoxicity against L5178y leukemic cells, with an IC_50_ value of 2.2 μg/mL [51].

**Figure 4:**
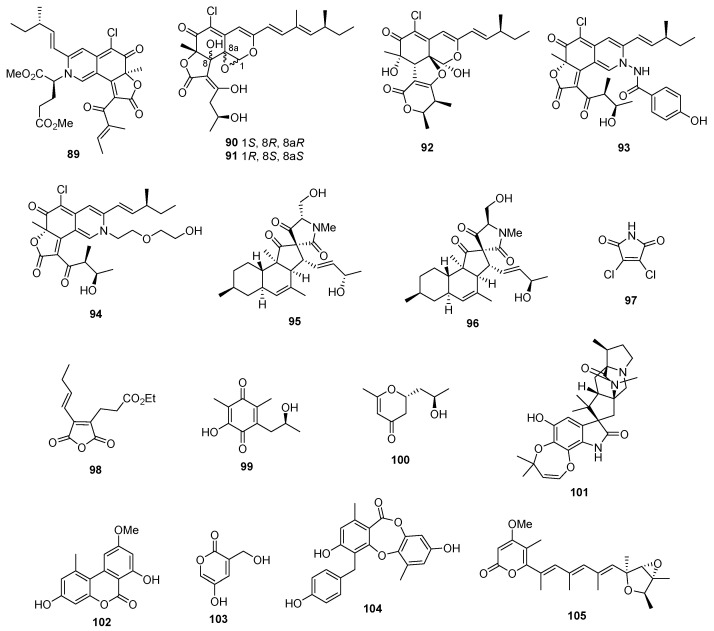
Structures of compounds **89**–**105**.

*Chaetomium globosum* OUPS-T106B-6 produced chaetomugilins P–R (**83**–**85**) and 11-epichaetomugilin I (**86**). The cytotoxicity of compounds **83**–**86** was assessed against P388, HL-60, L1210, and KB cells. Compounds **83** and **86** displayed strong cytotoxic effects, with IC_50_ values ranging from 0.7 to 1.8 pM, whereas 5-fluorouracil as a positive control inhibited P388, HL-60, L1210, and KB cells, with IC_50_ values of 1.7, 2.7, 1.1, and 7.7 μM. In contrast, compounds **84** and **85** exhibited significant cytotoxicity; their IC_50_ values fell within a range of 32.0 to greater than 100 pM [52]. Dechloro-chaetomugilins A (**87**) and D (**88**) were identified in *C. globosum* OUPS-T106B-6. These compounds exhibited moderate inhibitory effects on the growth of cultured P388, HL-60, L1210, and KB cell lines, with IC_50_ values ranging from 57.4 to greater than 100 µM [53]. A chloroazaphilone derivative called *N*-glutarylchaetoviridin C (**89**) (Figure 4) was isolated from *Chaetomium globosum* HDN151398. This compound demonstrated notable cytotoxicity against MGC-803 and HO8910 cells, with IC_50_ values of 6.6 and 9.7 µM, respectively [54]. *Phomopsis tersa* FS441 produced chloroazaphilone derivatives known as tersaphilones D (**90**) and E (**91**). These compounds exhibited remarkable cytotoxicity against SF-268, MCF-7, HEPG-2, and A549 cell lines, with IC_50_ values ranging from 5.4 to 8.3 µM, while cisplatin as a positive control inhibited these cells, with IC_50_ values of 3.3 ± 0.3, 3.2 ± 0.1, 2.4 ± 0.1, and 1.6 ± 0.1 µM [55]. *Chaetomium* sp. NA-S01-R1 was the source of chaephilone C (**92**) and chaetoviridides A and B (**93** and **94**). Remarkably, compound **93** demonstrated significant cytotoxicity against Hep G2 cells, with an IC_50_ value of 3.9 µM. Conversely, compounds **92** and **94** exhibited enhanced cytotoxic activities against HeLa cells, with IC_50_ values ranging from 5.6 to 7.7 µM, whereas doxorubicin as a positive control inhibited Hep G2 and HeLa, with IC_50_ values of 1.1 ± 0.1 and 0.5 ± 0.1 µM, respectively [56]. Pyrenosetins A and B (**95** and **96**) were discovered in *Pyrenochaetopsis* sp. FVE-001. These compounds demonstrated their ability to inhibit the growth of A-375 and HaCaT cells, with IC_50_ values of 2.8 and 4.2 µM for compound **95**, and 6.3 and 35.0 µM for compound **96**, while doxorubicin as a positive control inhibited A-375 and HaCaT cells, with IC_50_ values of 0.6 and 22.1 µM [57]. A novel chlorinated pyrrole-2,5-dione metabolite (**97**) was extracted from the fungus *Mollisia* sp. SCSIO41409, which originates from mangrove sediments. This compound exhibited significant antiproliferative effects against 22Rv1 and PC-3 cell lines, with IC_50_ values of 8.35 and 9.60 μM, while docetaxel as a positive control inhibited 22Rv1 and PC-3 cell lines, with IC_50_ values of 0.03 and 0.12 µM [58]. The fungus *Talaromyces* sp. SCSIO 41050, sourced from microbes in mangrove sediment, produced a maleic anhydride derivative known as maleicanhydridane (**98**). Notably, this compound features a unique acid anhydride functional group. Maleicanhydridane (**98**) exhibited moderate cytotoxicity, with IC_50_ values of 15.5 μM against the A549 cell line and 22.9 μM against the WPMY-1 cell line, whereas docetaxel as a positive control displayed cytotoxicity against the two cell lines, with IC_50_ values of 29.95 and 0.51 µM [59]. Benzoquinone **99** was isolated from the fungus *Talaromyces* sp. MCCC3A01752, which is derived from marine sources. This compound exhibited cytotoxic properties against the MKN1 gastric cancer cell line, with an IC_50_ value of 78.0 μM. Meanwhile, the positive control cisplatin inhibited MKN1 with an IC_50_ value of 8.8 µM [60]. A newly discovered compound, (*R*)-6-((8*R*)-hydroxypropyl)-2-methyl-5,6-dihydro-4H-pyran-4-one (**100**), was isolated from *Cladosporium halotolerans* FS702. This compound exhibited notable cytotoxic activity against MCF-7, HepG-2, SF-268, and A549 cell lines, with IC_50_ values of 0.47, 0.33, 0.16, and 0.23 µM, respectively, which were superior to the positive control, doxorubicin (1.38–1.59 μM) [61]. From *Aspergillus aculeatinus* WHF0198, a novel paraherquamide called aculeaquamide A (**101**) was identified, displaying activity against Bel-7402, with an IC_50_ value of 3.3 μM [62]. *Alternaria* sp. LV52, a marine endophytic fungus, produced two new polyketides named alternariol-9-methyl ether (**102**). These polyketides demonstrated cytotoxic effects against A549 and PC3, with EC_50_ values of 2.69 and 0.64 μM, respectively [63]. Pestalotiopyrone N (**103**) was isolated from *Pestalotiopsis* sp. HQD-6, exhibiting weak cytotoxicity against the Hela cell line, with an IC_50_ value of 50.42 ± 0.07 μM, while doxirubicin as a positive control inhibited the Hela cell line, with IC_50_ values of 8.60 ± 0.10 µM [64]. *Trichoderma* sp. 307 yielded one new depsidone named botryorhodine H (**104**), which displayed potent cytotoxicity against the MMQ and GH3 cell lines, with IC_50_ values of 3.09 and 3.64 μM [65]. *Penicillium* sp. XL-01 yielded a new verrucosidin derivative named nordeoxyverrucosidin (**105**), which exhibited promising cytotoxic activity against the MGC-803, HeLa, and MDA-MB-231 cell lines, with IC_50_ values of 0.96, 3.60, and 2.91 μM, whereas cisplatin, as the positive control, inhibited these cell lines, with IC_50_ values of 1.15, 1.19, and 1.13 µM, respectively [66].

#### 2.1.2. Chromones, Xanthones, Coumarins, Benzoquinones, Naphthoquinones, Anthraquinones, and Other Aromatic Compounds

**Figure 5:**
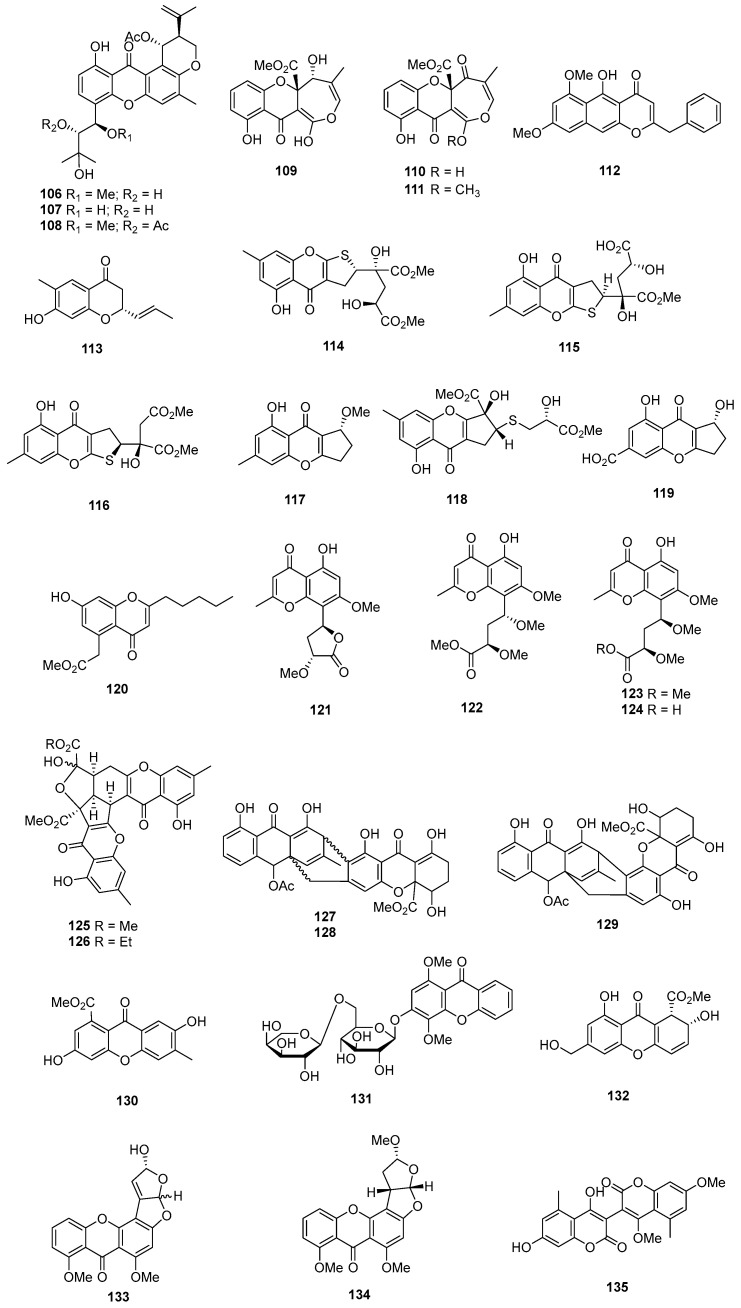
Structures of compounds **106**–**135**.

Three new prenylxanthones, named aspergixanthones A, C, and F (**106**–**108**) (Figure 5), were isolated from *Aspergillus* sp. ZA-01. Among these compounds, **106** showed selective cytotoxicity against the A-549 cell line, with the IC_50_ value of 1.8 μM, while **107** and **108** displayed broad-spectrum cytotoxicities against MDA-MB-231, MCF-7, MGC-803, HeLa, and A-549, with IC_50_ values ranging from 1.1 to 9.8 μM. Simultaneously, cisplatin as the positive control inhibited these cell lines, with IC_50_ values ranging from 0.74 to 1.3 μM [67]. Brocaenols A–C (**109**–**111**), novel cytotoxic polyketides isolated from *Penicillium brocae*, demonstrated weak cytotoxicity against the HCT-116 cell line, with IC_50_ values of 20, 50, and >50 µg/mL, respectively [68]. A newly discovered naphtho-γ-pyrone (112) from *Phomopsis* sp. ZSU-H26 exhibited cytotoxicity against Hep-2 and HepG2, with IC_50_ values of 10 and 8 μg/mL [69]. Additionally, a sorbicillinoid analogue (**113**) from *Trichoderma* sp. displayed strong cytotoxicity against MCF-7, with an IC_50_ value of 7.82 µM [70]. *Penicillium oxalicum* yielded a dihydrothiophene-condensed chromone, oxalicumone A (**114**), which showed cytotoxicity against A375 and SW-620 cell lines, with IC_50_ values of 11.7 ± 0.9 and 22.6 ± 1.5 µM, respectively, whereas cisplatin as the positive control inhibited the two cell lines, with IC_50_ values of 7.3 ± 0.8 and 30.0 ± 4.1 μM [71]. Oxalicumones D and E (**115** and **116**), isolated from *Penicillium oxalicum* SCSGAF 0023, exhibited significant cytotoxicity against various cell lines, with IC_50_ values ranging from 1.36 to 10.10 μM [72]. A mutant of *Penicillium purpurogenum* G59 through diethyl sulfate (DES) mutagenesis produced isoconiochaetone C (**117**), demonstrating significant cytotoxic activities against K562, HL-60, and HeLa cell lines [73]. Chromosulfine (**118**), a novel cyclopentachromone sulfide from the same fungus, showed toxicity against multiple cell lines, with IC_50_ values ranging from 16.7 to 75.4 µM [74]. Coniochaetone K (**119**), isolated from *Cladosporium halotolerans* GXIMD 02502, exhibited cytotoxicity against two human prostatic cancer cell lines, C4-2B and 22RV1, with inhibitions ranging from 55.8 to 82.1% at a concentration of 10 µM [75]. *Pestalotiopsis* sp. produced pestalotiopsone F (**120**), displaying cytotoxicity against the murine cancer cell line L5178Y, with an EC_50_ value of 8.93 μg/mL [76]. Highly oxygenated chromones, rhytidchromone A, B, D, and E (**121**–**124**), isolated from *Rhytidhysteron rufulum*, showed cytotoxicity against Kato-3 cell lines, with IC_50_ values ranging from 16.0 to 23.3 µM. Rhytidchromones A (**121**) and D (**123**) were active against MCF-7 cells, with IC_50_ values of 19.3 and 17.7 µM, respectively. Simultaneously, doxorubicin as the positive control inhibited MCF-7 and Kato-3, with IC_50_ values of 1.0 ± 0.1 and 2.7 ± 0.5 μM [77]. Epiremisporines B (**125**) and B1 (**126**), isolated from the diethyl sulfate (DES) mutagenesis of the marine-derived fungus *Penicillium purpurogenum* G59 exhibited cytotoxicity against K562 and HL-60 cell lines. Epiremisporine B (**125**) had IC_50_ values of 69.0 and 62.9 µg/mL, while epiremisporine B1 (**126**) had IC_50_ values of 53.1 and 54.7 µg/mL, respectively [73]. Three new xanthoquinodin compounds, JBIR-97 (**127**), JBIR-98 (**128**), and JBIR-99 (**129**), isolated from *Tritirachium* sp. SpB081112MEf2, demonstrated cytotoxic activity against ACC-MES-1, with IC_50_ values of 31, 63, and 59 µM and against Hela, and 11, 17, and 17 µM, respectively [78]. A new xanthone derivative (**130**), isolated from *Phomopsis* sp. (no. SK7RN3G1), exhibited cytotoxicity against Hep-2 and HepG2 cells, with IC_50_ values of 8 and 9 µg/mL [79]. *Phomopsis* sp. (ZH76) produced a novel xanthone derivative (**131**) that inhibited the growth of Hep-2 and HepG2 cells, with IC_50_ values of 9 and 16 µM, respectively [80].

The deep-sea-derived fungus *Engyodontium album* DFFSCS021 yielded a new chromone, engyodontiumone H (**132**), demonstrating cytotoxic activity against human histiocytic lymphoma U937, with an IC_50_ value of 4.9 µM, whereas doxorubicin as the positive control inhibited U937, with the IC_50_ value of 0.06 μM [81]. *Aspergillus nomius* NC06, isolated from the marine sponge *Neopetrosia chaliniformis*, produced two new oxisterigmatocystins, J (**133**) and K (**134**), which exhibited cytotoxic activity against HT 29 colon cancer cells, with IC_50_ values of 6.28 and 15.14 µM, while taxol, as the positive control, inhibited HT29, with the IC_50_ value of 0.48 μM [82]. *Aspergillus niger*, isolated from the Mediterranean sponge *Axinella damicornis*, yielded 3,3’-bicoumarin bicoumanigrin (**135**), inhibiting the activity of human tumor cells at concentrations ranging from 1 to 20 μg/mL [44].

**Figure 6:**
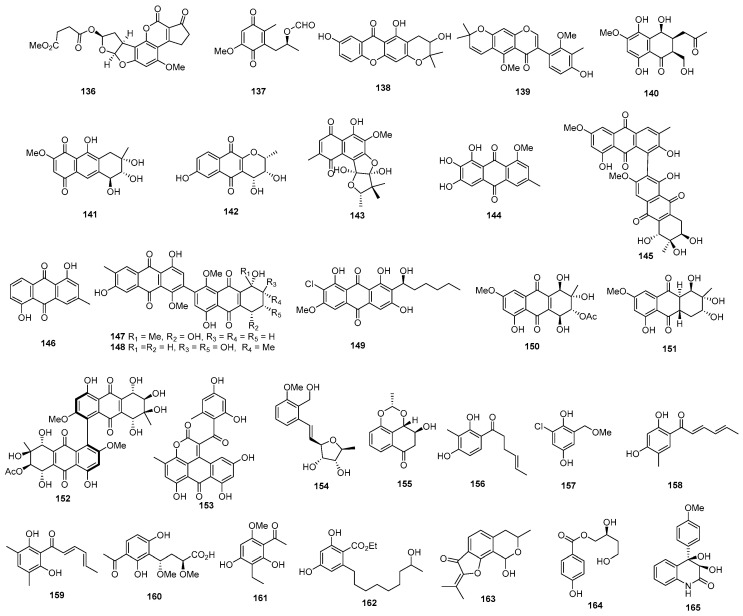
Structures of compounds **136**–**165**.

A new aflatoxin, aflatoxin B2b (**136**) (Figure 6), was isolated from *Aspergillus flavus* 092008, endogenous with the mangrove plant *Hibiscus tiliaceus* (Malvaceae). It displayed cytotoxicity against A549, K562, and L-02 cell lines, with IC_50_ values of 8.1, 2.0, and 4.2 µM, respectively [83]. An unknown pentaketide, (+)-formylanserinone B (**137**), isolated from *Penicillium* sp. obtained from deep-sea sediment, exhibited modest activity against the MDA-MB-435 cell line, with an IC_50_ value of 2.90 µg/mL [84]. A new xanthone derivative (**138**), isolated from the mangrove endophytic fungus no. ZSU-H16, displayed cytotoxicity against KB and KBV 200 cells, with IC_50_ values greater than 50 μg/mL [85]. The mangrove endophytic fungus *Fusarium* sp. ZZF41 produced a new isoflavone named 5-*O*-methyl-2′-methoxy-3′-methylalpinumisoflavone (**139**), which displayed cytotoxicity against Hep-2 and HepG2, with IC_50_ values of 4 and 11 µM [86]. Fusarnaphthoquinone A (**140**), isolated from the sea fan-derived fungi *Fusarium* spp. PSU-F135, showed cytotoxic activities against KB and MCF-7, with IC_50_ values of 130 and 22 µM, whereas doxorubicin, as the positive control, inhibited KB and MCF-7, with IC_50_ values of 0.33 and 2.18 μM [87]. Moreover, 10-deoxy-bostrycin (**141**), isolated from *Nigrospora* sp. ZJ-2010006, demonstrated cytotoxicity against A549, with an IC_50_ value of 4.56 µM, while mitomycin, as the positive control, inhibited A549, with the IC_50_ value of 3.00 μM [88]. Acaromycin A (**142**), isolated from the deep-sea-derived fungus *Acaromyces ingoldii* FS121, exhibited growth inhibition against the tumor cell lines MCF-7, NCI-H460, SF-268, and HepG-2, with IC_50_ values of less than 10 µM [89]. Herqueidiketal (**143**), possessing a novel skeleton with a highly oxidized naphthoquinone moiety, was isolated from *Penicillium* sp. It exhibited moderate cytotoxicity and significant inhibitory activity against A549, with an LC_50_ value of 17.0 µM, while the value was 3.3 μM for doxorubicin as a positive control [90]. *Emericella variecolor*, purified from the marine sponge *Haliclona valliculata*, yielded evariquinone (**144**), which displayed antiproliferative activity with inhibitory rate values of 60% and 69% against KB and NCI-H460 cells at 3.16 µg/mL [91]. Alterporriol P (**145**), isolated from *Alternaria* sp. ZJ-2008003 obtained from a *Sarcophyton* sp. soft coral in the South China Sea, exhibited cytotoxic activities against PC-3 and HCT-116, with IC_50_ values of 6.4 and 8.6 µM, whereas the value for epirubicin was 0.46 for PC-3 and it was 0.82 for HCT-116 [92]. *Halorosellinia* sp. (no. 1403) yielded compound **146**, displaying strong cytotoxicity, with IC_50_ values of 3.17 and 3.21 μM against KB and KBv200 cells, respectively [93]. *Alternaria* sp. ZJ9-6B, isolated from the mangrove *Aegiceras corniculatum* in the South China Sea, produced alterporriols K (**147**) and L (**148**), displaying moderate cytotoxic activity against MDA-MB-435 and MCF-7 cells, with IC_50_ values ranging from 13.1 to 29.1 µM [94]. *Aspergillus* sp. SCSIO F063 produced 6-*O*-methyl-7-chloroaveratin (**149**), showing inhibitory activity against SF-268, MCF-7, and NCI-H460, with IC_50_ values of 7.1, 6.6, and 7.4 µM, respectively. Meanwhile, cisplatin as a positive control showed IC_50_ values of 4.59 for SF-268, 10.23 for MCF-7, and 1.56 for NCI-H460 [95]. A new anthracene derivative, altersolanol N (**150**), isolated from *Stemphylium globuliferum*, exhibited potent cytotoxicity against L5178Y mouse lymphoma cells, with IC_50_ values in the low micromolar range [96]. Additionally, a new tetrahydroanthraquinone derivative, dihydroaltersolanol C (**151**) and acetylalterporriol E (**152**) isolated from the endophytic fungus *Stemphylium globuliferum*, showed cytotoxicity against L5178Y mouse lymphoma cells, with IC_50_ values of 3.4 and 10.4 µM [97]. Aspergiolide A (**153**), an anthraquinone derivative with a naphtho [1,2,3-de] chromene-2,7-dione skeleton, was isolated from *Aspergillus glaucus*. It demonstrated selective cytotoxicity against A-549, HL-60, BEL-7402, and P388 cell lines, with IC_50_ values of 0.13, 0.28, 7.5, and 35.0 µM, respectively [98]. 

**Figure 7:**
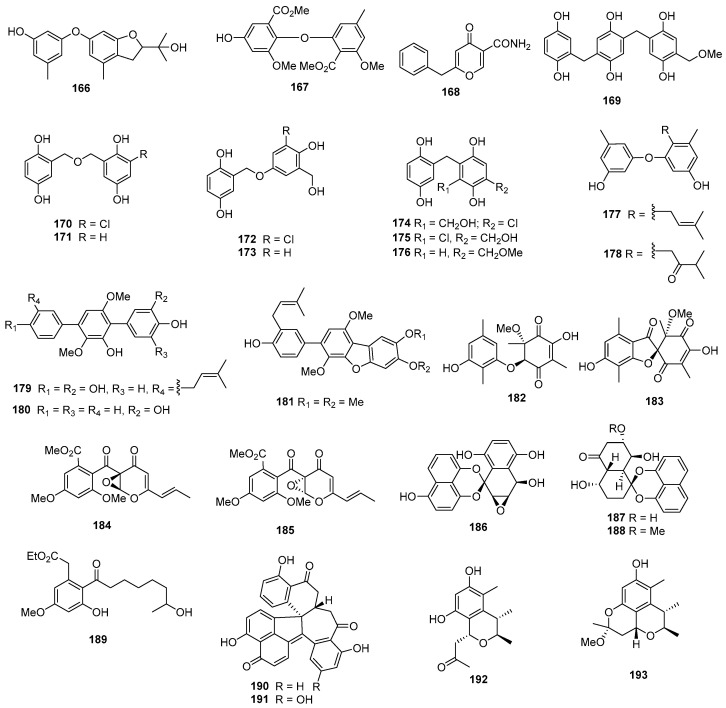
Structures of compounds **166**–**193**.

Varitriol (**154**), isolated from *Emericella variecolor*, exhibited cytotoxicity against T-47D, RXF393, and SNB-75, with GI_50_ values ranging from 1.63 × 10^−7^ to 2.44 × 10^−7^ µM [99]. Humicolone (**155**), a new phenolic tetralone in acetal form, was isolated from *Humicola grisea* Traaen, displaying cytotoxicity against KB cell lines, with IC_50_ values between 1 and 5 ppm [100]. Compound **156**, isolated from *Phialocephala* sp. FL30r, exhibited cytotoxicity against K562 and P388, with IC_50_ values of 4.8 and 0.1 μM [21]. A monomeric derivative (**157**) from the marine-derived fungus *Penicillium terrestre* showed cytotoxic effects on HL-60 with an IC_50_ value of 6.7 µM [101]. Moreover, 6-demethyl-sorbicillin (**158**), isolated from *Trichoderma* sp., demonstrated cytotoxicity against HL-60, with an IC_50_ value of 23.9 µM [102]. Isolated from *Penicillium* sp. M207142, purified from sea sediment, (2*E*,4*E*)-1-(2,6-dihydroxy-3,5-dimethyl-phenyl) hexa-2,4-dien-1-one) (**159**) showed cytotoxicity against the Hela cell line, with an IC_50_ value of 11.2 µM, and potent cytotoxicity against the SW620 cell line, with a 74% inhibition at a tested concentration of 10 µg/mL [103]. Fischerin B (**160**), isolated from the deep-sea-derived fungus *Aspergillus fischeri* FS452, showed activities against SF-268, MCF-7, HepG-2, and A549, with IC_50_ values ranging from 7 to 10 µM [104]. A novel phomalone derivative, phomalichenone F (**161**), from a deep-sea-derived fungus *Alternaria* sp. MCCC 3A00467, showed cytotoxic activity against U266 cells, with an IC_50_ value of 24.99 µg/mL [105]. *Lasiodiplodia* sp. 318#, a mangrove endophytic fungus, produced a new lasiodiplodin (**162**) that displayed cytotoxicity against MDA-MB-435, HepG2, HCT-116, A549, and THP1, with IC_50_ values of 10.1, 12.5, 11.9, 13.3, and 39.7 µM [106]. *Aspergillus pseudodeflectus* produced a new isochroman derivative named pseudodeflectusin (**163**), which displayed cytotoxicity against HeLa-S3, NUGC-3, and HL-60, with LD_50_ values of 47, 49, and 39 μM [107]. A new compound, (*S*)-2, 4-dihydroxy-1-butyl (4-hydroxy) benzoate (**164**), from the fungus *Penicillium auratiogriseum*, showed cytotoxic activity against tsFT210 cells, with a maximum inhibitory rate of 8.0 µg/mL [108]. *Penicillium janczewskii*, obtained from a marine sample, produced 3R*,4R*-dihydroxy-4-(4’-methoxyphenyl)-3,4-dihydro-2(1*H*)-quinolinone (**165**), exhibiting moderate cytotoxicity against SKOV-3 cells, with an ED_50_ value of 8.1 μM [109]. A new dihydrobenzofuran derivative, awajanoran (**166**) (Figure 7), isolated from *Acremonium* sp. AWA16-1, inhibited the growth of A549 cells, with an IC_50_ value of 17 µg/mL [110]. *Aspergillus* sp. B-F-2 produced a novel diphenyl ether dimethyl 2,3′-dimethylosoate (**167**), showing weak cytotoxicity against K562, with an IC_50_ value of 76.5 μM. Additionally, at 100 μM, the compound increased the percentage of cells in the S phase of the cell cycle from 38.3% (control) to 56.4% [111]. Carbonarones A (**168**), obtained from the culture of the marine-derived fungus *Aspergillus carbonarius* WZ-4-11, exhibited cytotoxicity against K562, with an IC_50_ value of 56.0 µg/mL [45]. Eight new gentisyl alcohol derivatives, including the trimeric terrestrol A (**169**) and dimeric terrestrols B–H (**170**–**176**), were isolated from the marine-derived fungus *Penicillium terrestre*. These new compounds demonstrated cytotoxic effects on HL-60, MOLT-4, BEL-7402, and A-549 cell lines, with IC_50_ values ranging from 5.1 to 63.2 µM [101].

Two new prenylated diphenyl ethers, diorcinols D (**177**) and E (**178**), were isolated from *Aspergillus versicolor* ZLN-60. Compound **177** displayed moderate cytotoxicity against the Hela and K562 cell lines, with IC_50_ values of 31.5 and 48.9 µM, respectively. Meanwhile, compound **178** exhibited moderate cytotoxicity only against the Hela cell line, with an IC_50_ value of 36.5 µM [112]. Prenylterphenyllin A (**179**), 4″-dehydro-3-hydroxyterphenyllin (**180**), and nylcandidusin B (**181**) were isolated from *Aspergillus taichungensis* ZHN-7-07, a root soil fungus from the mangrove plant *Acrostichum aureum*. Compound **179** displayed moderate activities against A549 and HL-60, with IC_50_ values of 8.32 and 1.53 μM. Meanwhile, compounds **180** and **181** showed moderate activities only against the P-388 cell line, with IC_50_ values of 2.70 and 1.57 μM, respectively [113]. *Aspergillus aculeatus* produced two new compounds, aculeatusquinones B (**182**) and D (**183**), showing cytotoxicity against K562, HL-60, and A549, with IC_50_ values ranging from 5.4 to 76.1 µM [114]. 

*Penicillium* sp. WC-29-5, cocultured with *Streptomyces fradiae* 007, yielded new natural products (**184**–**185**) that displayed moderate cytotoxicity against H1975 tumor cells, with IC_50_ values of 3.97 and 5.73 µM, respectively. Meanwhile, compound **185** showed moderate cytotoxicity towards the HL-60 cell, with an IC_50_ value of 3.73 µM [115]. *Ascochyta* sp. NGB4 yielded ascochytatin (**186**), a novel bioactive spirodioxynaphthalene metabolite, showing cytotoxicity against A549 and Jurkat, with IC_50_ values of 4.8 and 6.3 µM [116]. Two new spirobisnaphthalenes (**187**–**188**) were isolated from the mangrove-derived fungus *Rhytidhysteron* sp. AS21B. Among these compounds, compound **187** was active only on CaSki cells, with an IC_50_ of 22.81 µM, while compound **188** showed cytotoxic activities against CaSki and MCF-7, with IC_50_ values of 24.44 and 17.30 µM. Doxorubicin as a positive control inhibited MCF-7 and CaSki, with IC_50_ values of 0.06 and 0.20 µM [117]. The mangrove endophytic fungus *Phomopsis* sp. ZSU-H76 yielded a new polyketide (**189**) displaying cytotoxicity against HEp-2 and HepG2 cells, with IC_50_ values of 25 and 30 μg/mL, respectively [118]. Sporothrins A (**190**) and B (**191**) were isolated from the mangrove endophytic fungus *Sporothrix* sp. (#4335), displaying cytotoxicity against HepG2, with IC_50_ values of 50 and 20 μg/mL [119]. Two new citrinin derivatives, penicitrinols C (**192**) and E (**193**), were isolated from the marine-derived fungus *Penicillium citrinum*. These compounds showed weak cytotoxicity against HL-60 cells, with IC_50_ values of 52.8 and 41.2 µM [120].

**Figure 8:**
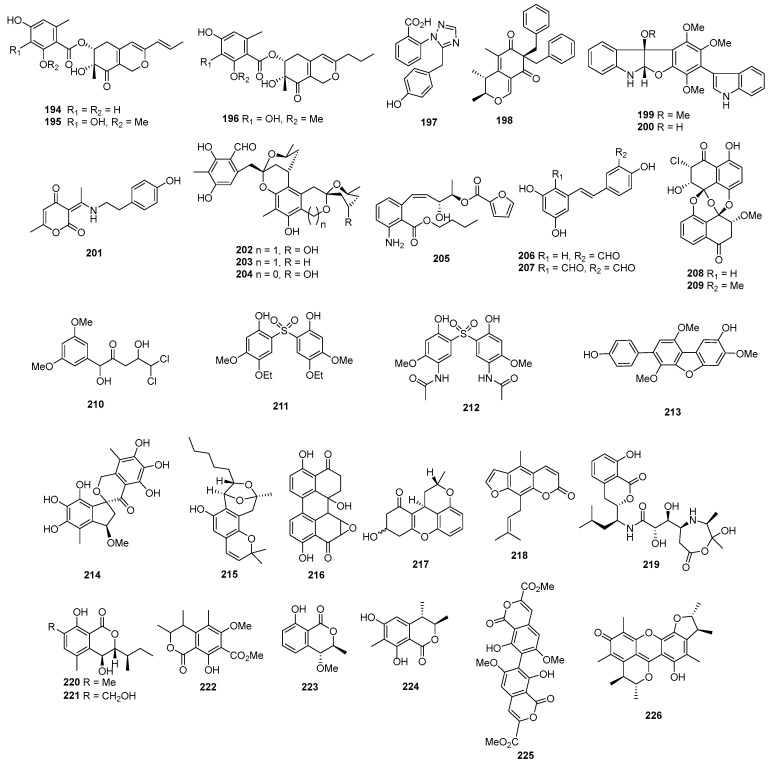
Structures of compounds **194**–**226**.

Comazaphilones D–F (**194**–**196**) (Figure 8) were isolated from *Penicillium commune* QSD-17, obtained from a marine sediment sample collected in the southern China Sea. These compounds showed cytotoxic activity against the human pancreatic tumor cell line SW1990, with IC_50_ values of 51, 26, and 53 µM, which is stronger than that of the positive control, fluorouracil (with an IC_50_ value of 120 μM) [121]. A novel triazole carboxylic acid, penipanoid A (**197**), was isolated from the marine sediment-derived fungus *Penicillium paneum* SD-44, displaying cytotoxicity against SMMC-7721, with an IC_50_ of 54.2 µM [122]. A novel benzylazaphilone derivative with an unprecedented carbon skeleton, aspergilone A (**198**), was isolated from *Aspergillus* sp. from a gorgonian *Dichotella gemmacea*. The compound showed cytotoxic activities against MCF-7, HL-60, and A549, with IC_50_ values of 25.0, 3.2, and 37.0 µg/mL [123]. *Paecilomyces variotii* EN-291, isolated from the marine alga-derived endophytic, produced varioloid A (**199**) and varioloid B (**200**), displaying cytotoxicity against A549, HCT116, and HepG2, with IC_50_ values ranging from 2.6 to 8.2 µg/mL [124]. An unusual alkaloid (**201**), isolated from *Fusarium incarnatum* (HKI0504) and purified from the mangrove plant *Aegiceras corniculatum*, exhibited weak antiproliferative effects on K-562 and HUVEC, with GI_50_ values of 37.3–37.6 µM, whereas imatinib as a positive control inhibited K-562 and HUVEC, with IC_50_ values of 0.17 and 18.5 µM. Additionally, compound **201** demonstrated cytotoxic activity against Hela, with a CC_50_ value of 23.3 µM [125]. Peniciketals A−C (**202**−**204**), three new spiroketals with a benzo-fused 2,8-dioxabicyclo [3.3.1] nonane moiety, were identified from the fungus *Penicillium raistrichii*. These compounds displayed selective cytotoxic activity against HL-60 cells, with IC_50_ values of 3.2, 6.7, and 4.5 μM, respectively, while doxorubicin as a positive control inhibited HL-60, with an IC_50_ value of 0.085 µM [126]. *Pestalotiopsis vaccinii* produced a new aromatic amine named pestalamine A (**205**), exhibiting cytotoxic activity against Hela, MCF-7, and HepG2, with IC_50_ values of 22.0, 40.3, and 32.8 µM. The IC_50_ values of the positive control taxol toward these three cell lines were 21, 5.2, and 960 nM, respectively [127]. Two new resveratrol derivatives, named resveratrodehydes A (**206**) and B (**207**), isolated from *Alternaria* sp. R6, were active against MDA-MB-435 and HCT-116, with IC_50_ values ranging from 6.9 to 8.6 µM. Epirubicin was used as a positive control for these cell lines, showing IC_50_ values of 0.56 and 0.48 µM, respectively [128]. Chloropreussomerins A (**208**) and B (**209**), two new chlorinated preussomerins, were isolated from *Lasiodiplodia theobromae* ZJ-HQ1. These compounds exhibited cytotoxicity against A549, HepG2, HeLa, MCF-7, and HEK293T, with IC_50_ values ranging from 5.9 to 27 µM, whereas epirubicin, as a positive control, inhibited these cell lines, with IC_50_ values ranging from 0.42 to 1.3 µM [129]. *Penicillum citreonigrum* XT20-134 (MCCC 3A00956) yielded 5,5-dichloro-1-(3,5-dimethoxyphenyl)-1,4-dihydroxypentan-2-one (**210**), which showed potent cytotoxicity to the human hepatoma tumor cell Bel7402, with IC_50_ values of 13.14 ± 1.41 µM, and the human fibrosarcoma tumor cell HT1080, with IC_50_ values of 16.53 ± 1.67 µM, respectively [33]. Two new sulfonyl metabolites, pensulfonoxy (**211**) and pensulfonamide (**212**), were obtained from the fermentation extract of *Penicillium aculeatum*. Pensulfonamide (**212**) showed potent preferential cytotoxicity against MCF-7 and HCT-116, with IC_50_ values of 2.18 and 6.18, while pensulfonoxy (**211**) exhibited cytotoxic activity against HCT-116, with an IC_50_ value of 5.23 μM. The IC_50_ values of the positive control, paclitaxel, exhibited in these cell lines at 0.97 and 0.52 µM, respectively [130]. *Aspergillus candidus* OUCMDZ-1051, isolated from a marine sponge (XS-3) from the Xisha islands, yielded 4-*O*-methylcandidusin A (**213**). The new compound demonstrated cytotoxic activity against 21 tumor cell lines, with IC_50_ values ranging from 0.98 to 19.1 μM among the 26 tested tumor cell lines. Notably, this compound exhibited stronger or comparable inhibitory activity to the positive control (doxorubicin) against the triple-negative breast cancer (MDA-MB-468), breast invasive ductal carcinoma (BT474), and epidermoid carcinoma (A431) cell lines, with IC_50_ values of 1.84, 6.05, and 0.98 μM, respectively [131]. The endophytic fungus *Aspergillus micronesiensis* derived from *Kappaphycus alvarezii* led to the isolation of a novel dibenzospiroketal named aspermicrone B (**214**). This compound displayed selective cytotoxic activity toward the HepG2 cell line, with an IC_50_ value of 9.9 µM [132]. A new salicylaldehyde derivative enantiomer, euroticin F (**215**), isolated from *Eurotium* sp. SCSIO F452, exhibited cytotoxicity against SF-268, MCF-7, HepG2, and A549, with IC_50_ values ranging from 21.88 to 37.31 µM, whereas the positive control (adriamycin) inhibited these cell lines, with IC_50_ ranging from 1.19 to 2.02 µM [133]. The marine endophytic *Alternaria* sp. LV52 yielded a novel polyketide named altertoxin II (**216**), which was tested and displayed cytotoxicity against A549 and PC3, with EC_50_ values of 1.15 and 0.33 μM [63]. The endophytic fungus *Penicillium* sp. GXIMD 03101 yielded a new xanthene derivative named penicixanthene E (**217**), which exhibited weak cytotoxic activity against SW1990, with an IC_50_ value of 23.8 μM [134]. *Penicillium* sp. ZH16, a mangrove endophytic fungus from the South China Sea, produced a new furanocoumarin derivative (**218**) with cytotoxicity against KB and KBV200 cell lines, having IC_50_ values of 5 and 10 µg/mL, respectively [135]. One novel isocoumarin, named Sg17-1-4 (**219**), was isolated from *Alternaria tenuis* Sg17-1, which exhibited cytotoxic activities against HeLa and A375-S2, with IC_50_ values of 0.05 and 0.3 mM [136]. Chrysoarticulins A–B (**220**–**221**), isolated from *Chrysosporium articulatum*, showed cytotoxicity against K562 and A549, with IC_50_ values of 164.0, 63.0, 147.3, and 63.2 µM, while the positive control (doxorubicin) inhibited K562 and A549, with IC_50_ values of 4.8 and 2.8 µM, respectively [18]. A new isocoumarin (**222**), isolated from the mangrove endophytic fungus (no. dz17), showed weak cytotoxic activity against Hep-2 and HepG2 cells, with IC_50_ values of 52 and 55 µg/mL [137]. A new isochroman (**223**), isolated from *Phomopsis* sp. Gx-4, showed weak cytotoxicity against Hep-2 and HepG2, with an IC_50_ value of over 50 µM [138]. A new isocoumarin, (3R*,4S*)-6,8-dihydroxy-3,4,7-trimethylisocoumarin (**224**), was isolated from *Penicillium* sp. 091402, which showed moderate cytotoxicity against the K562 cell, with an IC_50_ value of 18.9 µg/mL [139]. Two novel compounds named bipenicilisorin (**225**) and penicitrinone F (**226**) were isolated from a deep-sea-derived fungus *Penicillium chrysogenum* SCSIO 41001. Compound **225** displayed significant cytotoxic activities against K562, A549, and Huh-7 cell lines, with IC_50_ values of 6.78, 6.94, and 2.59 μM, while compound **226** showed moderate cytotoxic activity against EV71, with an IC_50_ value of 14.50 μM [140].

#### 2.1.3. Other Cyclic Polyketides

**Figure 9:**
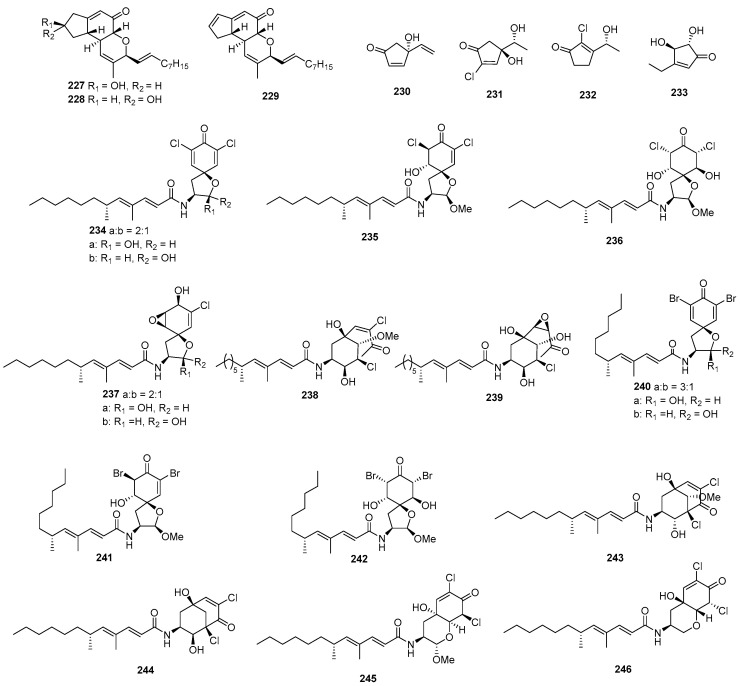
Structures of compounds **227**–**246**.

Penostatins A–C (**227**–**229**) (Figure 9) were isolated from a strain of *Penicillium* sp., originally purified from the marine alga *Enteromorpha intestinalis*. The three new compounds displayed significant cytotoxicity against P388 cells, with ED_50_ values of 0.8, 1.2, and 1.1 µg/mL [141]. *Trichoderma harzianum* OUPS-N115, separated from the sponge *Halichondria okadai*, produced trichodenones A–C (**230**–**232**), which displayed cytotoxicity against P388 cells, with ED_50_ values of 0.21, 1.21, and 1.45 µg/mL, while the positive control (mitomycin) inhibited P388, with an IC_50_ value of 0.05 µg/mL [142]. A novel cyclopentenone named trichoderone (**233**), isolated from *Trichoderma* sp. GIBH-Mf082, was active on HeLa, A549, MCF-7, and DU-145, with IC_50_ values of 85.6, 50.2, 63.5, and 43.2 μM. The IC_50_ values of the positive control cisplatin toward these cell lines ranged from 17.5 to 67 µM [143]. *Gymnascella dankaliensis* yielded 19 compounds, including gymnastatins A–C (**234**–**236**) [144], E–G (**237**–**239**) [145,146], I–K (**240**–**242**) [147], gymnastatins Q–R (**243**–**244**) [148], and dankastatins A–B (**245**–**246**) [149]. Among these compounds, compounds **234**–**236**, **238**–**239**, and **240**–**242** displayed cytotoxicity against P388, with ED_50_ values from 0.018 to 0.21 mg/mL. Meanwhile, compounds **237** and **243**–**244** showed cytotoxicity against P388, with ED_50_ values from 0.9 to 10.8 µg/mL, and the positive control (5-fluorouracil) inhibited P388, with an ED_50_ value of 0.073 µg/mL.

**Figure 10:**
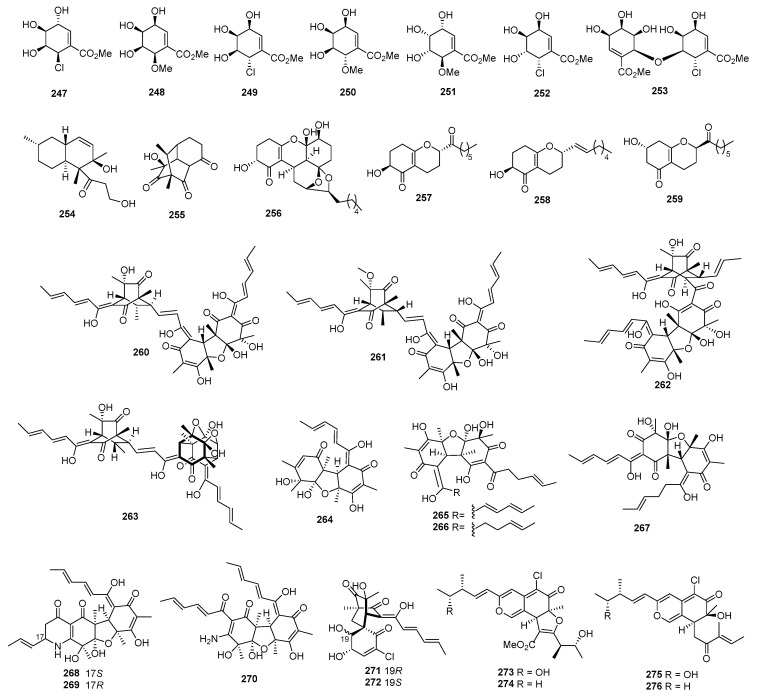
Structures of compounds **247**–**276**.

**Figure 11:**
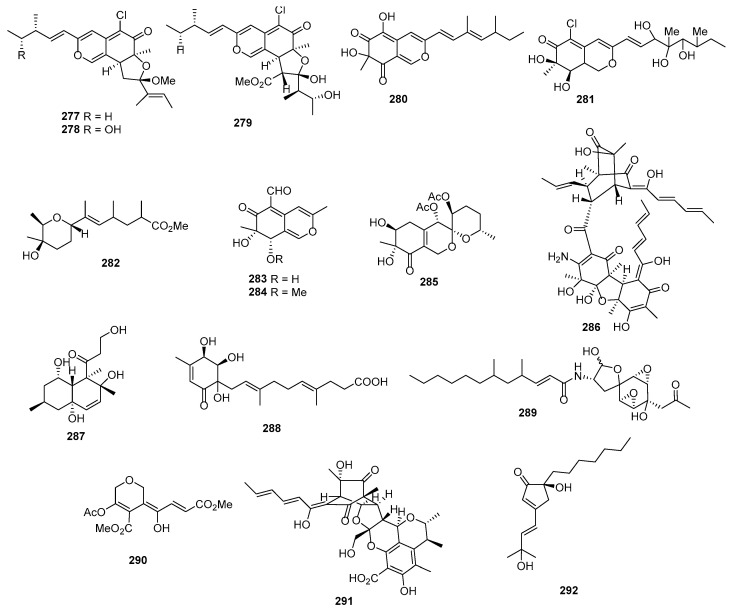
Structures of compounds **277**–**292**.

A strain of *Periconia byssoides* OUPS-N133, originally purified from the sea hare *Aplysia kurodai*, produced compounds A (**247**) and B (**248**) (Figure 10). These two compounds exhibited cytotoxicity against P388, with ED_50_ values of 0.12 and 4.0 µg/mL [150]. Moreover, the fungi yielded pericosines A–E (**249**–**253**), which were active on P388, with ED_50_ values of 0.1, 4.0, 10.5, 3.0, and 15.5 µg/mL [151]. A cultured marine fungus, *Aspergillus* sp., produced a new polyketide named aspermytin A (**254**). The compound induced neurite outgrowth in rat pheochromocytoma (PC-12) cells at a concentration of 50 μM [152]. Penicillone A (**255**) was isolated from *Penicillium terrestre*, which showed weak cytotoxicity against P388 and A-549 cell lines, with IC_50_ values of 83.0 and 68.4 µM; a positive control, VP16, inhibited P388 and A-549, with IC_50_ values of 0.064 and 1.4 µM [153]. Four new polyketide derivatives named trichodermatides A−D (**256**−**259**) were isolated from *Trichoderma reesei*. Among these compounds, trichodermatide A (**256**) has a ketal-containing pentacyclic skeleton. These compounds displayed cytotoxicity against A375-S2, with IC_50_ values of 102.2, 187.3, 38.8, and 220.0 μg/mL, respectively [154]. Compounds **260**−**263** were isolated from *Phialocephala* sp. FL30r. Among these compounds, compound **260** showed cytotoxicity against P388 and HL60 cells, with IC_50_ values of 9.10 and 3.14 μM, respectively, while the other compounds exhibited cytotoxicity against K562, with IC_50_ values of 88.2, 54.3, and 51.2 μM, and against P388 with IC_50_ values of 77.1, 78.3, and 65.7 μM [155,156]. One new compound, JBIR-59 (**264**), was isolated from *Penicillium citrinum* SpI080624G1f01. The compound exhibited cytotoxicity against the N18-RE-105 cell, with an EC_50_ value of 71 μM [157]. Two new bisorbicillinoids, compounds **265** and **266**, were isolated from *Penicillium terrestre*. These compounds showed cytotoxicity against P388 and A549, with IC_50_ values of 2.8, 2.1, and 8.8, 4.3 µM [158]. Moreover, 10,11-dihydrobisvertinolone (**267**), isolated from *Trichoderma* sp., showed cytotoxicity against HL-60, with an IC_50_ value of 49.0 µM; a positive control (VP16) inhibited HL-60, with an IC_50_ value of 2.1 µM [102]. The deep-sea-derived *Penicillium* sp. F23−2 produced three new nitrogen-containing sorbicillinoids named sorbicillamines B−D (**268**–**270**). These compounds displayed cytotoxic activity against HeLa, BEL-7402, HEK-293, HCT-116, and P388 cell lines, with IC_50_ values greater than 10 µM [159]. *Penicillium terrestre* produced two new chlorinated sorbicillinoids named chloctanspirones A (**271**) and B (**272**). Chloctanspirone A (**271**) was active against both HL-60 and A-549 cells, with IC_50_ values of 9.2 and 39.7 µM, respectively, while chloctanspirone B (**272**) showed weaker activity only against HL-60 cells, with an IC_50_ of 37.8 µM [160]. Chaetomugilins G–H (**273**–**274**) [161], I–L (**275**–**278**) (Figure 11) [50], and *seco*-chaetomugilins D (**279**) [162] were isolated from *Chaetomium globosum* OUPS-T106B-6. All the compounds could inhibit the proliferation of various tumor cells, including P388, HL-60, L1210, and KB. One new metabolite (**280**) was isolated from a mangrove endophytic fungus, *Penicillium* 303#. This compound showed cytotoxic activities against HCT-116, HepG2, and A549, with IC_50_ values ranging from 11.09 to 24.62 µg/mL, while a positive control (epirubicin) inhibited these cell lines, with IC_50_ values ranging from 0.28 to 0.6 µg/mL [163]. Penicilazaphilone C (**281**), an azaphilonidal derivative, was isolated from *Penicillium sclerotiorum* M-22, which was isolated from a rotten leaf. Penicilazaphilone C (**281**) was selective against melanoma cells B-16 and human gastric cancer cells SGC-7901, with IC_50_ values of 0.065 and 0.720 mM, respectively [164]. *Rhizopus* sp. 2-PDA-61 yielded a new pyran derivative named aspericin C (**282**), which exhibited cytotoxicity against P388, HL-60, and BEL-7402, with IC_50_ values of 14.6, 7.1, and 24.2 µM, while VP16, a positive control, inhibited these cell lines, with IC_50_ values ranging from 0.064 to 1.025 μM [165].

Moreover, 7-epiaustdiol (**283**) and 8-*O*-methyl-epiaustdiol (**284**) were isolated from the mangrove endophytic fungus *Talaromyces* sp. ZH-154. These compounds showed cytotoxicity, with IC_50_ values of 20.04 ± 1.26, 16.37 ± 0.54 against KB, and 19.32 ± 0.60, 37.16 ± 1.43 against KBv200 μg/mL; compared to a positive control (cisplatin), it was 0.56 ± 0.18 and 0.78 ± 0.23 μg/mL [166]. The saline soil-derived fungus *Penicillium raistrickii* yielded peneciraistin C (**285**), which exhibited moderate cytotoxic activity against MCF-7 and A549, with IC_50_ values of 7.6 and 3.2 μM, which are stronger than that of the positive control, fluorouracil (with IC_50_ values of 9.3 and 11.2 μM) [167]. Sorbicillamine E (**286**) was isolated from *Penicillium* sp. F23−2. This compound displayed cytotoxic activity against HeLa, BEL-7402, HEK-293, HCT-116, and P388 cell lines, with IC_50_ > 10 µM [159]. A new decaline derivative, decumbenone C (**287**), was isolated from *Aspergillus sulphureus* KMM 4640. The compound displayed potent cytotoxic activity against SK-MEL-5 human melanoma cells, with an IC_50_ value of 0.9 µM [168]. Penicillone A (**288**) was isolated from *Penicillium* sp. F11. The compound showed cytotoxicity against Cne2 and HT1080 cell lines, with IC_50_ values of 46.2 and 45.8 μM, respectively [169]. Using a modified diethyl sulphate mutagenesis procedure on *Penicillium purpurogenum* G59 could yield penicimutanolone (**289**). This compound inhibited HeLa, K562, HL-60, BGC-823, and MCF-7 human cancer cell lines, with IC_50_ values of 10.9, 17.4, 4.2, 12.6, and 8.6 μM [170]. Isariketide (**290**) was isolated from the marine-sediment-derived fungus *Isaria feline* KMM 4639. This compound displayed cytotoxicity against HL-60 and THP-1, with IC_50_ values of 4.3 and 37.4 µM compared to a positive control (cisplatin), with IC_50_ values of 2.28 and 80.6 [171]. Sorbicillfuran B (**291**), a novel sorbicillinoid adduct containing bicyclo [2.2.2] octane and tetrahydrofuran moieties, was isolated from *Penicillium citrinum* SCSIO41402. Sorbicillfuran B (**291**) exhibited weak cytotoxicity against HL-60 cells, with an IC_50_ value of 9.6 μM [172]. Euroticin I (**292**), isolated from *Eurotium* sp. SCSIO F452, exhibited cytotoxicity against SF-268, MCF-7, HepG-2, and A549, with IC_50_ values ranging from 12.74 to 23.73 µM [133].

#### 2.1.4. Linear Polyketides

**Figure 12:**
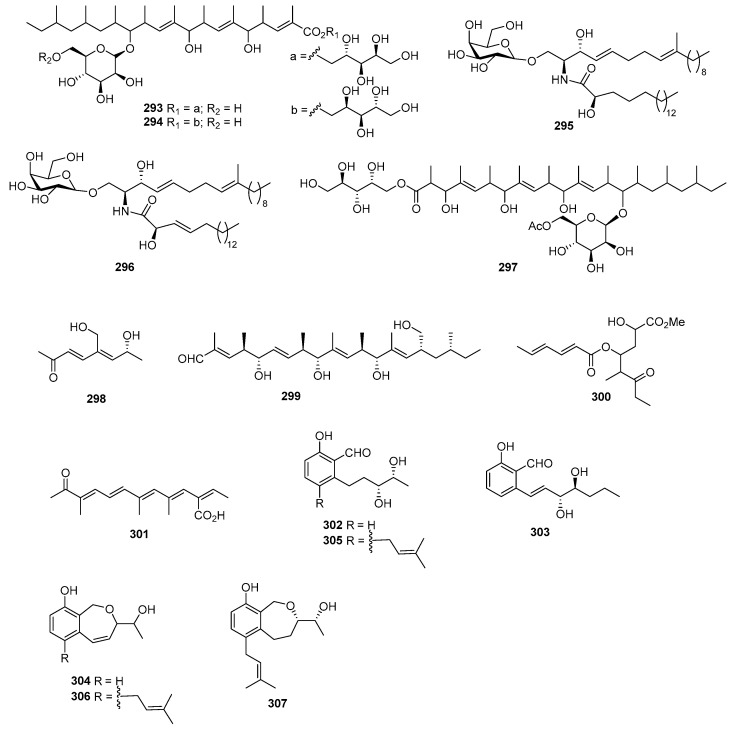
Structures of compounds **293**–**307**.

*Gliocladium roseum* KF-1040 produced roselipins 1A (**293**) (Figure 12) and 1B (**294**), demonstrating cytotoxicity against Raji cells, with a mean IC_50_ value of 39 µM [173,174,175]. Flavicerebrosides A (**295**) and B (**296**) were isolated from *Aspergillus flavipes*, found in the sea anemone *Anthopleura xanthogrammica*, exhibiting cytotoxic activity against the KB cell line, with IC_50_ values of 20.7 and 14.3 µg/mL [176]. Cladionol A (**297**), a novel polyketide glycoside from *Gliocladium* sp. L049 isolated from sea grass *Syringodium isoetifolium*, displayed activity against L1210 and KB cells, with IC_50_ values of 5 and 7 μg/mL, respectively [177]. *Aspergillus* sp. 16-02-1, isolated from deep-sea sediment, produced epiaspinonediol (**298**), with significant cytotoxic activities against K562, HL-60, HeLa, and BGC-823 human cancer cell lines at 100 µg/mL, showing inhibitory rates of 79.7%, 72.5%, 14.9%, and 21.8% [23]. *Pestalotiopsis clavispora*, isolated from the Mangrove plant *Rhizophora harrisonii*, yielded the polyketide derivative pestalpolyol I (**299**), with cytotoxicity against the mouse lymphoma cell line L5178Y, exhibiting an IC_50_ value of 4.10 µM, which was comparable to that of the positive control, kahalalide F (4.3 µM) [178]. Triacremoniate (**300**), isolated from mangrove-derived fungus *Acremonium citrinum*, showed cytotoxicity against HeLa cells, with an IC_50_ value of 30.46 ± 1.99 µM compared to 15.82 ± 0.30 µM for cisplatin (the positive control) [179]. Kaneoheoic acid I (**301**), isolated from *Fusarium graminearum* FM1010, displayed cytotoxic activities against the ovarian cancer cell line A2780 and TNF-α-induced NF-κB inhibitory activity, with IC_50_ values of 18.52 and 15.86 μM, respectively [180]. *Pestalotiopsis heterocornis* XWS03F09 yielded six novel polyketide derivatives named heterocornols A–C (**302**–**304**) and F–H (**305**–**307**). These compounds displayed cytotoxic activities against BGC-823, H460, PC-3, and SMMC-7721, with IC_50_ values ranging from 18.7 to 83.5 μM, whereas adriamycin, as the positive control, inhibited these cell lines, with IC_50_ values of 1.48, 0.98, 1.80, and 2.24 μM [181].

### 2.2. Peptides

#### 2.2.1. Diketopiperazine

**Figure 13:**
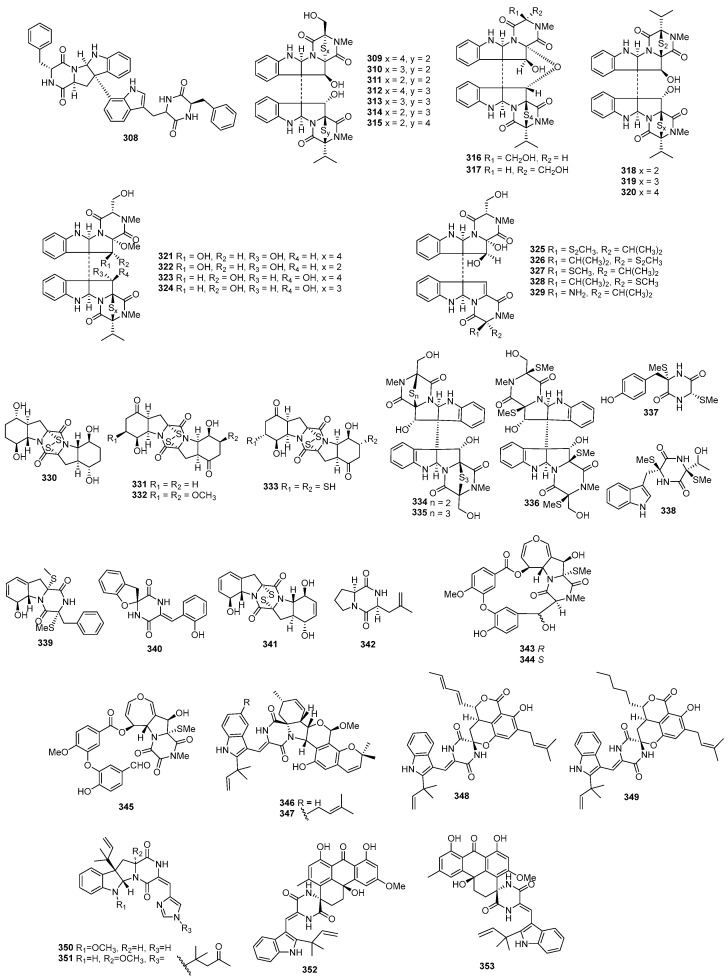
Structures of compounds **308**–**353**.

Asperazine (**308**) (Figure 13), obtained from a sponge-derived culture of *Aspergillus niger*, demonstrated selective activity in a primary in vitro assay at 50 µg/disk, targeting human leukemia murine colon 38, and human colon H116 or CX1 cell lines [182]. Leptosins A–C (**309**–**311**) [183], leptosins G (**312**), G1 (**313**), G2 (**314**), and H (**315**) [184], leptosins I–J (**316**–**317**) [185], leptosins K (**318**), K1 (**319**), and K2 (**320**) [186], leptosins M (**321**), M1 (**322**), N (**323**), and N1 (**324**) [187], and leptosins O–S (**325**–**329**) [188], isolated from *Leptosphaeria* sp. OUPS-4, found in the marine alga *Sargassum tortile*, exhibited strong cytotoxicity against P388, with ED_50_ values of 1.85, 2.40, 1.75, 4.6, 4.3, 4.4, 3.0, 1130, 1250, 3.8, 2.2, 2.1, 1050, 1400, 180, 190, 1100, 100, 14,800, 15,200, and 10,100 ng/mL, respectively, whereas 5-fluorouracil (positive control) inhibited P388, with an IC_50_ value of 0.058 μg/mL. Compound **321** also demonstrated cytotoxicity against 39 other human tumor cell lines, with a mean logGI_50_ of -5.25. Four new cytotoxic disulfides, rostratins A–D (**330**–**333**), were isolated from *Exserohilum rostratum* CNK-630, displaying cytotoxic activities against HCT-116, with IC_50_ values of 8.5, 1.9, 0.76, and 16.5 µg/mL [189]. *Oidiodendron truncatum* GW3-13 produced two new epipolythiodioxopiperazines, chetracins B (**334**), C (**335**), and a new diketopiperazine, chetracin D (**336**), showing cytotoxicity against BEL-7402, BGC-823, HCT-8, A549, and A2780, with IC_50_ values of 0.003–1.83 μM. Concurrently, a positive control (paclitaxel) inhibited HCT-8, Bel-7402, and A549, with IC_50_ values of 0.051, 0.006, and 0.016 μM, and with IC_50_ values stronger than 0.001 against BGC-823 and A2780 [190]. Fusaperazines A (**337**) from *Fusarium chlamydosporum* OUPS-N124, separated from the marine alga *Carpopeltis affinis*, exhibited cytotoxic activity against P388, with an ED_50_ value of 22.8 µg/mL [191]. *Gliocladium roseum* OUPS-N132, isolated from a sea hare, produced glioperazine (**338**), showing significant cytotoxicity against P388, with an ED_50_ value of 6.7 µg/mL [192]. A new thiodiketopiperazine, phomazine B (**339**) from *Phoma* sp. OUCMDZ-1847, associated with the mangrove plant *Kandelia candel*, displayed cytotoxic activity against MGC-803, with an IC_50_ value of 8.5 µM, while adriamycin (the positive control) inhibited the cell line, with an IC_50_ value of 0.17 µM [193]. Two new diketopiperazines, spirobrocazine C (**340**) and brocazine G (**341**), were isolated from the mangrove-derived *Penicillium brocae* MA-231. Compound **340** exhibited moderate activities against A2780, with an IC_50_ value of 59 µM, while compound **341** displayed strong activities against A2780 cells and A2780 CisR cells, with IC_50_ values of 664 and 661 nM, which were stronger than that of cisplatin (the positive control), showing IC_50_ values of 1.67 and 12.63 μM, respectively [194]. Penicimutide (**342**), a novel cyclic dipeptide from *Penicillium purpurogenum* G59, selectively inhibited HeLa cells, with an inhibition rate (IR%) of 39.4% at 100 µg/mL, a similar inhibition intensity to that of the positive control, 5-fluorouracil (IR % of 41.4% at 100 µg/mL against HeLa cells) [195]. *Aspergillus nidulans* SD-531 produced three novel emestrin-type thiodiketopiperazines, including didethio-11a-methylthioemestrin (**343**), 7′-epi-didethio-11a-methylthioemestrin (**344**), and 2′′-desmethyl-MPC1001F (**345**). These compounds exhibited cytotoxic activity against the tumor cell line Huh 7.5, with IC_50_ values of 19, 10, and 8 μM, a similar inhibition intensity to that of the positive control (sorafenib), with an IC_50_ value of 8.2 μM [196].

Four undescribed indole diketopiperazine-based hybrids (**346**–**349**) were isolated from *Aspergillus* sp. EGF 15-0-3. Compounds **346** and **347** displayed cytotoxicity against bladder cancer cell lines 5637 and T24, with IC_50_ values of 13.11, 18.47, 41.26, and 47.92 μM, while compounds **348** and **349** were active against hepatoma cell lines HCC-LM3 and 97H, with IC_50_ values of 5.42 and 3.40, and 3.73 and 8.22 μM, respectively [197]. *Penicillium* sp. F23-2, obtained from a deep-ocean sediment, yielded two new diketopiperazines, roquefortines F (**350**) and G (**351**), which showed varying degrees of cytotoxicities against A549, HL-60, BEL-7402, and MOLT-4 [198]. Variecolortins B (**352**) and C (**353**) were isolated from *Eurotium* sp. SCSIO F452, which displayed moderate cytotoxicities, with IC_50_ values of 12.5, as well as values of 30.1 μM against SF-268, and 15.0 and 37.3 μM against HepG2 [199].

#### 2.2.2. Cyclicpetides

**Figure 14:**
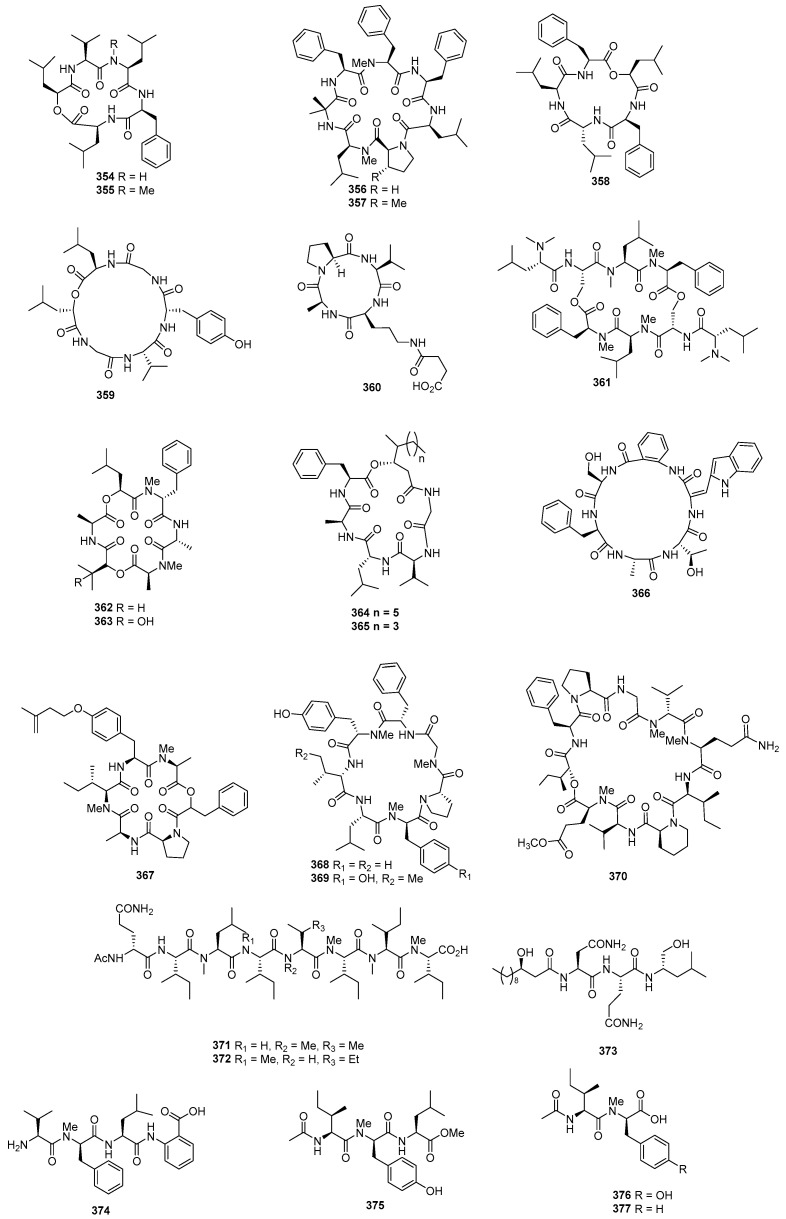
Structures of compounds **354**–**377**.

Sansalvamide A (**354**) (Figure 14), a novel cyclic pentadepsipeptide isolated from *Fusarium* sp. CNL-292, demonstrated selective cytotoxicity against HCT-116, COLO-205, and SK-MEL-2, with IC_50_ values of 9.8, 3.5, and 5.9 µg/mL, respectively [200]. Another cyclic depsipeptide, *N*-Methylsansalvamide (**355**), obtained from *Fusarium* sp. CNL-619, exhibited weak cytotoxicity against the NCI human tumor cell line, with a mean GI_50_ value of 8.3 µM [201]. *Scytalidium* sp. CNC-310 produced two new cyclic heptapeptides, named scytalidamides A (**356**) and B (**357**), showing cytotoxicity against HCT-116, with IC_50_ values of 2.7 and 11.0 µM. Both compounds displayed cytotoxicity against 60 NCI human tumor cell lines, with mean GI_50_ values of 7.9 and 4.1 µM. Additionally, compound **356** exhibited cytotoxicity against MOLT-4, with a GI_50_ value of 3.0 µM, and **357** showed cytotoxicity against Uacc-257, with a GI_50_ value of 1.2 µM [202]. Zygosporamide (**358**), a novel cyclic pentadepsipeptide from *Zygosporium masonii* CNK458, exhibited significant cytotoxicity across the NCI’s 60 cell line panel, with a mean GI_50_ of 9.1 µM. It displayed highly enhanced selectivity against SF-268, with a GI_50_ value of 6.5 nM, and for RXF393, with a GI_50_ value lower than 5.0 nM [203]. A new cyclic depsipeptide 1962A (**359**) from a mangrove endophytic fungus (no. 1962) demonstrated cytotoxic activity against MCF-7, with an IC_50_ value of 100 µg/mL [204]. Trichoderide A (**360**), isolated from *Trichoderma reesei* YZ48-08, inhibited A375-S2 at a concentration of 18.5 µg/mL [205]. *Clonostachys* sp. ESNA-A009 produced a new cytotoxic cyclodepsipeptide with a C_2_ symmetry named IB-01212 (**361**). This compound exhibited cytotoxicity against HeLa, SK-BR3, LN-caP, and HT29, with a GI_50_ value of 10 nM [206]. Two new cyclohexadepsipeptides, spicellamides A (**362**) and B (**363**), isolated from *Spicellum roseum*, showed cytotoxicity against neuroblastoma cells, with IC_50_ values of 30 and 6.2 µg/mL [207]. Scopularides A (**364**) and B (**365**), novel cyclodepsipeptides from *Scopulariopsis brevicaulis* separated from the marine sponge *Tethya aurantium*, exhibited cytotoxic activities against HT29, Colo357, and Panc89, with inhibitory rate values between 24% and 49% at 10 µg/mL [208]. Sclerotide B (**366**), containing both anthranilic acid and dehydroamino acid units, was isolated from *Aspergillus sclerotiorum* PT06-1, showing weak cytotoxicity against HL-60, with an IC_50_ value of 56.1 μM [209]. Pullularin E (**367**), obtained from *Bionectria ochroleuca* isolated from the inner leaf tissues of the plant *Sonneratia caseolaris* from Hainan Island, exhibited activity against the mouse lymphoma cells L5178Y, with an EC_50_ value of 5.6 µg/mL, which is comparable to the positive control (kahalalide F), with an IC_50_ value of 6.4 µg/mL [210]. Cordyheptapeptides C (**368**) and E (**369**), isolated from *Acremonium persicinum* SCSIO 115, demonstrated cytotoxicity against SF-268, MCF-7, and NCI-H460 tumor cell lines, with IC_50_ values ranging from 2.5–12.1 μM whereas the positive control (cisplatin) with IC_50_ values ranging from 1.6 to 10.2 μM [211]. A novel cyclodecadepsipeptide, phaeosphamide A (**370**), isolated from mangrove-derived fungus *Phaeosphaeriopsis* sp. S296, exhibited inhibitory activities against AGS, BEL-7402, HepG2, B16, and BIU87 cell lines, with IC_50_ values ranging from 5.14 to 66.38 μM [212].

#### 2.2.3. Linear Peptides

RHM1 (**371**) and RHM2 (**372**) are highly *N*-methylated linear octapeptides isolated from an atypical strain of *Acremonium* sp. These compounds showed limited cytotoxicity against the murine cancer cell line L1210 [213]. Another compound, fellutamide C (**373**), was extracted from *Aspergillus versicolor* and demonstrated cytotoxic activity against XF498, SK-MEL-2, and HCT15, with IC_50_ values of 3.9, 5.1, and 3.1 μM, while doxorubicin (the positive control) inhibited these cell lines, with IC_50_ values of 0.22, 0.07 and 0.33 μM, respectively [214]. From the deep-sea-derived fungal strain *Simplicillium obclavatum* EIODSF 020, four novel linear peptides named simplicilliumtides A, E, G, and H (**374**–**377**) were obtained. Among these, compounds **374** and **376** exhibited weak cytotoxicity against the human leukemia HL-60 cell line, with IC_50_ values of 64.7 and 100 µM. Additionally, compounds **375** and **377** displayed weak cytotoxicity towards the K562 cell line, with IC_50_ values of 39.4 and 73.5 µM [215].

### 2.3. Terpenoids and Sterols

#### 2.3.1. Sesquiterpenoids

**Figure 15:**
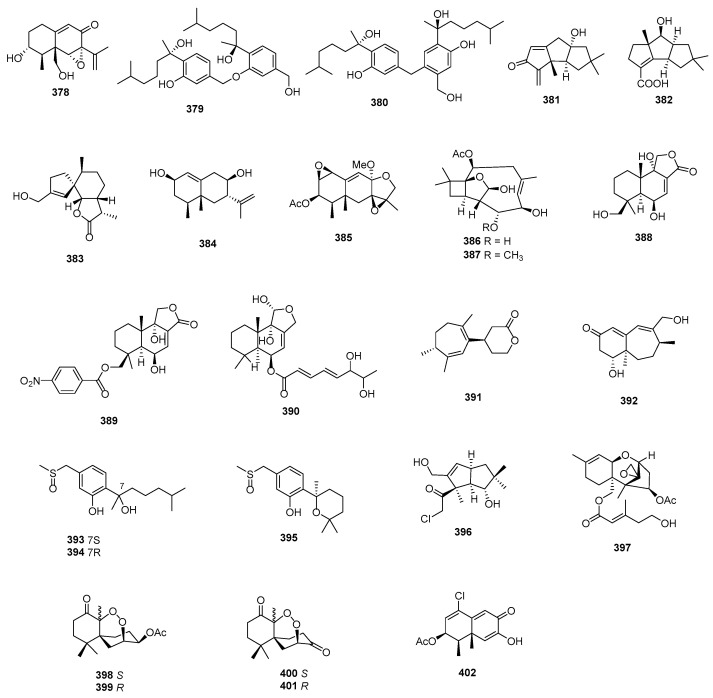
Structures of compounds **378**–**402**.

*Penicillium* sp. SS080624SCf1 produced a novel sesquiterpene named JBIR-28 (**378**) (Figure 15), exhibiting cytotoxicity against the human cervical carcinoma cell line HeLa, with an IC_50_ value of 92 μM [216]. *Aspergillus* sp. yielded two phenolic bisabolane sesquiterpenoid dimers, disydonols A (**379**) and C (**380**). These compounds were tested for cytotoxic activity against HepG-2 and Caski human tumor cell lines, displaying IC_50_ values of 9.31, 2.91 µg/mL against HepG-2, and 12.40 and 10.20 µg/mL against Caski, respectively [217]. *Chondrostereum* sp. nov. SF002, isolated from a soft coral *Sarcophyton tortuosum* in the South China Sea, produced a new triquinane-type sesquiterpenoid named chondrosterin A (**381**). This compound demonstrated cytotoxicity against A549, CNE2, and LoVo, with IC_50_ values of 2.45, 4.95, and 5.47 µM [218]. Additionally, chondrosterin J (**382**) from the same fungi exhibited potent cytotoxic activities against the cancer cell lines CNE-1 and CNE-2, with IC_50_ values of 1.32 and 0.56 μM [219]. *Penicillium* sp. FJ-1 yielded a new compound, 15-hydroxy-6α,12-epoxy-7β,10α*H*,11β*H*-spiroax-4-ene-12-one (**383**), with IC_50_ values of 10 µM against Tca8113, 58 µM against the normal liver cell line WRL-68, and an antitumor effect on MG-63 cells with an IC_50_ value of 55 nM, whereas taxol (the positive control) inhibited Tca8113 and MG-63, with IC_50_ values of 46 and 10 nM, respectively [220]. *Penicillium* sp. PR19 N-1, an Antarctic deep-sea-derived fungus, produced two new eremophilane-type sesquiterpenes (**384**–**385**) with cytotoxicity, with IC_50_ values of 82.8, 5.2 against A-549, and 45.8 and 28.3 against HL-60 µM [221]. *Ascotricha* sp. ZJ-M-5 yielded two new caryophyllene derivatives (**386** and **387**) with a five-membered hemiacetal structural moiety. These compounds showed activity with GI_50_ values of 10.1, 12.3 µM against K562, and 6.9 and 8.5 µM against HL-60, which were greater than the positive control (cisplatin), with GI_50_ values of 19.1 and 13.4 µM [222]. *Aspergillus flocculosus*, a Vietnamese marine-sediment-derived fungus, produced a new drimane derivative (**388**) displaying cytotoxic activity against murine neuroblastoma Neuro-2a and human prostate cancer 22Rv1, with IC_50_ values of 24.1 and 31.5 µM, while docetaxel (the positive control) inhibited 22Rv1, with an IC_50_ value of 0.02 µM [223]. A new nitrobenzoyl sesquiterpenoid, 6β,9α-dihydroxy-14-p-nitrobenzoylcinnamolide (**389**), was isolated from *Aspergillus ochraceus* Jcma1F17, showing significant cytotoxicity against various cancer cell lines, including H1975, U937, K562, BGC-823, Molt-4, MCF-7, A549, Hela, HL60, and Huh-7 (with IC_50_ values from 1.95 to 6.35 μM) [224]. *Penicillium chrysogenum* LD-201810 yielded a drimane sesquiterpene ester named chrysoride A (**390**), which displayed moderate cytotoxicity against HeLa and HepG2 cancer cell lines, with IC_50_ values of 35.6 and 28.9 μM, respectively [225]. Lastly, two new compounds, nigerin (**391**) and ochracene J (**392**), isolated from *Aspergillus niger*, exhibited potent inhibitory activity on the production of nitric oxide (NO) in lipopolysaccharide (LPS)-stimulated RAW264.7 macrophages, with IC_50_ values of 8.5 and 4.6 μM [226]. A pair of new enantiomers, (+)-**393** and **394**, and a new derivative (**395**) were produced by *Aspergillus flavipes* 297. Compounds **393** and **394** exhibited cytotoxicity, with IC_50_ values of 39.9, 43.3 µg/mL against HepG2, and 28.7 and 30.1 µg/mL against MKN-45, while compound **395** only inhibited HepG2, with an IC_50_ value of 19.8 µg/mL [227]. A new chlorinated, cyclic sesquiterpene, chloriolin A (**396**), was isolated from an unidentified fungus separated from the marine sponge *Jaspis* aff. *Johnstoni*. The new compound showed cytotoxicity against SNB-75 and T-47D, with IC_50_ values of 0.5 and 0.7 µM, respectively [228]. Compound **397**, a new cytotoxic trichothecene sesquiterpene, was isolated from *Acremonium neocaledoniae*. The compound displayed cytotoxicity against KB, with an IC_50_ value of 0.4 μg/mL [229]. *Talaromyces flavus* produced talaperoxides A–D (**398–401**), two new norsesquiterpene peroxides. Compounds **399** and **401** exhibited cytotoxicity against MDA-MB-435, HeLa, MCF-7, HepG2, and PC-3, with IC_50_ values ranging from 0.70 to 2.78 μg/mL, which was stronger than compounds **398** and **400**, with IC_50_ values ranging from 2.64 to 19.77 μg/mL [230]. An Antarctic deep-sea-derived fungus *Penicillium* sp. PR19N-1 yielded a new chlorotrinoreremophilane sesquiterpene (**402**), which showed moderate cytotoxic activity against A549 and HL-60, with IC_50_ values of 12.2 and 11.8 µM [231].

#### 2.3.2. Diterpenoids

**Figure 16:**
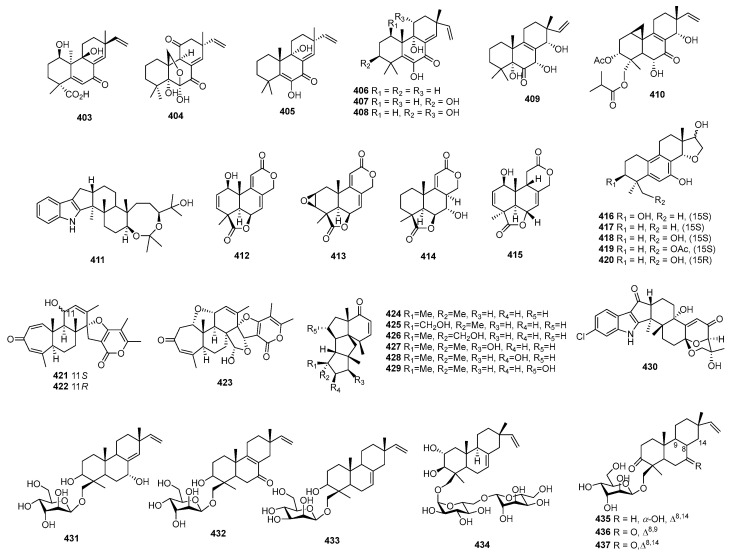
Structures of compounds **403**–**437**.

Myrocin D (**403**) (Figure 16), obtained from *Arthrinium* sp. 9287, isolated from the Mediterranean sponge *Geodia cydonium*, exhibited cytotoxicity against K562, L5178Y, A2780CisR, and A2780, with IC_50_ values of 50.3, 2.05, 66.0, and 41.3 µM compared to sunitinib (with an IC_50_ value of 0.12 µM) [232]. *Epicoccum* sp. HS-1 produced a new pimarane diterpenes (**404**–**405**), with IC_50_ values of 3.51, 20.74 µg/mL against KB, and 2.34 and 14.47 µg/mL against KBv200 [233]. Four new oxygenated pimarane diterpenes, scopararanes C–E and G (**406**–**409**), were isolated from *Eutypella scoparia* FS26 collected from the South China Sea. These compounds exhibited activity against MCF-7, with IC_50_ values of 35.9, 25.6, 74.1, and 85.5 μM. Additionally, compound **407** displayed cytotoxicity against SF-268 and NCI-H460, with IC_50_ values of 43.5 and 46.1 μM, whereas cisplatin (the positive control) inhibited SF-268, MCF-7, and NCI-H460, with IC_50_ values of 4.0, 9.2, and 1.5 μM, respectively [234]. Another new pimarane-type diterpene, scopararane I (**410**), was identified from *Eutypella* sp. FS46. Compound **410** demonstrated activity against MCF-7, NCI-H460, and SF-268 tumor cell lines, with IC_50_ values of 83.91, 13.59, and 25.31 µg/mL [235]. The *Penicillium brefeldianum* strain WZW-F-69 produced a novel indole diterpenoid named paspaline C (**411**). This compound displayed inhibition rates of 55.1%, 56.1%, 56.4%, 71.2%, and 65.8% against HepG-2, U2OS, MCF7, JeKo-1, and HL-60 cell lines at a concentration of 1 µM [236].

*Aspergillus wentii* EN-48 yielded tetranorlabdane diterpenoids, asperolides A−B (**412**−**413**), which were evaluated for cytotoxic activity against several tumor cells, with IC_50_ values from 35 to 97 μM [237]. Botryosphaerin F (**414**) was isolated from the mangrove fungus *Aspergillus terreus* GX73B. This novel compound showed potent inhibitory activity towards MCF-7 and HL-60 cancer cell lines, with a 50% inhibition of cell growth, with IC_50_ values of 4.49 and 3.43 µM compared to the positive control epirubicin (with IC_50_ values of 0.98 and 0.71 µM) [238]. A novel tetranorlabdane diterpenoid, known as asperolide E (**415**), was isolated from the fungus *Aspergillus wentii* SD-310, which was derived from deep-sea sediment. Compound **415** was assessed for its cytotoxicity against HeLa, MCF-7, and NCI-H446 cell lines, revealing IC_50_ values of 10.0, 11.0, and 16.0 µM, respectively [239]. Five new 20-nor-isopimarane diterpenoids having a 14,16-cyclic ether unit and possessing a unique 6/6/6/5 tetracyclic skeleton, named asperethers A–E (**416**–**420**), isolated from *Aspergillus wentii* SD-310, showed cytotoxic activities against the A549 cell line, with IC_50_ values of 20, 16, 19, 17, and 20 µM, while adriamycin as the positive control inhibited A549, with an IC_50_ value of 8 µM [240]. Three new bioactive breviane spiroditerpenoids named breviones F–H (**421**–**423**) were isolated from *Penicillium* sp., which were purified from a deep-sea sediment sample. Breviones F–H (**421**–**423**) were evaluated against HeLa cells and displayed inhibitory effects of 25.2%, 44.9%, and 25.3% at 10 µg/mL, respectively [241]. *Penicillium* sp. F23-2, obtained from a deep-ocean sediment, yielded conidiogenone B–G (**424–429**), which showed varying degrees of cytotoxicities against A549, HL-60, BEL-7402, and MOLT-4, while compound **425** inhibited BEL-7402 and HL-60 cell lines, with IC_50_ values of 0.97 and 0.038 μM [198]. Ascandinine D (**430**), one novel indole diterpenoid, was isolated from an Antarctic sponge-derived fungus *Aspergillus candidus* HDN15-152, which displayed cytotoxicity against HL-60 cells, with an IC_50_ value of 7.8 μM, while adriamycin, as the positive control, inhibited HL-60, with an IC_50_ value of 0.02 µM [242]. *Acremonium striatisporum* KMM 4401, initially isolated from the holothurian *Eupentacta fraudatrix*, produced virescenosides O–Q (**431**–**433**) [243] and R–U (**434**–**437**) [244]. Compounds **432** and **434**–**437** showed cytotoxicity against various cancer cell lines, with IC_50_ values ranging from 5.0 to 150 µM. Moreover, compounds **431**–**437** demonstrated the ability to inhibit Ehrlich carcinoma, with IC_50_ values ranging from 20 to 100 µM [243,244].

#### 2.3.3. Sesterterpenoids

**Figure 17:**
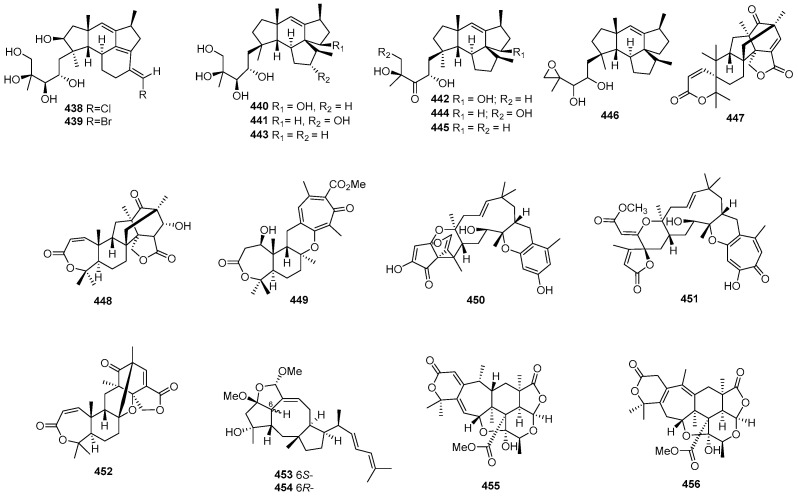
Structures of compounds **438**–**456**.

*Fusarium heterosporum* CNC-477 produced neomangicol A (**438**) (Figure 17) and B (**439**) [245], and mangicols A–G (**440**–**446**) [246]. Compound **438**–**439** had a previously undescribed carbon skeleton, representing a novel class of C_25_ rearranged sesterterpenes. Compounds **440**–**446** were structurally unique sesterterpene polyols. Compound **438** displayed activity against MCF-7 and CACO-2, with IC_50_ values of 4.9 and 5.7 µM, which was stronger than the mean IC_50_ value of 27 µM, while compounds **440**–**446** exhibited IC_50_ values ranging from 18 to 36 µM against 60 cell lines. *Aspergillus* CNK-371 yielded tropolactones A–C (**447**–**449**) containing an intriguing substituted 2,4,6-cycloheptatriene (tropone) ring. These compounds demonstrated in vitro cytotoxicity against human colon carcinoma (HCT-116), with IC_50_ values of 13.2, 10.9, and 13.9 μg/mL [247]. Phomeroids A (**450**) and B (**451**), two novel meroterpenoids representing two types of skeletons, were isolated from the deep-sea-derived fungus *Phomopsis tersa* FS441. These compounds showed significant cytotoxicity against SF-268, MCF-7, HepG-2, and A549, with IC_50_ values of 14.2, 12.0, 11.7, and 17.6 µM for **450** and 0.50, 1.30, 1.00, and 1.10 µM for **451**. Meanwhile, adriamycin as a positive control inhibited these cell lines, with IC_50_ values ranging from 1.1 to 1.5 µM [248]. A novel meroterpenoid, insuetolide C (**452**), isolated from *Aspergillus insuetus* (OY-207) from the Mediterranean sponge *Psammocinia* sp., exhibited mild cytotoxicity against MOLT-4 human leukemia cells, with an inhibition rate (IR%) of 51% at 50 mg/mL [249]. Two new sesterterpenes, ophiobolin O (**453**) and 6-*epi*-ophiobolin O (**454**), were isolated from the marine-derived fungus *Aspergillus* sp. 094102. These compounds demonstrated strong cytotoxicity against P388, with IC_50_ values of 4.7 and 9.3 μM [250]. Meroterpenes (**455**–**456**) were isolated from the marine fungus *Penicillium* sp. 303#, separated from seawater in Zhanjiang Mangrove National Nature Reserve, Guangdong Province, China. These compounds showed moderate cytotoxic activities against various cancer cell lines, including MDA-MB-435, HepG2, HCT-116, and A549, with IC_50_ values of 34.25, 24.56, 33.72, and 37.82 μg/mL, and 31.32, 23.87, 29.19, and 34.06 μg/mL [163].

#### 2.3.4. Sterols

**Figure 18:**
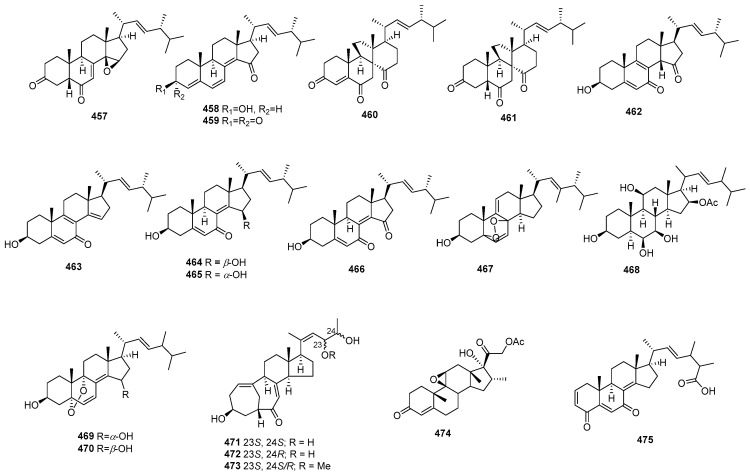
Structures of compounds **457**–**475**.

The fungus *Gymnacella dankaliensis*, derived from the Halichondria sponge, produced novel compounds: gymnasterone B–D (**457**–**459**) (Figure 18) and dankasterones A (**460**) and B (**461**) [251,252]. These compounds demonstrated cytotoxicity against P388, with ED_50_ values ranging from 0.9 to 2.5 µg/mL, respectively. Six new ergosterols (**462**–**467**) were isolated from the marine-derived fungus *Rhizopus* sp., exhibiting stronger inhibition against P388 and HL-60 compared to A549 and BEL-7402. The cytotoxic activities against P388 and HL-60 showed IC_50_ values ranging from 14 to 9.3 and 1.3 to 7.1 µM [253]. *Penicillium chrysogenum* QEN-24S, an endophytic fungus from an unidentified marine red algal species, yielded the polyoxygenated steroid penicisteroid A (**468**). This structurally unique steroid with tetrahydroxy and C-16-acetoxy groups displayed cytotoxicity against NCI-H460, Hela, and SW1990 cells, with IC_50_ values of 40, 15, and 31 µg/mL [254]. *Aspergillus niger* MA-132, an endophytic fungus from the mangrove plant *Avicennia marina*, produced two new 6,8(14),22-hexadehydro-5a,9a-epidioxy-3,15-dihydroxy sterols, named nigerasterols A (**469**) and B (**470**). These compounds showed cytotoxic activity, with IC_50_ values of 1.82, 5.41 µM against A549, and 0.30 and 1.50 µM against HL-60 [255]. Three new C_25_ steroids (**471**–**473**) with an unusual bicyclo [4.4.1] A/B ring system were isolated from an antitumor mutant AD-1-2 of marine-derived *Penicillium purpurogenum* G59. These compounds exhibited cytotoxicity against HL-60 and K562, with an inhibition ranging from 13.3 to 34.7% at a tested concentration of 100 µg/mL [256]. *Penicillium citrinum* SCSIO 41017, associated with the sponge *Callyspongia* sp., produced a new steroid named 16a-methylpregna-17a,19-dihydroxy-(9,11)-epoxy-4-ene-3,18-dione-20-acetoxy (**474**). Compound **474** exhibited moderate activity against SF-268, MCF-7, HepG-2, and A549, with IC_50_ values ranging from 13.5 to 18.0 μM [257]. A novel oxygenated steroid, aspersteroid A (**475**), was isolated from *Aspergillus flavus* YJ07-1, showing selective cytotoxicity against the A-549 cell line, with an IC_50_ value of 14.6 μM [258].

### 2.4. Hybrids

#### 2.4.1. Hybrids of Polyketides and Peptides (or Amino Acids)

**Figure 19:**
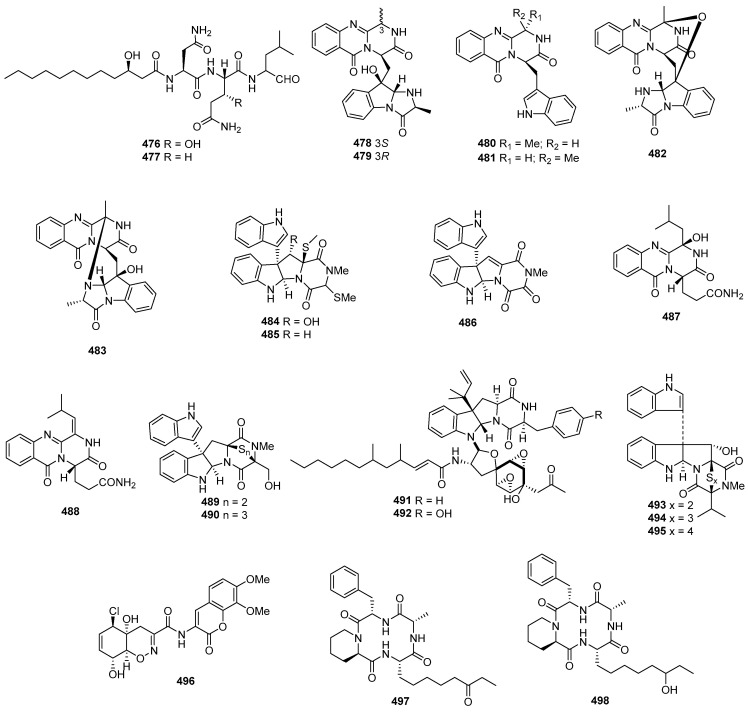
Structures of compounds **476**–**498**.

**Figure 20:**
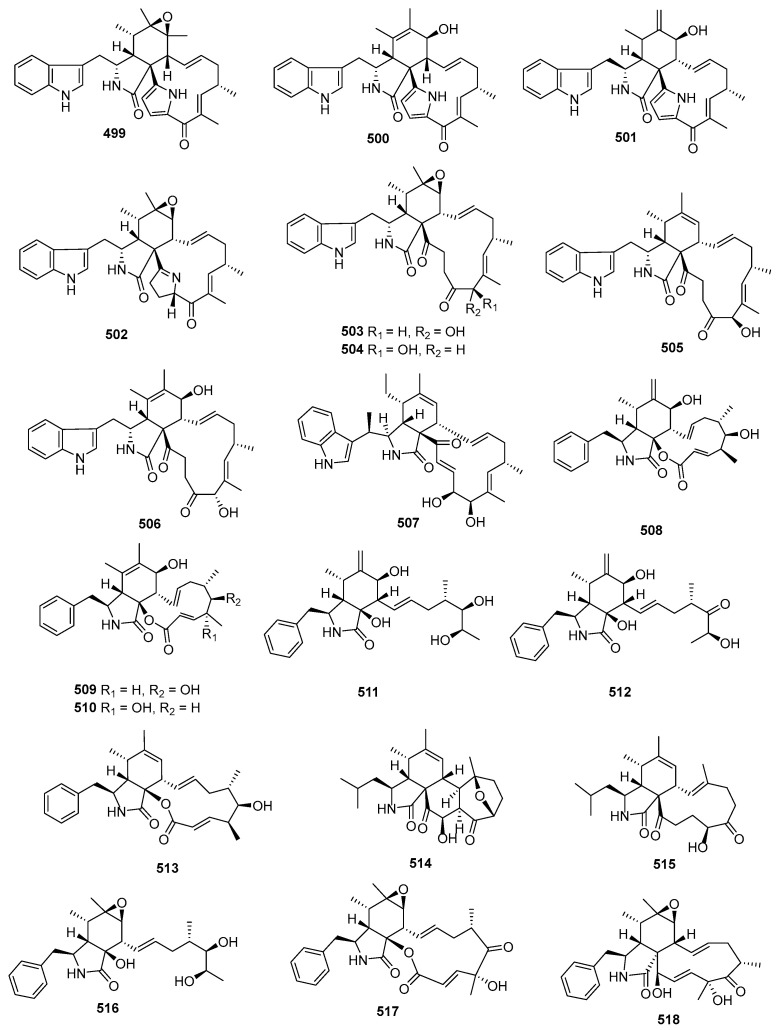
Structures of compounds **499**–**518**.

Cytotoxic peptides, fellutamides A (**476**) (Figure 19) and B (**477**), were isolated from *Penicillium fellutanum* Btourge, found in the gastrointestinal tract of the marine fish *Apogon endekataenia* Bleeker. These two compounds (**476** and **477**) exhibited cytotoxicity against P388, L1210, and KB cells, with IC_50_ values of 0.2 and 0.1, 0.8 and 0.7, and 0.5 and 0.7 µg/mL [259]. *Aspergillus fumigatus* produced fumiquinazolines A (**478**), B (**479**), and D–G (**480**–**483**), displaying moderate cytotoxicity against P388, with ED_50_ values of 6.1, 16.0, 13.5, 13.8, 14.6, and 17.7 μg/mL [260]. *Gliocladium roseum* OUPS-N132, isolated from the sea hare, yielded gliocladins A−C (**484**−**486**). Gliocladins A (**484**) and B (**485**) exhibited cytotoxicity against P388, with ED_50_ values of 6.5 and 20 µg/mL, while compound **486** showed more potent activity against P388, with an ED_50_ of 2.4 µg/mL [192]. Two new quinazoline alkaloids, aurantiomides B (**487**) and C (**488**), were isolated from the sponge-derived fungus *Penicillium aurantiogriseum* SP0-19. Aurantiomide B (**487**) exhibited moderate cytotoxicities against P388 and HL-60, with IC_50_ values of 54 and 52 µg/mL. Aurantiomide C (**488**) showed cytotoxicity against P388 and BEL-7402, with IC_50_ values of 48 and 62 µg/mL [261]. Luteoalbusins A (**489**) and B (**490**), two new indole diketopiperazines, were isolated from *Acrostalagmus luteoalbus* SCSIO F457, originally purified from deep-sea sediment. Compounds **489** and **490** showed cytotoxicity against MCF-7, NCI-H460, SF-268, and HepG-2, with IC_50_ values of 0.23–1.31 μM, which was stronger than cisplatin, with IC_50_ values of 2.45–4.76 μM [262]. Using a modified diethyl sulfate mutagenesis procedure on *Penicillium purpurogenum* G59 yielded penicimutanins A (**491**) and B (**492**). These compounds inhibited HeLa, K562, HL-60, BGC-823, and MCF-7 human cancer cell lines, with IC_50_ values of 9.5/17.7, 11.4/19.9, 5.4/12.1, 8.0/16.6, and 5.4/8.0 μM [170]. Leptosins D–F (**493**–**495**) were isolated from *Leptosphaeria* sp. OUPS-4, separated from the marine alga *Sargassum tortile*. These compounds showed strong cytotoxicity against P388, with ED_50_ values of 86, 46, and 56 ng/mL [183]. Trichodermamide B (**496**), possessing a rare cyclic *O*-alkyl-oxime functionality incorporated into a six-membered ring, was isolated from *Trichoderma virens* CNL910. The novel modified dipeptide exhibited cytotoxicity against HCT-116, with an IC_50_ of 0.32 µg/mL [263]. *Microsporum* cf. *gypseum* CNL-629, separated from a sample of the bryozoan *Bugula* sp. collected in the U.S. Virgin Islands, yielded two new cyclic peptides named microsporins A (**497**) and B (**498**). These compounds showed cytotoxic activity against HCT-116, with IC_50_ values of 0.6 and 8.5 µg/mL. Meanwhile, compound **497** also showed cytotoxic activities against 60 cancer cells, with a mean IC_50_ value of 2.7 µM [264]. A novel class of cytochalasans, penochalasins A–C (**499**–**501**) (Figure 20), was isolated from a strain of *Penicillium* sp. originally separated from the marine alga *Enteromorpha intestinalis*. All the compounds exhibited potent cytotoxicity against cultured P388 cells, with ED_50_ values of 0.4, 0.3, and 0.5 µg/mL [265].

**Figure 21:**
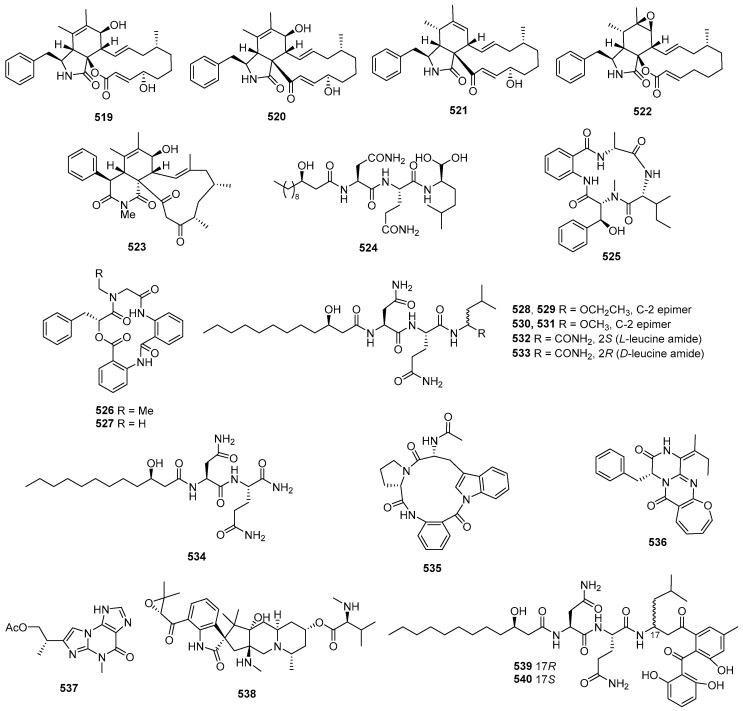
Structures of compounds **519**–**540**.

*Penicillium* sp. OUPS-79, purified from the marine alga *Enteromorpha intestinalis*, yielded five new cytotoxic metabolites designated as penochalasins D–H (**502**–**506**). These compounds displayed moderate cytotoxic activities against P388, with ED_50_ values of 3.2, 2.1, 1.8, 1.9, and 2.8 µg/mL [266]. Chaetoglobosin-542 (**507**) was extracted from *Phomopsis asparagi*, demonstrating weak cytotoxicity against C38, L1210, and CFU-GM [267]. *Spicaria elegans* KLA03 produced compounds **508**–**510** [268], **511**–**512** [269], **513** [270], **514**–**515** [271], and **516**–**517** [272]. Among these, compounds **508**–**513** and **516**–**517** exhibited cytotoxic activity against A549, with IC_50_ values ranging from 4.3 to 21.0 μM. Compounds **508**–**510** displayed cytotoxic effects on P388, with IC_50_ values of 56–99 μM, and compounds **514**–**515** showed cytotoxicity against HL-60, with IC_50_ values of 19.9 and 20.0 μM. *Xylaria* sp. SCSIO156, from the South China Sea marine sediment, produced 21-*O*-deacetylcytochalasin Q (**518**), with weak cytotoxic activity against SF-268 and NCF-H460 (with IC_50_ values of 44.3 and 96.4 μM) [273]. Two new cytochalasin derivatives, deoxaphomins B (**519**) (Figure 21) and C (**520**), were isolated from the fungus *Phoma* sp. from the giant jellyfish *Nemopilema nomurai*. Compounds **519**–**522** displayed cytotoxicity against SK-MEL-2, SK-OV-3, A549, HCT15, and XF498, with IC_50_ values ranging from 4.19 to 29.32 µM [274]. The cytochalasan asporychalasin (**523**) was isolated from *Aspergillus oryzae* in the Red Sea sediments off Jeddah, Saudi Arabia, showing moderate cytotoxic activity against A549, HepG2, and MCF-7, with IC_50_ values of 8.8 ± 0.4, 7.4 ± 0.2, and 8.3 ± 0.3 μg/mL, respectively [275]. *Aspergillus versicolor*, isolated from a marine sponge *Petrosia* sp., produced fellutamide F (**524**), exhibiting cytotoxicity against A549, SK-OV-3, SK-MEL-2, XF498, and HCT15, with ED_50_ values ranging from 0.13 to 1.81 µg/mL, while doxorubicin (the positive control) inhibited these cell lines, with ED_50_ values ranging from 0.01 to 0.18 µg/mL [276]. *Aspergillus terreus* SCSGAF0162 produced asperterrestide A (**525**), a novel compound with cytotoxicity against U937 and MOLT4 human carcinoma cell lines, having IC_50_ values of 6.4 and 6.2 μM [277]. The fungus *Aspergillus clavatus* C2WU was found to produce clavatustides A (**526**) and B (**527**), which demonstrated the dose-dependent suppression of hepatocellular carcinoma (HCC) cell lines (HepG2, SMMC-7721, and Bel-7402). These compounds induced cell-cycle arrest in the G1 phase and reduced cells in the S phase [278]. Another fungus, *Penicillium purpurogenum* G59, yielded seven new lipopeptides named penicimutalides A–G (**528**–**534**), exhibiting cytotoxicity against various cancer cell lines, including K562, HL-60, HeLa, BGC-823, and MCF-7 [279].

In a mixed culture of two marine-alga-derived fungal strains of the genus *Aspergillus*, a new cyclotripeptide named psychrophilin E (**535**) was isolated. This compound showed cytotoxicity against HCT-116, A2780, K562, and A2780CisR cell lines, with IC_50_ values ranging from 27.3 to 67.8 μM, compared to 0.8 to 33.4 μM for cisplatin [280]. From the marine-sponge-derived fungus *Aspergillus versicolor* SCSIO 41016, a new diketopiperazine alkaloid (**536**) exhibited weak cytotoxic activities against ACHN, OS-RC-2, and 786-O cells, with IC_50_ values ranging from 27.0 to 47.1 μM [281]. A deep-sea-derived fungus, *Aspergillus sydowii* MCCC 3A00324, produced a novel acremolin type alkaloid named acremolin D (**537**), exhibiting inhibitory effects against the proliferation of Hela-S3 and K562 cell lines, with an inhibition rate of 30.6% and 25.1% at the concentration of 20 μM, respectively [282]. Additionally, a pentacyclic alkaloid named citrinadin C (**538**) was isolated from *Penicillium citrinum*, showing cytotoxic activities against the human liver cancer cell line MHCC97H, with an IC_50_ value of 16.7 μM [283]. *Aspergillus* sp. was found to produce asperphenins A (**539**) and B (**540**), demonstrating significant antiproliferative activity against various human cancer cell lines, including RKO colorectal carcinoma cells. The IC_50_ values for these compounds ranged from 0.8 to 9.7 μM, which was comparable to the positive control etoposide [284].

#### 2.4.2. Hybrids of Polyketides and Terpenoids (or Steroids or Isoprenyls)

**Figure 22:**
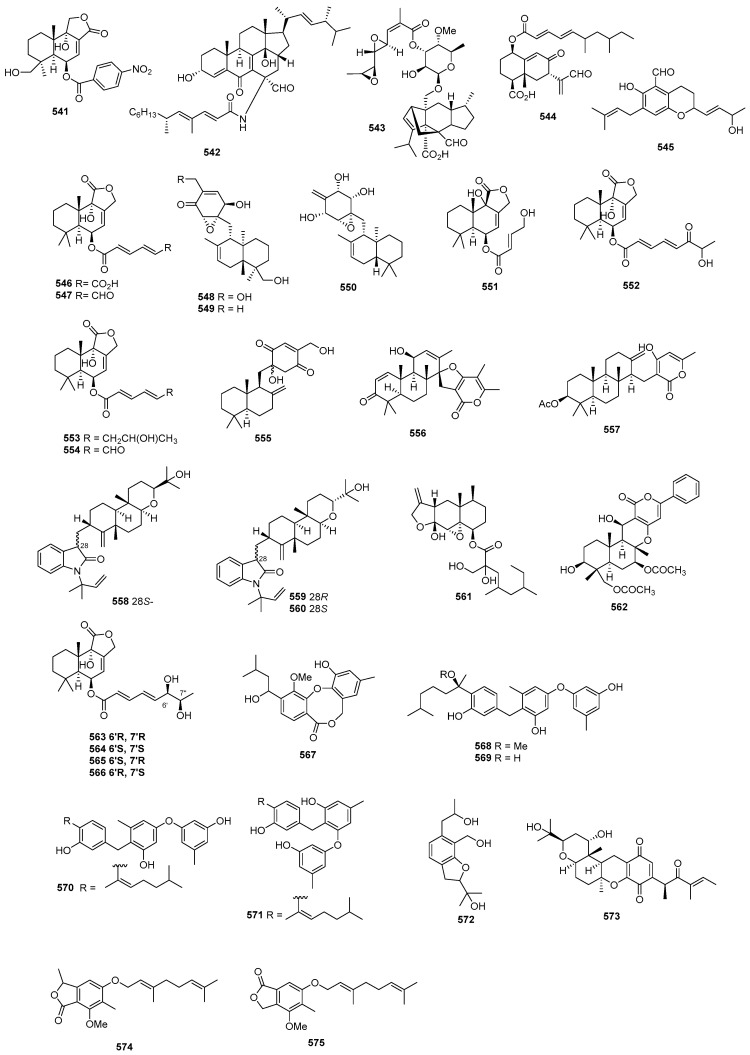
Structures of compounds **541**–**575**.

*Aspergillus versicolor* CNC 327, isolated from the surface of the Caribbean green alga *Penicillus capitatus*, produced a novel sesquiterpenoid nitrobenzoyl ester (**541**) (Figure 22). This compound exhibited potent cytotoxic effects against HCT-116, HCC-2998, SNB-75, and BT-549, with LC_50_ values ranging from 0.27 to 0.53 μg/mL. Additionally, it demonstrated selective cytotoxicity against CAK-1, 786-0, TK-10, ACHN, and UO-31, with LC_50_ values ranging from 0.47 to 0.57 μg/mL [285]. *Gymnacella dankaliensis*, a fungus derived from a Halichondria sponge, produced a novel compound called gymnasterone A (**542**). This compound exhibited inhibitory activity against P388, with an ED_50_ value of 10.1 μg/mL [252]. Another fungus, *Hypoxylon croceum*, yielded a sordarin derivative named hypoxysordarin (**543**), which displayed cytotoxicity against HL-60, with an IC_50_ value of 50 μg/mL [286]. A novel eremophilane sesquiterpene, 07H239-A (**544**), was isolated from *Xylariaceous* LL-07H239 and exhibited selective cytotoxic activity against CCRFCEM, with an IC_50_ value of 0.9 µg/mL [287]. *Chaetomium globosum*, isolated from the inner tissue of the marine red alga *Polysiphonia urceolata*, produced chaetopyranin (**545**), which displayed weak cytotoxicity against HMEC, SMMC-7721, and A549 cell lines, with IC_50_ values of 15.4, 28.5, and 39.1 µg/mL [288]. Two newly identified drimane sesquiterpenoids (**546**–**547**) were obtained from the fungus *Aspergillus ustus* 8009, isolated from the marine sponge *Suberites domuncula*. Compound **546** demonstrated cytotoxic activity against the L5178Y cell line, with an EC_50_ value of 5.3 µg/mL. On the other hand, compound **547** exhibited cytotoxic effects against L5178Y, PC12, and HeLa cell lines, with EC_50_ values of 0.6, 7.2, and 5.9 µg/mL, respectively [289]. Epoxyphomalins A (**548**) and B (**549**), characterized by unusual structural features, were isolated from *Phoma* sp. These compounds demonstrated mean IC_50_ values of 0.11 and 1.25 µg/mL against 36 human tumor cell lines [290]. Epoxyphomalin D (**550**), produced by *Paraconiothyrium* sp. 193H12, displayed cytotoxic activity against prostate PC3M and bladder BXF 1218L, with IC_50_ values of 0.72 and 1.43 µM, respectively [291]. A new compound, (*E*)-6-(4′-hydroxy-20-butenoyl)-strobilactone A (**551**), was isolated from *Aspergillus insuetus* (OY-207), which was purified from the Mediterranean sponge *Psammocinia* sp. This compound exhibited mild cytotoxicity against MOLT-4 human leukemia cells, with an inhibition rate (IR%) of 55% at 50 mg/mL [249]. Additionally, a novel drimane sesquiterpene (**552**) was isolated from *Aspergillus ustus*, displaying antitumor activity against P388, with an IC_50_ of 8.7 μM [292]. *Aspergillus ustus* 094102 yielded ustusolates C (**553**) and E (**554**). Among these compounds, compound **554** demonstrated cytotoxicity against HL-60, with an IC_50_ value of 9.0 μM, while compound **553** showed moderate cytotoxicity against A549, with an IC_50_ value of 10.5 μM [293].

Penicilliumin A (**555**) was extracted from *Penicillium* sp. F00120, isolated from a deep-sea-sediment sample, and demonstrated moderate cytotoxic activity against B16, A375, and Hela cell lines, with GI_50_ values of 27.37, 22.88, and 44.05 µg/mL, respectively [294]. Another newly discovered compound, brevione I (**556**), was obtained from *Penicillium* sp. C9408-3 in deep-sea sediment, exhibiting cytotoxicity against MCF-7, with an IC_50_ value of 7.44 µM [295]. Aszonapyrone A (**557**) and aszonapyrone B (**558**), isolated from the coral-derived fungus *Neosartorya laciniosa* KUFC 7896, showed significant growth inhibition. Aszonapyrone A (**557**) displayed lower GI_50_ values (13.6, 11.6, and 10.2 µM) against MCF-7, NCI-H460, and A375-C5 cell lines compared to sartorypyrone B (**558**) [296]. Anthcolorins B–D (**558**–**560**), unique tetrahydropyrane diterpene metabolites with oxoindoline at C-3, were derived from *Aspergillus versicolor* OUPS-N136, originally purified from the sea urchin *Anthocidaris crassispina*. These compounds exhibited cytotoxic activity against P388, with IC_50_ values ranging from 2.2 to 8.5 μM, which is comparable to 5-fluorouracil (the positive control) with an IC_50_ value of 1.2 μM [297]. Cryptosphaerolide (**561**), an ester-substituted sesquiterpenoid from *Cryptosphaeria* sp. CNL-523, displayed cytotoxicity against HCT-116, with an IC_50_ value of 4.5 µM [298]. *Penicillium concentricum* ZLQ-69 produced phenylpyropene E (**562**), demonstrating cytotoxicity against the MGC-803 cell line, with an IC_50_ value of 19.1 µM [299]. Asperienes A–D (**563**–**566**), four C-6′/C-7′ epimeric drimane sesquiterpene esters, were isolated from *Aspergillus flavus* CF13-11. These compounds showed potent bioactivities against HeLa, MCF-7, MGC-803, and A549, with IC_50_ values ranging from 1.4 to 8.3 µM. However, they also exhibited cytotoxicity against GES-1 cells, with IC_50_ values of 78, 6.2, 4.9, and 83 µM [300]. *Paecilomyces* sp., a mangrove fungus from the Taiwan Strait, yielded paeciloxocin A (**567**), exhibiting strong cytotoxicity against HepG2, with an IC_50_ of 1 μg/mL [301]. *Penicillium expansum* 091006, obtained from the mangrove plant *Excoecaria agallocha*, produced two new polyphenols, expansols A (**568**) and B (**569**). Expansol A (**568**) displayed cytotoxicity against HL-60, with an IC_50_ of 15.7 µM, while expansol B (**569**) exhibited cytotoxicity against A549 and HL-60 cells, with IC_50_ values of 1.9 and 5.4 µM, respectively [302]. Meanwhile, the fungi produced expansols C (**570**) and E (**571**), showing weak cytotoxicity against HL-60 cell lines, with IC_50_ values of 18.2 and 20.8 µM, respectively [303]. *Aspergillus ustus* 094102 yielded ustusorane E (**572**), which exhibited cytotoxicity against HL-60, with an IC_50_ of 0.13 μM [293]. *Nigrospora* sp. MA75, an endophytic fungus derived from the marine semimangrove plant *Pongamia pinnata*, produced compound **573**, which demonstrated moderate activity against SMMC7721, MCF-7, and SW1990, with IC_50_ values of 7, 4, and 5 μg/mL, whereas fluorouracil (the positive control) demonstrated IC_50_ values of 2, 4, and 16 μg/mL [304]. *Stachylidium* sp. 220, isolated from the sponge *Callyspongia* sp. cf. *C. flammea*, yielded two new phthalide derivatives, marilones A (**574**) and C (**575**), exhibiting weak antiproliferative activity, with average GI_50_ values of 36.7 and 26.6 µM [305].

**Figure 23:**
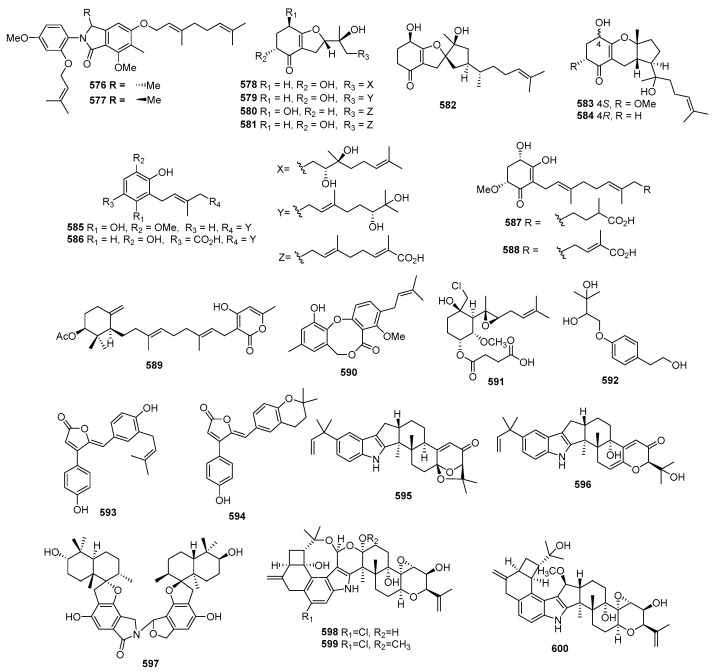
Structures of compounds **576**–**600**.

Marilines A_1_ (**576**) (Figure 23) and A_2_ (**577**) were also produced, with **576** showing cytotoxicity against five cancer cell lines (with a mean GI_50_ of 24.4 µM) and **577** exhibiting cytotoxicity against 19 cancer cell lines (with a mean GI_50_ of 11.02 µM) [306]. *Alternaria* sp. JJY-32 produced bicycloalternarenes A–D (**578**–**581**), tricycloalternarenes A–C (**582**–**584**), and monocycloalternarenes A–D (**585**–**588**), all inhibiting RAW264.7 cells, with IC_50_ values ranging from 39 to 85 µM [307]. *Neosartorya fischeri* KUFC 6344 yielded a new meroditerpene (**589**) active against NCI-H460, MCF-7, and A375-C5, with IC_50_ values of 37.3, 46.3, and 21.5 μM [296]. Prenpenicillide (**590**), a novel penicillide derivative from *Penicillium* sp. ZLN29, showed weak cytotoxicity against HepG2 cells (with an IC_50_ value of 9.9 µM) [308]. Ligerin (**591**), a novel chlorinated sesquiterpenoid analogue of fumagillin from *Penicillium canescentia* MMS35, exhibited strong inhibitory activity against POS1, with an IC_50_ value of 117 nM [309]. *Penicillium* sp. FJ-1 produced a new compound **592** with cytotoxicity against Tca8113 and MG-63 cells (with IC_50_ values of 26 and 35 µM, respectively) [220]. *Aspergillus terreus* OUCMDZ-1925 yielded rubrolides R (**593**) and S (**594**), both displaying cytotoxic activity against K562, with IC_50_ values of 12.8 and 10.9 μM, while the IC_50_ value of adriamycin (the positive control) against K562 was 0.64 μM [310]. Two new indole-diterpenoids (**595**–**596**) from *Aspergillus flavus* OUCMDZ-2205 arrested the A549 cell cycle in the S phase at a concentration of 10 μM. Additionally, compounds **595**–**596** exhibited weak cytotoxic activity against MCF-7 and A549, with IC_50_ values of 18–30 μM [311]. *Stachybotrys* sp. MF347 produced compound **597**, a spirocyclic drimane with activity on NIH-3T3 and HepG2 cells (with IC_50_ values of 13.0 and 14.3 µM) [312]. *Mucor irregularis* QEN-189, originally isolated from the marine mangrove *plant Rhizophora stylosa*, yielded rhizovarins A, B, and E (**598**–**600**), which were cytotoxic against the A-549 cell line, with IC_50_ values of 11.5, 6.3, and 9.2 µM. Compounds **598** and **599** also showed cytotoxicity against the HL-60 cell line, with IC_50_ values of 9.6 and 5.0 µM, respectively [313].

#### 2.4.3. Hybrids of Peptides and Terpenoids (or Isoprenyls)

**Figure 24:**
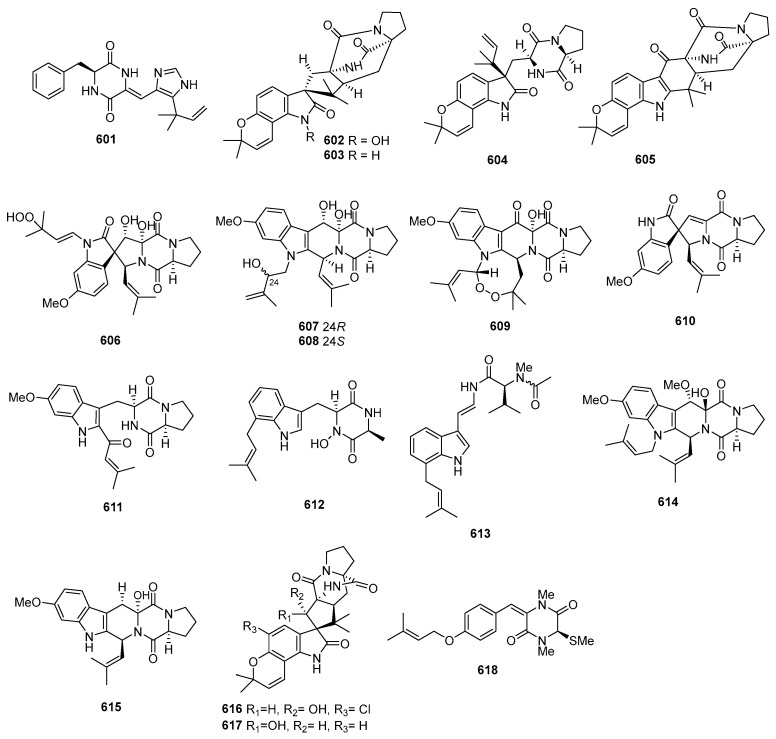
Structures of compounds **601**–**618**.

(–)-Phenylahistin (**601**) (Figure 24) was obtained from *Aspergillus ustus* and demonstrated potent cytotoxicity against various cell lines, including A431, A549, Hela, K562, MCF7, WiDr, and P388, with IC_50_ values ranging from 0.18 to 0.33 μM [314]. Notoamides A–C (**602**–**604**), isolated from *Aspergillus* sp., exhibited cytotoxicity against Hela and L1210, with IC_50_ values ranging from 22 to 52 μg/mL [315]. Notoamide I (**605**), also produced by the fungi, displayed weak cytotoxicity against HeLa, with an IC_50_ value of 21 µg/mL [316]. Spirotryprostatin E (**606**), along with two derivatives of fumitremorgin B (**607**–**608**) and 13-oxoverruculogen (**609**), were isolated from *Aspergillus fumigatus*. Compound **606** showed cytotoxicity against A549, MOLT-4, and HL-60, with IC_50_ values of 3.1, 3.1, and 2.3 μM, while compound **607** displayed cytotoxicity against BEL-7402, A549, MOLT-4, and HL-60, with IC_50_ values of 7.0, 11.0, 11.0, and 3.4 μM. Compounds **608** and **609** exhibited cytotoxicity against HL-60, with IC_50_ values of 5.4 and 1.9 μM, whereas VP16 (the positive control) inhibited BEL-7402, A549, MOLT-4, and HL-60, with IC_50_ values of 0.003–1.400 μM [317]. Three new diketopiperazine alkaloids, 6-methoxyspirotryprostatin B (**610**), 18-oxotryprostatin A (**611**), and 14-hydroxyterezine D (**612**), were isolated from *Aspergillus sydowi* PFW1-13. These compounds displayed weak cytotoxicity against A-549 cells, with IC_50_ values of 8.29, 1.28, and 7.31 µM, respectively. Compound **610** was slightly cytotoxic against HL-60, with an IC_50_ value of 9.71 µM [318]. Indole-3-ethenamide (**613**), isolated from halotolerant *Aspergillus sclerotiorum* PT06-1, exhibited cytotoxicity against HL-60 and A549, with IC_50_ values of 27 and 3.0 μM [319]. *Aspergillus fumigatus* YK-7 produced two new diketopiperazines, prenylcyclotryprostatin B (**614**) and 9-hydroxyfumitremorgin C (**615**), which showed cytotoxicity against U937, with IC_50_ values of 25.3 and 18.2 μM [320]. Two new prenylated indole alkaloids, 5-chlorosclerotiamide (**616**) and 10-epi-sclerotiamide (**617**), were isolated from *Aspergillus westerdijkiae* DFFSCS013. These compounds exhibited cytotoxicity against K562, with IC_50_ values of 44 and 53 µM [321]. A new diketopiperazine (**618**) from the Antarctic marine-derived fungus *Penicillium crustosum* HDN153086 displayed cytotoxicity against K562 cells, with an IC_50_ value of 12.7 μM [322].

#### 2.4.4. Other Hybrids

**Figure 25:**
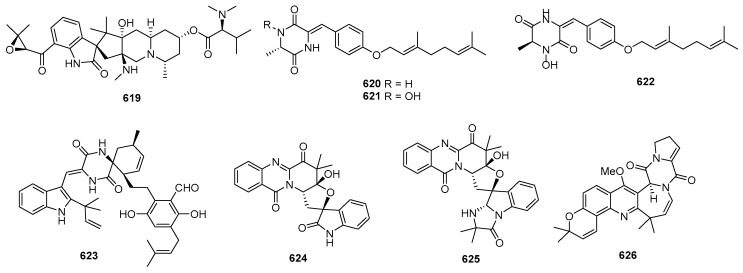
Structures of compounds **619**–**626**.

Citrinadin A (**619**) (Figure 25) is a recently discovered pentacyclic alkaloid isolated from *Penicillium citrinum*, derived from a marine red alga. In preliminary tests, citrinadin A (**619**) demonstrated moderate cytotoxic effects on murine leukemia L1210 cells and KB cells, with IC_50_ values of 6.2 and 10 µg/mL, respectively [323,324]. PJ147 (**620**), a novel diketopiperazine, was identified in *Gliocladium* sp. YUP08, originally isolated from sea mud in Rushan. PJ147 exhibited cytotoxicity against U937, HL-60, and T47D cells, with IC_50_ values of 0.79, 2.02, and 30.51 µM, respectively [325,326]. Additionally, two new piperazine-2,5-dione derivatives, gliocladrides A (**621**) and B (**622**), from the same fungi, displayed cytotoxic effects on U937, HL-60, and T47D cells, with IC_50_ values ranging from 11.60 to 52.83 µM, while vincristin (the positive control) inhibited these cell lines, with IC_50_ values of 1.67–12.57 μM [326]. Dihydrocryptoechinulin D (**623**) was isolated from *Aspergillus effuses* H1-1, sourced from mangrove rhizosphere soil. This compound exhibited potent activity against HL-60 and P388 cells, with IC_50_ values of 4.80 and 1.83 µM [327]. Tryptoquivalines T (**624**) and U (**625**), two novel alkaloids isolated from *Neosartorya fischeri*, demonstrated activity against HL-60 cells, with IC_50_ values of 82.3 and 90.0 µM [328]. Versicamide H (**626**), isolated from *Aspergillus versicolor* HDN08-60, displayed moderate cytotoxicity against HCT-116, Hela, K562, and HL-60 cells, with IC_50_ values of 17.7, 19.4, 22.4, and 8.7 μM, respectively [329].

### 2.5. Others

**Figure 26:**
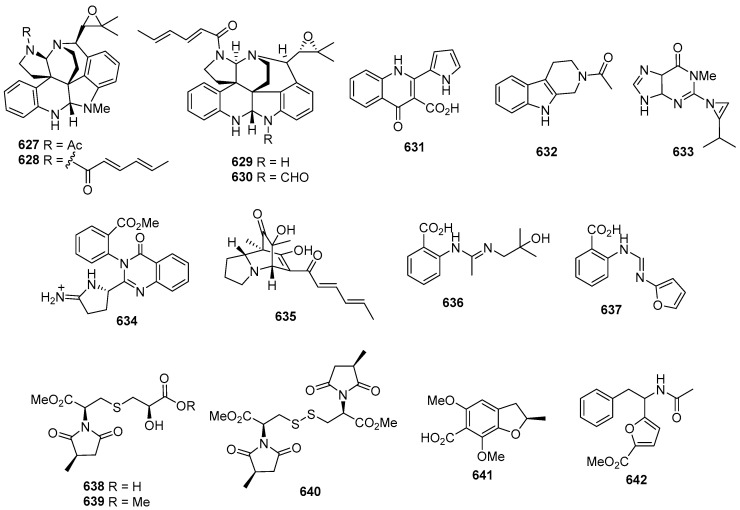
Structures of compounds **627**–**642**.

*Penicillium* sp. strain, isolated from the marine alga *Enteromorpha intestinalis*, produced communesins A (**627**) (Figure 26) and B (**628**), with cytotoxic activity against P-388 lymphocytic leukemia cells, exhibiting ED_50_ values of 3.5 and 0.45 µg/mL, respectively [330]. Another *Penicillium* sp. strain, originally obtained from the Mediterranean sponge *Axinella verrucosa*, yielded communesins C (**629**) and D (**630**). These compounds demonstrated cytotoxicity against U-937, THP-1, NAMALWA, MOLT-3, and SUP-B15 cells, with ED_50_ values ranging from 8.2 to 16.2 μg/mL [331]. From a mangrove endophytic fungus *Penicillium* sp., a novel pyrrolyl 4-quinolinone alkaloid named penicinoline (**631**) was isolated. Penicinoline exhibited cytotoxicity against 95-D and HepG2 cell lines, with IC_50_ values of 0.57 and 6.5 µg/mL [332]. An unusual alkaloid (**632**), isolated from *Fusarium incarnatum* (HKI0504) purified from the mangrove plant *Aegiceras corniculatum*, showed weak antiproliferative and cytotoxic activities against HUVEC and K-562, with GI_50_ values of 41.1 and 33.3 µM. Additionally, compound **632** displayed cytotoxic activity against HeLa cells, with a CC_50_ of 23.8 µM [125]. *Acremonium strictum* yielded acremolin (**633**), a novel modified base, demonstrating cytotoxicity against A549, with an IC_50_ of 45.9 µg/mL (doxorubicin exhibited an IC50 of 1.83 lg/mL as a positive control) [333]. *Penicillium aurantiogriseum* produced auranomide B (**634**), which exhibited cytotoxic activity against HEPG2 cells, with an IC_50_ of 0.097 µM [334]. From the deep-sea-derived *Penicillium* sp. F23-2, a new nitrogen-containing sorbicillinoid named sorbicillamine A (**635**) was isolated. These compounds displayed cytotoxic activity against HeLa, BEL-7402, HEK-293, HCT-116, and P388 cell lines, with IC_50_ values exceeding 10 µM [159]. Penipacids A (**636**) and E (**637**), two new anthranilic acid derivatives from *Penicillium paneum* SD-44, showed inhibitory activity against the human colon cancer RKO cell line, with IC_50_ values of 8.4 and 9.7 µM [335]. *Aspergillus violaceus* WZXY-m64-17 yielded three new methylsuccinimide-based sulfur-bearing compounds named violaceimides A, B, and E (**638**–**640**). Among these, compounds **638** and **639** displayed cytotoxicity with IC_50_ values of 5.3, 1.8 μM against U937, and 1.5 and 2.51 μM against HCT-8, while **640** was active on U937, with an IC_50_ value of 16.6 μM [336]. *Aspergillus terreus* [CFCC 81836] produced asperterreusine A (**641**), exhibiting cytotoxicity against HL-60 and SW-480 cell lines, with IC_50_ values of 15.3 and 25.7 μM [337]. Additionally, a new ester furan derivative (**642**) isolated from *Aspergillus niger* BRF-074 demonstrated activity against the HCT-116 cell line, with an IC_50_ value of 2.9 μg/mL [338].

## 3. Conclusions and Perspectives

**Figure 27:**
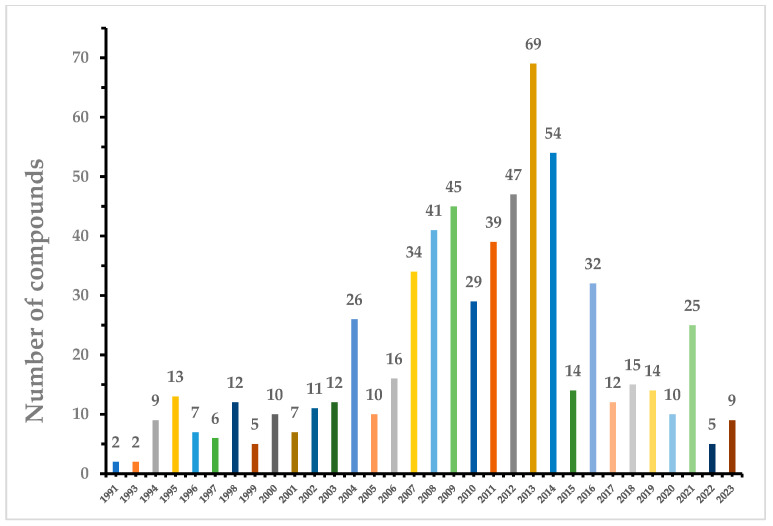
Numbers of antitumor compounds isolated from marine fungi each year (1991–2023).

The ocean, serving as a rich habitat for various microorganisms, presents significant untapped potential. Fungi inhabiting marine environments have proven to be prolific producers of secondary metabolites, yielding an abundance of novel compounds with exceptional cytotoxic properties. From 1991 to August 2023, a total of 642 previously undiscovered cytotoxic compounds have been isolated and characterized from marine fungi. While our efforts have been exhaustive in documenting these newfound cytotoxic agents, it is possible that some compounds have eluded inclusion in our compilation. This review, encapsulated in Table S1, provides a comprehensive overview of these novel natural products, encompassing details such as their chemical structures, originating strains, the sources of these strains, and their respective cytotoxic activities. The data gleaned from the summary of cytotoxic compounds isolated from marine-derived fungi spanning 33 years (1991–2023) indicates a notable trend. The majority of these compounds (546 in total) emerged between 2004 and 2023, as illustrated in Figure 27. It is evident that the quantity of reported cytotoxic compounds has steadily increased since 1993, reaching its peak in 2013 with a record high of 69 new compounds. Subsequently, there has been a declining trend in the number of reported cytotoxic compounds. Remarkably, from 1995 to 2021, each year witnessed the discovery of ten or more new cytotoxic compounds, with the exceptions being 1996, 1997, 1999, and 2001.

**Figure 28:**
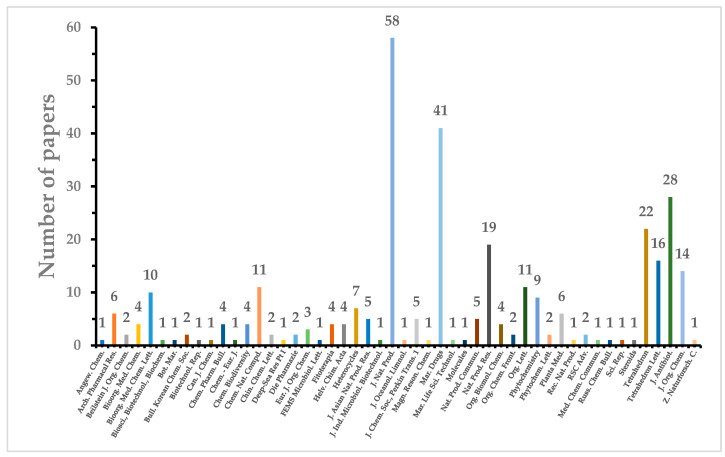
Journals that reported antitumor compounds and numbers of papers published (1991–2023).

The articles reporting these 642 compounds have been published in 50 different journals. Most of the articles reporting these compounds in the period of time (1991–2023) were published in *J. Nat. Prod.* (58), *Mar. Drugs* (41), *J. Antibiot.* (28), *Tetrahedron* (22), *Nat. Prod. Res.* (19), *Tetrahedron Lett.* (16), *J. Org. Chem.* (14), and *Org. Lett.* (11) (Figure 28). The main journals that reported the cytotoxic compounds from marine fungi were *J. Nat. Prod.* (17.4%), *Mar. Drugs* (12.3%), *J. Antibiot.* (8.4%), *Tetrahedron* (6.6%), *Nat. Prod. Res.* (5.7%), *Tetrahedron Lett.* (4.8%), *J. Org. Chem.* (4.2%), and *Org. Lett.* (3.3%) (Figure S1). In particular, the number of articles of these compounds published in *Phytochemistry* was nine, which were second only to the major journals mentioned above.

**Figure 29:**
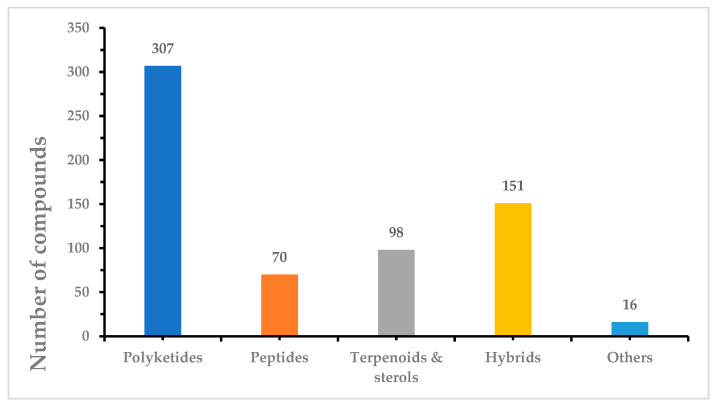
Structural classes of antitumor compounds from marine fungi (1991–2023).

Cytotoxic compounds, based on their structural characteristics, fall into five primary categories: polyketides, peptides, terpenoids and sterols, hybrids, and other miscellaneous compounds. These compounds display a wide array of chemical structures, with polyketides comprising the majority, totaling 307 compounds and accounting for 47.8% of the newly discovered antitumor agents (Figure 29). Among these polyketides, a significant proportion can be further categorized into macrolides, lactones, pyrones, and lactams (105 compounds), as well as chromones, xanthones, coumarins, benzoquinones, naphthoquinones, anthraquinones, and other aromatic compounds (121 compounds), collectively representing 35.2% of the total 642 cytotoxic compounds. Notably, the distribution of these compounds among marine-derived fungi varies. Specifically, the number of such compounds isolated from *Aspergillus* sp., *Penicillium* sp., and other fungal sources were 148, 140, and 354 compounds, respectively (Table S1). *Aspergillus* sp. emerged as the primary source of antitumor compounds, with *Penicillium* sp. following closely behind. These findings indicate that *Aspergillus* sp. and *Penicillium* sp. are significant producers of secondary metabolites in marine fungi, yielding a diverse range of promising compounds with potential biological activities.

Among the 642 compounds that have been documented, the majority have undergone testing for their cytotoxic activities, revealing predominantly moderate results. However, a subset of approximately twenty-three compounds within this dataset stands out due to their notably potent cytotoxic activity, exhibiting IC_50_ values at the nanomolar (nM) or nanogram per milliliter (ng/mL) scale. Examples of such compounds include **23** [13], **65** [41], **309**–**315** [183,184], **318**–**320** [186], **323**–**324** [187], **326** [188], **341** [194], **358** [203], **361** [206], **383** [220], **493**–**495** [183], and **591** [309]. It is noteworthy that most of these 642 compounds are constructed upon known structural frameworks or represent analogues of previously reported structures. Over the past decade, there has been a declining trend in the proportion of compounds with unique structural scaffolds derived from marine fungi. Nevertheless, the exploration and cultivation of uncharted and atypical microbial sources, including microorganisms residing in extreme environments, hold the potential to guide the discovery of novel compounds characterized by distinctive structures and exceptional biological activities. Recent years have witnessed a surge of interest among researchers in the realm of microbial biosynthesis, with the expectation of unearthing compounds featuring novel structures and unique properties through biological means. This pursuit involves the application of an increasing array of bioinformatics tools to identify potential biosynthetic gene clusters responsible for the production of fungal natural products. A routine sequencing of the genomes of fungal strains has further propelled this endeavor. The development of high-yield, broadly applicable expression systems for the biosynthesis of small molecules, the construction of genetic tools designed to harness the latent biosynthetic capabilities of cultured marine fungi, and the activation of “dormant” biosynthetic pathways all stand as pivotal strategies for the discovery of small molecules originating from marine fungi. Research aimed at comprehending the genetic and biochemical mechanisms underlying the biosynthetic pathways of marine fungi will open promising avenues for the design and identification of compounds endowed with enhanced anti-cancer properties.

## Supplementary Materials

The following supporting information can be downloaded at: https://www.mdpi.com/article/10.3390/md22020070/s1, Figure S1. Percentages of antitumor compounds published in different journals (1991–2023). Table S1. Cytotoxic compounds isolated from marine fungi (1991–2023).
