# Supplementary material for: Cytotoxic Compounds from Marine Fungi: Sources, Structures, and Bioactivity
Source: Mar Drugs. 2024 Jan 28;22(2):70. doi: 10.3390/md22020070 (PMC10890532; doi:10.3390/md22020070)
Supplement: Supplementary file 1 [file marinedrugs-22-00070-s001.zip › marinedrugs-2805340-supplementary.pdf]

Supplementary Material

Cytotoxic Compounds from Marine Fungi: Sources, Structures, and Bioactivity

Yukang Gao [1], Jianjian Wang [1], Pornphimon Meesakul [2], Jiamin Zhou [1], Jinyan Liu [1], Shuo Liu [1], Cong Wang [1,*] and Shugeng Cao [2,*]

[1] Key Laboratory of Chemistry and Engineering of Forest Products, State Ethnic Affairs Commission, Guangxi Key Laboratory of Chemistry and Engineering of Forest Products, Key Laboratory of Universities in Guangxi for Excavation and Development of Ancient Ethnomedicinal Recipes, Guangxi Collaborative Innovation Center for Chemistry and Engineering of Forest Products, Guangxi Minzu University, Nanning 530006, China; 18262343358@163.com (Y.G.); wangjianjian0114@163.com (J.W.); m14795747203@163.com (J.Z.); cheese77877@163.com (J.L.); 18231488462@163.com (S.L.)

[2] Department of Pharmaceutical Sciences, Daniel K. Inouye College of Pharmacy, University of Hawai'i at Hilo, Hilo, HI 96720, USA; pmeesak@hawaii.edu

* Correspondence: wangcong123206@163.com (C.W.); scao@hawaii.edu (S.C.)

Table of Contents

Figure S1. Percentages of antitumor compounds published in different journals (1991–2023). **1**
Table S1. Cytotoxic compounds isolated from marine fungi (1991–2023). **1**

Figure S1. Percentages of antitumor compounds published in different journals (1991–2023).

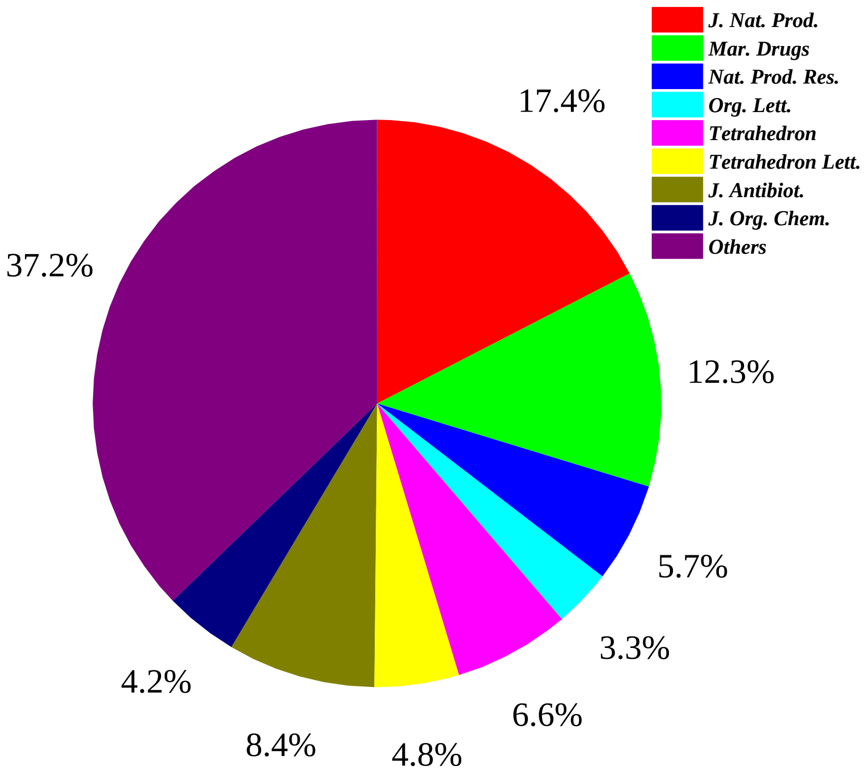

Table S1. Cytotoxic compounds isolated from marine fungi (1991–2023).

Compound	Producing strain	Strain source	Architectural feature	Ref.
**2.1. Polyketides**				
**2.1.1. Macrolides, lactones, pyrones, and lactams**				
**1**	*Hyphomycetes* sp.	Zoanthid *Zoanthus* sp., Amami Island, Kagoshima Prefecture, Japan	Azetinone (α,β-unsaturated-β-lactam)	[6]
**2**	*Periconia byssaides* OUPS-N133	Sea hare *Aplysia kurodai*, Osaka Bay of Japan	16-membered macrolide	[7]
**3–5**	*Aspergillus ostianus* 01F313	Unidentified marine sponge, Pohnpei, Federated States of Micronesia	14-membered macrolide	[8]
**6**	*Cladosporium* sp. F14	Seawater nearby the mangrove stand at Kei Ling Ha Lo Wai, Sai Kung, Hong Kong	nine-membered lactone	[9]
**7–8**	*Cladosporium* sp. L037	Brown alga, Seragaki Beach, Okinawa Island	12-membered macrolide	[10]

Compound	Producing strain	Strain source	Architectural feature	Ref.
**9–18**	*Dendrodochium* sp.	Sea cucumber *Holothuria nobilis* Selenka, South China Sea	12-membered macrolide	[11]
**19–22**	*Pestalotiopsis microspore.*	Fresh, healthy fruits of *Drepanocarpus lunatus*	14-membered macrolide	[12]
**23**	*Myrothecium roridum* 98F42	A submerged woody material, Palau	Macrocyclic trichothecene	[13]
**24**	*Myrothecium* sp. TUF 02F6	Marine sponge, Indonesia	Macrocyclic trichothecene	[14]
**25**	*Diaporthe* sp.	Submerged rotten leaves of *Kandelia candel*, Fujian Province of China	Isobenzofuranone or phthalide	[15]
**26**	*Penicillium* sp. BM923	Sea sediment sample, Miho, Sizuoka prefecture, Japan	Isobenzofuranone or phthalide	[16]
**27**	*Penicillium* sp. ZH58	Leaves of mangrove tree *Avicennia*, Dong Sai, Hainan of the South China Sea coast	Isobenzofuranone or phthalide	[17]
**28**	*Chrysosporium articulatum*	Unidentified dictyoceratid sponge, the coast of Gagu-do, Korea	Isobenzofuranone or phthalide	[18]
**29**	*Guignardia* sp. 4382	South-China Sea	Isobenzofuranone or phthalide	[19]
**30**	*Acremonium* sp. AWA16-1	Sea mud off Awajishima Island, Japan	γ-lactone-δ-lactam rings	[20]
**31–32**	*Phialocephala* sp. FL30r	Deep-sea sediment	Sorbicillinoid and benzofuranone	[21]
**33**	*Pseudallescheria boydii*	Inner tissue of sea star *Acanthaster planci*, Hainan Sanya National Coral Reef Reserve	Isobenzofuranone or phthalide	[22]
**34–40**	*Aspergillus* sp. 16-02-1	Deep-sea sediment sample, Lau Basin hydrothermal vent, southwest Pacific	C_9 polyketides	[23]
**41**	*Trichoderma citrinoviride*	Sediment samples, Min River estuary, China	Dioxolanone ester derivative	[24]
**42**	*Verruculina enalia* BCC 22226	Driftwood, Phetchaburi province, Thailand	Oxa-spiro derivative	[25]

Compound	Producing strain	Strain source	Architectural feature	Ref.
**43**	*Aigiaus parvus* sp. BCC 5311	Mangrove wood	14-membered resorcylic macrolide	[26]
**44–45**	Zh6-B1	Bark of *Sonneratia apetala*, Zhu Hai, Guangdong, China	10-membered resorcylic	[27]
**46–48**	*Penicillium sumatrense* MA-92	Rhizosphere of the mangrove plant *Lumnitzera racemose*, WenChang, Hainan Island, China	Sulfur-containing curvularin derivative	[28]
**49**	MF593	Green alga, Toyama Bay, Japan Sea	Ramulosin derivative	[29]
**50**	*Penicillium waksmanii* Zaleski OUPS-N133	Brown alga *Sargassum ringgoldianum*	Pyrenocine derivative	[30]
**51**	*Petriella* sp. TUBS 7961	Marine sponge *Suberitis domuncula*, Rovinj, Croatia	Pyrenocine derivative	[31]
**52**	*Penicillium chrysogenum* QEN-24S	Unidentified marine red algal species of the genus *Laurencia*	α-pyrone derivative	[32]
**53**	*Penicillum citreonigrum* XT20-134	Southeast Indian Ocean sediments at 2910 m	Pyrenocine derivative	[33]
**54**	*Aspergillus* sp. 16-02-1	Deep-sea sediment sample, Lau Basin hydrothermal vent, southwest Pacific	C_9 polyketides	[23]
**55**	*Penicillium citrinum*	Marine sediments, Langqi Island, Fujian, China	Alkaloid	[34]
**56**	Mixed broth of No. K38 and E33	South China sea coast	Diimide derivative	[35]
**57**	*Aspergillus flavus*	Marine algae *Enteromorpha tubulosa*, Putian Pinghai, China	Cyclopiazonic acid	[36]
**58–59**	*Monascus albidus* BB3	Fresh inner tissue of marine shellfish *Meretrix lusoria*, Hailing Island, Yangjiang, China	γ-lactam derivative	[37]
**60–61**	*Aspergillus oryzae*	Marine sediments, Langqi Island, Fujian, China	Tetracyclic oxindole alkaloids	[38]
**62**	*Trichoderma citrinoviride*	Sediment samples, Min River estuary, China	Succinimide derivative	[24]

Compound	Producing strain	Strain source	Architectural feature	Ref.
**63**	*Aspergillus versicolor* ZBY-3	Deep-sea water sample collected at a depth of 800 m in the southeast Pacific	γ-lactam derivative	[39]
**64**	*Aspergillus sydowi* D2-6	Marine sediments, Jiaozhou Bay, China	Hetero-spirocyclic γ-lactam	[40]
**65**	*Aspergillus fumigatus* OUPS-T106B-5	Marine fish *Mugil cephalus*	Hetero-spirocyclic γ-lactam	[41]
**66–67**	*Aspergillus fumigatus* OUPS-T106B-5	Marine fish *Mugil cephalus*	Hetero-spirocyclic γ-lactam	[42]
**68**	*Campylocarpon* sp. HDN13-307	Root of mangrove plant *S. caseolaris*	2-pyridone alkaloid derivative	[43]
**69–70**	*Aspergillus niger*	Mediterranean sponge *Axinella damicornis*	2-pyridone alkaloid derivative	[44]
**71**	*Aspergillus carbonarius* WZ-4-11	Marine sediment, Weizhou island, China	2-pyridone alkaloid derivative	[45]
**72**	*Aspergillus versicolor* Y31-2	Seawater samples, Indian Ocean	Phenylquinolinone	[46]
**73**	*Chaunopycnis* sp. CMB-MF028	Rock surfaces in the intertidal zone of Moora Park, Shorncliffe, Queensland	2-pyridone alkaloid derivative	[47]
**74–76**	*Chaetomium globosum* OUPS-T106B-6	Marine fish *Mugil cephalus*	Chloro-azaphilone derivative	[48]
**77–89**	*Chaetomium globosum* OUPS-T106B-6	Marine fish *Mugil cephalus*	Chloro-azaphilone derivative	[49]
**80–81**	*Chaetomium globosum* OUPS-T106B-6	Marine fish *Mugil cephalus*	Chloro-azaphilone derivative	[50]
**82**	*Penicillium chrysogenum*	Mediterranean sponge *Ircinia fasciculata*	Sorbicillin derivative	[51]
**83–86**	*Chaetomium globosum* OUPS-T106B-6	Marine fish *Mugil cephalus*	Chloro-azaphilone derivative	[52]
**87–88**	*Chaetomium globosum* OUPS-T106B-6	Marine fish *Mugil cephalus*	Azaphilone derivative	[53]
**87**	*Chaetomium globosum* HDN151398	Deep-sea sediment sample, South China Sea	Chloro-azaphilone derivative	[54]
**90–91**	*Phomopsis tersa* FS441	Sediment sample collected at the depth of 3000 m in the Indian Ocean	Chloro-azaphilone derivative	[55]

Compound	Producing strain	Strain source	Architectural feature	Ref.
**92–94**	*Chaetomium* sp. NA-S01-R1	Seawater sample at a depth of 4050 m, West Pacific Ocean	Chloro-azaphilone derivative	[56]
**95–96**	*Pyrenochaetopsis* sp. FVE-001	Brown alga *Fucus vesiculosus*, Kiel Fjord	Decalinoylspirotetramic acid derivative	[57]
**97**	*Mollisia* sp. SCSIO41409	Root bark of *Ardisia cornudentata* Mez	Chlorinated pyrrole-2,5-dione	[58]
**98**	*Talaromyces* sp. SCSIO 41050	Mangrove sediment sample, Zhanjiang, coastline of the northern part of Beibu Gulf, China	Maleic anhydride derivative	[59]
**99**	*Talaromyces* sp. HM-4-3-T3	Sea water, Dongshan Island, China	Benzoquinone	[60]
**100**	*Cladosporium halotolerans* FS702	Sediment sample collected at the depth of 2372 m in the South China Sea	Pyrone derivative	[61]
**101**	*Aspergillus aculeatinus* WHF0198	Sediments collected at the deep-sea of the South China Sea	Paraherquamide	[62]
**102**	*Alternaria* sp. LV52	Deep-sea cold seep sediment sample collected from the South China Sea	Polyketide	[63]
**103**	*Pestalotiopsis* sp. HQD-6	Leaf of Chinese mangrove plant *Rhizophora mucronate*	Polyketide	[64]
**104**	*Trichoderma* sp. 307	Stem bark of *Clerodendrum inerme*, Zhanjiang Mangrove National Nature Reserve in Guangdong Province, China	Depsidone	[65]
**105**	*Penicillium* sp. XL-01	Bohai Sea	Verrucosidin derivative	[66]
**2.1.2. Chromones, xanthones, coumarins, benzoquinons, naphthoquinones, anthraquinones and other aromatic compounds**				
**106–108**	*Aspergillus* sp. ZA-01	Sediment collected from the Bohai Sea of Huanghuagang, Hebei Province of China	Prenylxanthones	[67]
**109–111**	*Penicillium brocae* F97S76	Tissue homogenate of *Zyzzya* sp. sponge, Fiji	Polyketides	[68]

Compound	Producing strain	Strain source	Architectural feature	Ref.
**112**	*Phomopsis* sp. ZSU-H26	Stem of the mangrove tree *Excoecaria agallocha*, Dong Zai, Hainan, China	Naphtho-γ-pyrone	[69]
**113**	*Trichoderma* sp. AF007	Seastar *Acanthaster planci*, Hainan Sanya National Coral Reef Reserve, China	Sorbicillinoid analogue	[70]
**114**	*Penicillium oxalicum* SCSGAF 0023	South China Sea gorgonian *Muricella flexuosa*	Dihydrothiophene-condensed chromone	[71]
**115–116**	*Penicillium oxalicum* SCSGAF 0023	South China Sea gorgonian *Muricella flexuosa*	Dihydrothiophene-condensed chromone	[72]
**117**	*Penicillium purpurogenum* G59	Soil sample collected at the tideland of Bohai Bay, China	Chromone	[73]
**118**	*Penicillium purpurogenum* G59	Soil sample collected at the tideland of Bohai Bay, China	Cyclopentachromone sulfide	[74]
**119**	*Cladosporium halotolerans* GXIMD 02502	Coral *Porites lutea*, Weizhou Islands coral reef in Guangxi Zhuang autonomous region, China	Benzopyranone	[75]
**120**	*Pestalotiopsis* sp.	Leaves of the Chinese Mangrove plant *Rhizophora mucronate*	Chromone	[76]
**121–124**	*Rhytidhysteron rufulum*	Leaves of the Thai mangrove plant *Bruguiera gymnorrhiza*	Oxygenated chromones	[77]
**125–126**	*Penicillium purpurogenum* G59	Soil sample collected at the tideland of Bohai Bay, China	Chromones	[73]
**127–129**	*Tritirachium* sp. SpB081112MEf2	Marine sponge *Pseudoceratina purpurea*, offshore sites in Sakuraguchi, Ishigaki Island, Okinawa Prefecture, Japan	Xanthoquinodins	[78]
**130**	*Phomopsis* sp. (No. SK7RN3G1)	Mangrove sediment of Shankou, Hainan, China	Xanthone	[79]

Compound	Producing strain	Strain source	Architectural feature	Ref.
**131**	*Phomopsis* sp. ZH76	Stem of *Excoecaria agallocha* of the mangrove tree Euphorbiaceae, Dong Sai of the South China Sea coast	Xanthone *O*-glycoside	[80]
**132**	*Engyodontium album* DFFSCS021	Marine sediment sample, South China Sea	Chromone	[81]
**133–134**	*Aspergillus nomius* NC06	Marine sponge *Neopetrosia chaliniformis*, Mandeh Island, West Sumatra, Indonesia	Oxisterigmatocystin derivatives	[82]
**135**	*Aspergillus niger*	Mediterranean sponge *Axinella damicornis*	Polyketides	[44]
**136**	*Aspergillus flavus* 092008	Root of the mangrove plant *Hibiscus tiliaceus* (Malvaceae), Wenchang, Hainan province of China	Aflatoxin	[83]
**137**	*Penicillium* sp.	Deep water (-4380 ft) sedimentX	Pentaketide	[84]
**138**	No. ZSU-H16	Leaves of the mangrove tree *avicennia* from the South China Sea coast	Xanthone	[85]
**139**	*Fusarium* sp. ZZF41	Stem of the mangrove tree *Kandelia candel* (L.) Druce	Isoflavone	[86]
**140**	*Fusarium* spp. PSU-F135	Gorgonian sea fan (*Annella* sp.) collected near Koh Hin Ran Pet, Suratthani Province	Hydronaphthoquinone	[87]
**141**	*Nigrospora* sp. ZJ-2010006	Unidentified sea anemone (GX-WZ-20100026), Weizhou coral reef, South China Sea	Hydroanthraquinone analogue	[88]
**142**	*Acaromyces ingoldii* FS121	Marine sediment sample, South China Sea	Pyrandione analogue	[89]
**143**	*Penicillium* sp. F011	Korea	Polyaromatic metabolite	[90]
**144**	*Emericella variecolor*	Marine sponge Haliclona valliculata, collected in a depth of 20–30 m at Secca di Capo di Fonza, Italy	Anthraquinone evariquinone	[91]

Compound	Producing strain	Strain source	Architectural feature	Ref.
**145**	*Alternaria* sp. ZJ-2008003	*Sarcophyton* sp. soft coral, South China Sea	Anthranoid dimer	[92]
**146**	*Halorosellinia* sp. 1403	Mangrove	Anthracenedione derivative	[93]
**147–148**	*Alternaria* sp. ZJ9-6B	Mangrove *Aegiceras corniculatum*, South China Sea	Bianthraquinone derivative	[94]
**149**	*Aspergillus* sp. SCSIO F063	Marine sediment sample, South China Sea	Chlorinated anthraquinone	[95]
**150**	*Stemphylium globuliferum*	*Mentha pulegium* (Lamiaceae), Morocco	Anthracene derivative	[96]
**151–152**	*Stemphylium globuliferum*	*Juncus acutus* collected from the shore of the hypersaline lake El Hamra in Wadi el Natrun, Egypt	Tetrahydroanthraquinone derivatives	[97]
**153**	*Aspergillus glaucus*	Marine sediment, Fujian, China	Nnthraquinone derivative	[98]
**154**	*Emericella variecolor*	Sponge collected in Venezuelan waters of the Caribbean Sea	Polyketides	[99]
**155**	*Humicola grisea* Traaen	Drifting wood, southeastern lagoon of New Caledonia	Anthraquinone derivative	[100]
**156**	*Phialocephala* sp. FL30r	Deep-sea sediment	Polyketides	[21]
**157**	*Penicillium terrestre*	Marine sediments, Jiaozhou Bay, China	Monomeric derivative	[101]
**158**	*Trichoderma* sp. f-13	Marine sediment, Fujian, China	Polyketides	[102]
**159**	*Penicillium* sp. M207142	Sea sediment	Polyketides	[103]
**160**	*Aspergillus fischeri* FS452	Deep-sea sludge, Indian Ocean	Globoscin derivative	[104]
**161**	*Alternaria* sp. MCCC 3A00467	Sediment of Pacific Ocean at depth of 5295 m	Phomalone derivative	[105]

Compound	Producing strain	Strain source	Architectural feature	Ref.
**162**	*Lasiodiplodia* sp. 318#	*Excoecaria agallocha* of Mangrove National Nature Reserve in Gaoqiao, Zhanjiang city, Guangdong, China	Polyketides	[106]
**163**	*Aspergillus pseudodeflectus*	Seaweed, *Sargassum fusiform*, Miura Peninsula	Isochroman derivative	[107]
**164**	*Penicillium auratiogriseum*	Sponge *Mycale plumose*, Qingdao, China	Aromatic ester	[108]
**165**	*Penicillium janczewskii* H-TW5/869	Marine water sample	Quinolinone	[109]
**166**	*Acremonium* sp. AWA16-1	Sea mud, Awajishima Island, Japan	Dihydrobenzofuran derivative	[110]
**167**	*Aspergillus* sp. B-F-2	Marine sediments, Behai Bay, China	Diphenyl ether	[111]
**168**	*Aspergillus carbonarius* WZ-4-11	Marine sediment, Weizhou island, China	Polyketides	[45]
**169–176**	*Penicillium terrestre*	Marine sediments, Jiaozhou Bay, China	Gentisyl alcohol derivative	[101]
**177–178**	*Aspergillus versicolor* ZLN-60	Sediment of the Yellow Sea	Gentisyl alcohol derivatives	[112]
**179–181**	*Aspergillus taichungensis* ZHN-7-07	Root soil of the mangrove plant *Acrostichum aureum*	Polyketides	[113]
**182–183**	*Aspergillus aculeatus*		Polyketides	[114]
**184–185**	*Penicillium* sp. WC-29-5	Mangrove soil around the roots of *Aegiceras corniculatum*, Wenchang, Hainan Province of China	Polyketides	[115]
**186**	*Ascochyta* sp. NGB4	Floating scrap of festering rope collected at a fishing port in Nagasaki prefecture, Japan	Polyketides	[116]
**187–188**	*Rhytidhysteron* sp. AS21B	Leaves of *Azima sarmentosa*, collected from the mangrove forest in Samutsakhon province, Thailand	Polyketides	[117]

Compound	Producing strain	Strain source	Architectural feature	Ref.
**189**	*Phomopsis* sp. ZSU-H76	Stem of the mangrove tree E. agallocha, South China Sea	Polyketides	[118]
**190–191**	*Sporothrix* sp. (#4335)	Bark of the inshore mangrove tree *Kandelia candel*	Polyketides	[119]
**192–193**	*Penicillium citrinum*	marine sediments collected from Langqi Island, Fujian, China	Citrinin derivatives	[120]
**194–196**	*Penicillium commune* QSD-17	Marine sediment sample, southern China Sea	Azaphilone derivatives	[121]
**197**	*Penicillium paneum* SD-44	Marine sediment sample, South China Sea	Triazole carboxylic acid	[122]
**198**	*Aspergillus* sp.	Gorgonian *Dichotella gemmacea*	Benzylazaphilone derivative	[123]
**199–200**	*Paecilomyces variotii* EN-291	Red alga *Grateloupia turuturu*	Indole derivatives	[124]
**201**	*Fusarium incarnatum* (HKI0504)	Mangrove plant *Aegiceras corniculatum*	Alkaloid	[125]
**202–204**	*Penicillium raistrichii*	Saline soil sample, Bohai Bay in Zhanhua, China	Spiroketal	[126]
**205**	*Pestalotiopsis vaccinii*	A branch of *Kandelia candel* (L.) Druce (Rhizophoraceae), southern China	Aromatic amine	[127]
**206–207**	*Alternaria* sp. R6	Root of a marine semi-mangrove plant *Myoporum bontioides* A. Gray, Leizhou peninsula, Guangdong Province, China	Resveratrol derivatives	[128]
**208–209**	*Lasiodiplodia theobromae* ZJ-HQ1	Leaves of the marine mangrove *Acanthus ilicifolius*, Zhanjiang Mangrove Nature Reserve in Guangdong Province, China	Chlorinated preussomerins	[129]
**210**	*Penicillum citreonigrum* XT20-134	Southeast Indian Ocean sediments at 2910 m	Polyketides	[33]

Compound	Producing strain	Strain source	Architectural feature	Ref.
**211–212**	*Penicillium aculeatum*	Red alga *Laurencia obtusa*, Egyptian Red Sea	Sulfonyl metabolites	[130]
**213**	*Aspergillus candidus* OUCMDZ-1051	Unidentified sponge sample (No. XS-3), Xisha islands, South China Sea	Terphenyl	[131]
**214**	*Aspergillus micronesiensis*	An edible red seaweed *Kappaphycus alvarezii*	Dibenzospiroketal	[132]
**215**	*Eurotium* sp. SCSIO F452	Sea sediment sample, South China Sea	Salicylaldehyde derivative	[133]
**216**	*Alternaria* sp. LV52	Deep-sea cold seep sediment sample collected from the South China Sea	Polyketides	[63]
**217**	*Penicillium* sp. GXIMD 03101	Mangrove plant *Acanthus ilicifolius* L., South China Sea	Xanthene derivative	[134]
**218**	*Penicillium* sp. ZH16	Leaves of mangrove tree *avicennia* from the Dong Sai, Hainan of the South China Sea coast	Furanocoumarin	[135]
**219**	*Alternaria tenuis* Sg17-1	Marine alga, Zhoushan Island, China	Isocoumarin	[136]
**220–221**	*Chrysosporium articulatum*	Unidentified dictyoceratid sponge, the coast of Gagu-do, Korea	Isocoumarin	[18]
**222**	No. dz17	South China Sea	Isocoumarin	[137]
**223**	*Phomopsis sp.* Gx-4	Mangrove sediment, ZhuHai, Guangdong, China	Isocoumarin	[138]
**224**	*Penicillium* sp. 091402	Root of the mangrove *Bruguiera sexangula* Linn, Qinglan Port, Hainan, China	Isocoumarin	[139]
**225–226**	*Penicillium chrysogenum* SCSIO 41001	Deep sea sediment of the Indian Ocean	Isocoumarin	[140]
**2.1.3. Polyketides (Other cyclic polyketides)**				
**227–229**	*Penicillium* sp.	Marine alga *Emeromorpha intestinalis*	Cyclic polyketides	[141]
**230–232**	*Trichoderma harzianum* OUPS-N115	Marine sponge *Halichondria okadai*	Cyclic polyketides	[142]

Compound	Producing strain	Strain source	Architectural feature	Ref.
**233**	*Trichoderma* sp. GIBH-Mf082	Marine sediment, South China Sea	Cyclopentenone	[143]
**234–236**	*Gymnascella dankaliensis*	Sponge *Halichondria japonica*, Osaka Bay, Japan	Polyketides	[144]
**237–239**	*Gymnascella dankaliensis*	Sponge *Halichondria japonica*, Osaka Bay, Japan	Polyketides	[145,146]
**240–242**	*Gymnascella dankaliensis*	Sponge *Halichondria japonica*, Osaka Bay, Japan	Polyketides	[147]
**243–244**	*Gymnascella dankaliensis*	Sponge *Halichondria japonica*, Osaka Bay, Japan	Polyketides	[148]
**245–246**	*Gymnascella dankaliensis*	Sponge *Halichondria japonica*, Osaka Bay, Japan	Polyketides	[149]
**247–248**	*Periconia byssoides* OUPS-N133	Sea hare *Aplysia kurodai*	Polyketides	[150]
**249–253**	*Periconia byssoides* OUPS-N133	Sea hare *Aplysia kurodai*	Polyketides	[151]
**254**	*Aspergillus* sp.	Mussel *Mytilus edulis*, Toyama Bay	Polyketides	[152]
**255**	*Penicillium terrestre*	Marine sediment, Jiaozhou Bay, Qingdao	Polyketides	[153]
**256–259**	*Trichoderma reesei*	Marine mud in the tideland of Lianyungang, China	Polyketides	[154]
**260–263**	*Phialocephala* sp. FL30r	Underwater sample	Sorbicillin trimers	[155,156]
**264**	*Penicillium citrinum* SpI080624G1f01	Marine sponge *Demospongiae* collected offshore of Ishigaki island, Okinawa. Prefecture, Japan	Sorbicillinoid	[157]
**265–266**	*Penicillium terrestre*		Polyketides	[158]
**267**	*Trichoderma* sp. f-13	Marine sediment, Fujian, China	Polyketide	[102]
**268–270**	*Penicillium* sp. F23−2	Deep sea	Sorbicillinoids	[159]
**271–272**	*Penicillium terrestre*	Marine sediments, Jiaozhou Bay, China	Chlorinated sorbicillinoids	[160]
**273–274**	*Chaetomium globosum* OUPS-T106B-6	Marine fish, Mugil cephalus	Polyketides	[161]
**275–278**	*Chaetomium globosum* OUPS-T106B-6	Marine fish *Mugil cephalus*	Polyketides	[50]

Compound	Producing strain	Strain source	Architectural feature	Ref.
**279**	*Chaetomium globosum* OUPS-T106B-6	Marine fish, Mugil cephalus	Polyketides	[162]
**280**	*Penicillium* 303#	Sea water, Zhanjiang Mangrove National Nature Reserve in Guangdong Province, China	Polyketides	[163]
**281**	*Penicillium sclerotiorum* M-22	Rotted leaf sample collected on the west coast of Haikou, Hainan province, China	Azaphilonidal derivative	[164]
**282**	*Rhizopus* sp. 2-PDA-61	Bryozoan *Bugula* sp., Jiaozhou Bay, China	Pyran derivative	[165]
**283–284**	*Talaromyces* sp. ZH-154	Stem bark of *Kandelia candel*, Qi'ao Island of Zhuhai, China	Polyketides	[166]
**285**	*Penicillium raistrickii*	Saline soil collected along the coast of Bohai Bay in Zhanhua, Shandong Province of China	Polyketides	[167]
**286**	*Penicillium* sp. F23–2	Deep sea	Nitrogen-containing sorbicillinoid	[159]
**287**	*Aspergillus sulphureus* KMM 4640	Marine sediments	Decaline derivative	[168]
**288**	*Penicillium* sp. F11	Deep marine	Polyketides	[169]
**289**	*Penicillium purpurogenum* G59		Polyketides	[170]
**290**	*Isaria feline* KMM 4639	Marine sediments collected at a depth of 10 m, South China Sea, coast of Vietnam	Polyketides	[171]
**291**	*Penicillium citrinum* SCSIO41402	Marine alga *Coelarthrum* sp. collected in Yongxing Island, South China Sea	Sorbicillinoid	[172]
**292**	*Eurotium* sp. SCSIO F452	Sea sediment sample, South China Sea	Salicylaldehyde derivative	[133]
**2.1.4. Linear polyketides and others**				
**293–294**	*Gliocladium roseum* KF-1040	Seaweed sample collected at Yap Island	Linear polyketides	[173–175]

Compound	Producing strain	Strain source	Architectural feature	Ref.
**295–296**	*Aspergillus flavipes*	Sea anemone *Anthopleura xanthogrammica*	Linear polyketides	[176]
**297**	*Gliocladium* sp. L049	Sea grass *Syringodium isoetifolium*, Maeda Cape, Okinawa Island	Linear polyketides	[177]
**298**	*Aspergillus* sp. 16-02-1	Deep-sea sediment sample, Lau Basin hydrothermal vent, southwest Pacific	Linear polyketides	[23]
**299**	*Pestalotiopsis clavispora*	Mangrove plant *Rhizophora harrisonii*, Port Harcourt (Nigeria)	Linear polyketides	[178]
**300**	*Acremonium citrinum*. MMF4	Sediment at the root of mangrove plant *Kandelia obovata*, Yunxiao county, Zhangzhou City, Fujian Province, in China	Linear polyketides	[179]
**301**	*Fusarium graminearum* FM1010	Shallow-water volcanic rock, Richardson's Beach, Big Island, Hawaii	Linear polyketides	[180]
**302–307**	*Pestalotiopsis heterocornis* XWS03F09	Sponge *Phakellia fusca* collected from the Xisha Islands, close to Sansha City, Hainan Province, China	Polyketides	[181]
**2.2. Peptides**				
**2.2.1. Diketopiperazine**				
**308**	*Aspergillus niger*	Caribbean *Hyrtios* sponge	Diketopiperazines	[182]
**309–329**	*Leptosphaeria* sp. OUPS-4	Marine alga *Sargassum tortile*	Diketopiperazines	[183–188]
**330–333**	*Exserohilum rostratum* CNK-630	Cyanobacterial mat collected off the northwest corner of Lanai Island, Hawaii	Diketopiperazines	[189]
**334–336**	*Oidiodendron truncatum* GW3-13	Soil collected under lichens near the Great Wall station (Chinese Antarctic station)	Diketopiperazines	[190]
**337**	*Fusarium chlamydosporum* OUPS-N124	Marine alga *Carpopeltis affinis*	Diketopiperazines	[191]

Compound	Producing strain	Strain source	Architectural feature	Ref.
**338**	*Gliocladium roseum* OUPS-N132	The sea hare *Aplysia kurodai* collected in coast of Kata	Diketopiperazines	[192]
**339**	*Phoma* sp. OUCMDZ-1847	Mangrove plant *Kandelia candel* (Rhizophoraceae) Wenchan, Hainan Province, China	Diketopiperazines	[193]
**340–341**	*Penicillium brocae* MA-231	Mangrove	Diketopiperazines	[194]
**342**	*Penicillium purpurogenum* G59	Soil sample collected at the tideland of Bohai Bay	Diketopiperazines	[195]
**343–345**	*Aspergillus nidulans* SD-531	Deep-sea sediment collected in cold seep region (at a depth 1331 m) in the South China Sea	Diketopiperazines	[196]
**346–349**	*Aspergillus* sp. EGF 15-0-3	Soft coral	Diketopiperazines	[197]
**350–351**	*Penicillium* sp. F23-2	Underwater sample	Diketopiperazines	[198]
**352–353**	*Eurotium* sp. SCSIO F452	South China Sea sediment sample	Diketopiperazines	[199]
**2.2.2. Cyclicpetides**				
**354**	*Fusarium* sp. CNL-292	Seagrass *Halodule wrightii*	Cyclic pentadepsipeptide	[200]
**355**	*Fusarium* sp. CNL-619	Green alga *Avrainvillea* sp., southeast end of St. Thomas at Bovoni Cay, United States Virgin Islands	Cyclic depsipeptide	[201]
**356–357**	*Scytalidium* sp. CNC-310	Green alga *Halimeda* sp. collected from a patch reef at a depth of 15 m from the northern end of Long Island, the Bahamas	Cyclic heptapeptides	[202]
**358**	*Zygosporium masonii* CNK458	Marine cyanobacterium, island of Maui, Hawaii	Cyclic pentadepsipeptide	[203]
**359**	Strain No. 1962	Leaf of *Kandelia candel* from an estuarine mangrove, Hong Kong	Cyclic pentadepsipeptide	[204]
**360**	*Trichoderma reesei* YZ48-08		Cyclotetrapeptide	[205]

Compound	Producing strain	Strain source	Architectural feature	Ref.
**361**	*Clonostachys* sp. ESNA-A009	Unidentified marine sponge, Japan	Cyclodepsipeptide	[206]
**362–363**	*Spicellum roseum* 193H5	Sponge *Ectyplasia perox*	Cyclohexadepsipeptides	[207]
**364–365**	*Scopulariopsis brevicaulis*	Marine sponge *Tethya aurantium*, Limski Fjord, Croatia	Cyclodepsipeptides	[208]
**366**	*Aspergillus sclerotiorum* PT06-1	Putian SeaSalt Field, Fujian, China	Cyclic hexapeptide	[209]
**367**	*Bionectria ochroleuca*	Leaf tissues of the plant *Sonneratia caseolaris* (Sonneratiaceae) from Hainan Island (China)	Cyclicpetide	[210]
**368–369**	*Acremonium persicinum* SCSIO 115	Marine sediment sample, South China Sea	Cycloheptapeptides	[211]
**370**	*Phaeosphaeriopsis* sp. S296	Sediment of a mangrove plant *Bruguiera gymnorhiza*, Techeng Isle, Zhanjiang, Guangdong Province, China	Cyclodecadepsipeptide	[212]
**2.2.3. Linear peptides**				
**371–372**	*Acremonium* sp. 021172cKZ	Marine sponge *Teichaxinella* sp., Papua New Guinea	N-methylated linear octapeptides	[213]
**373**	*Aspergillus versicolor*	Marine sponge *Petrosia* sp.	Linear peptide	[214]
**374–377**	*Simplicillium obclavatum* EIODSF 020	Marine sediment sample, East Indian Ocean	Linear peptides	[215]
**2.3. Terpenoids and sterols**				
**2.3.1. Sesquiterpenoids**				
**378**	*Penicillium* sp. SS080624SCf1	Tunicate *D. molle*, Ishigaki Island, Okinawa Prefecture, Japan	Sesquiterpenoid	[216]
**379–380**	*Aspergillus* sp.	Marine sponge *Xestospongia testudinaria*, South China Sea	Sesquiterpenoid dimers	[217]
**381**	*Chondrostereum* sp. nov. SF002	Soft coral *Sarcophyton tortuosum*, the South China Sea	Triquinane-type sesquiterpenoid	[218]

Compound	Producing strain	Strain source	Architectural feature	Ref.
**382**	*Chondrostereum* sp. nov. SF002	Soft coral *Sarcophyton tortuosum*, the South China Sea	Sesquiterpenoid	[219]
**383**	*Penicillium* sp. FJ-1	Marine plant of *Avicennia marina*, Fujian, China	Sesquiterpenoid	[220]
**384–385**	*Penicillium* sp. PR19 N-1	Marine sludge collected from Prydz Bay (-1000 m), Antarctica	Eremophilane-type sesquiterpenes	[221]
**386–387**	*Ascotricha* sp. ZJ-M-5	Mud sample collected on the coastal beach in Fenghua County, Zhejiang Province, China	Sesquiterpenoids	[222]
**388**	*Aspergillus flocculosus*	Sediment sample, Nha Trang Bay, South China Sea, Vietnam	Sesquiterpenoid	[223]
**389**	*Aspergillus ochraceus* Jcma1F17	Marine alga *Coelarthrum* sp., Paracel Islands, South China Sea	Nitrobenzoyl sesquiterpenoid	[224]
**390**	*Penicillium chrysogenum* LD-201810	Marine red alga *Grateloupia turuturu*, Qingdao, China	Sesquiterpenoid	[225]
**391–392**	*Aspergillus niger*	Marine sponge *Dysidea* sp., South China Sea	Sesquiterpenoids	[226]
**393–394**	*Aspergillus flavipes* 297	Seawater collected at coastal zone of Yantai, China	Sesquiterpenoids	[227]
**396**	Unidentified fungi	Marine sponge *Jaspis* aff. *Johnstoni*	Sesquiterpenoid	[228]
**397**	*Acremonium neocaledoniae*	Drifting wood, Gadgi Bay, New Caledonia	Sesquiterpenoid	[229]
**398–401**	*Talaromyces flavus*	Leaves of mangrove plant *Sonneratia apetala* collected on the coastal saltmarsh of the South China Sea	Sesquiterpenoids	[230]
**402**	*Penicillium* sp. PR19N-1	Marine sludge collected from Prydz Bay (−1000 m), Antarctica	Chloro-trinoreremophilane sesquiterpene	[231]
**2.3.2. Diterpenoids**				

Compound	Producing strain	Strain source	Architectural feature	Ref.
**403**	*Arthrinium* sp. 9287	Mediterranean sponge *Geodia cydonium*, Adriatic Ocean	Diterpenoids	[232]
**404–405**	*Epicoccum* sp. HS-1		Pimarane diterpenes	[233]
**406–409**	*Eutypella scoparia* FS26	Marine sediment sample collected at the depth of 139 m in the South China Sea	Oxygenated pimarane diterpenes	[234]
**410**	*Eutypella* sp. FS46	Marine sediment sample collected at the depth of 292 m in the South China Sea	Pimarane-type diterpene	[235]
**411**	*Penicillium brefeldianum* strain WZW-F-69	Soil near an abalone aquaculture base of Fujian province, China	Indole diterpenoid	[236]
**412–413**	*Aspergillus wentii* EN-48	Unidentified marine brown algal species of the genus *Sargassum*	Tetranorlabdane diterpenoids	[237]
**414**	*Aspergillus terreus* GX73B	Coastal salt marsh of the South China Sea, Guangxi Province	Diterpenoids	[238]
**415**	*Aspergillus wentii* SD-310	Deep sea sediment sample, South China Sea at a depth of 2038 m	Diterpenoids	[239]
**416–420**	*Aspergillus wentii* SD-310	Deep-sea sediment sample	Diterpenoids	[240]
**421–423**	*Penicillium* sp.	Deep water sediment sample, East Pacific	Breviane spiroditerpenoids	[241]
**424–429**	*Penicillium* sp. F23-2	Underwater sample	Diterpenoids	[198]
**430**	*Aspergillus candidus* HDN15-152	Sponge collected from Pulitzer Bay, Antarctica	Indole diterpenoids	[242]
**431–433**	*Acremonium striatisporum* KMM 4401	Holothurian *Eupentacta fraudatrix*	Diterpene glycosides	[243]
**434–437**	*Acremonium striatisporum* KMM 4401	Holothurian *Eupentacta fraudatrix*	Diterpene glycosides	[244]
**2.3.3. Sesterterpenoids**				
**438–439**	*Fusarium heterosporum* CNC-477	Driftwood collected from a mangrove habitat at Sweetings Cay, Bahamas	Sesterterpenes	[245]

Compound	Producing strain	Strain source	Architectural feature	Ref.
**440–446**	*Fusarium heterosporum* CNC-477	Driftwood collected from a mangrove habitat at Sweetings Cay, Bahamas	Sesterterpenes	[246]
**447–449**	*Aspergillus* CNK-371	Unidentified sponge collected at 40 feet from Manele Bay, Lanai, Hawaii	Meroterpenoids	[247]
**450–451**	*Phomopsis tersa* FS441	Deep-sea sediment sample	Meroterpenoids	[248]
**452**	*Aspergillus insuetus* (OY-207)	The Mediterranean sponge Psammocinia sp., Sdot-Yam, Israel	Meroterpenoid	[249]
**453–454**	*Aspergillus* sp. 094102	Zoanthid *Zoanthus* collected at Ayamaru Cape, Amami Island, Kagoshima Prefecture, Japan	Sesterterpenes	[250]
**455–456**	*Penicillium* sp. 303#	Sea water, Zhanjiang Mangrove National Nature Reserve in Guangdong Province, China	Meroterpenoids	[163]
**2.3.4. Sterols**				
**457**	*Gymnacella dankaliensis*	Sponge *Halichondria japonica*, Osaka Bay, Japan	Sterols	[251]
**458–461**	*Gymnascella dankaliensis*	Sponge *Halichondria japonica*, Osaka Bay, Japan	Sterols	[252]
**462–467**	*Rhizopus* sp.	Marine *Bugula* sp., Jiaozhou Bay, China	Ergosterols	[253]
**468**	*Penicillium chrysogenum* QEN-24S	Unidentified marine red algal species of the genus *Laurencia*	Sterols	[254]
**469–470**	*Aspergillus niger* MA-132	A fresh healthy sample of the mangrove plant *Avicennia marina*	Sterols	[255]
**471–473**	*Penicillium purpurogenum* G59	Soil sample collected at the tideland of Bohai Bay, China	Sterols	[256]
**474**	*Penicillium citrinum* SCSIO 41017	Sponge *Callyspongia* sp. collected from the sea area near Xuwen County, Guangdong Province, China	Sterol	[257]

Compound	Producing strain	Strain source	Architectural feature	Ref.
**475**	*Aspergillus flavus* YJ07-1	Bohai Sea	Oxygenated steroid	[258]
**2.4. Hybrids**				
**2.4.1. Hybrids of polyketides and peptides (or amino acids)**				
**476–477**	*Penzczllzum fellutanum* Btourge	Marine fish *Apogon endekataenia* Bleeker	Hybrids	[259]
**478–483**	*Aspergillus fumigatus*	Marine fish *Pseudolabrus japonicus*	Hybrids	[260]
**484–486**	*Gliocladium roseum* OUPS-N132	The sea hare *Aplysia kurodai* collected in coast of Kata	Hybrids	[192]
**487–488**	*Penicillium aurantiogriseum* SP0-19	Sponge *Mycale plumose* (Mycalidae), Jiaozhou Bay, Qingdao, China	Hybrids	[261]
**489–490**	*Acrostalagmus luteoalbus* SCSIO F457	Deep-sea sediment	Hybrids	[262]
**491–492**	*Penicillium purpurogenum* G59		Hybrids	[170]
**493–495**	*Leptosphaeria* sp. OUPS-4	Marine alga *Sargassum tortile*	Hybrids	[183]
**496**	*Trichoderma virens* CNL910	A sample of the marine ascidian *Didemnum mole*, Madang, Papua New Guinea	Hybrids	[263]
**497–498**	*Microsporum* cf. *gypseum* CNL-629	A sample of the bryozoan *Bugula* sp., U.S. Virgin Islands	Hybrids	[264]
**499–501**	*Penicillium* sp.	Marine alga *Enteromorpha intestinalis*, Tanabe Bay, Japan	Hybrids	[265]
**502–506**	*Penicillium* sp. OUPS-79	Marine alga Enteromorpha intestinalis	Hybrids	[266]
**507**	*Phomopsis asparagi*	Sponge *Rhaphidophlus juniperina*	Hybrids	[267]
**508–510**	*Spicaria elegans* KLA03	Marine sediments, Jiaozhou Bay, China	Hybrids	[268]
**511–512**	*Spicaria elegans* KLA03	Marine sediments, Jiaozhou Bay, China	Hybrids	[269]
**513**	*Spicaria elegans* KLA03	Marine sediments, Jiaozhou Bay, China	Hybrids	[270]

Compound	Producing strain	Strain source	Architectural feature	Ref.
**514–515**	*Spicaria elegans* KLA03	Marine sediments, Jiaozhou Bay, China	Hybrids	[271]
**516–517**	*Spicaria elegans* KLA03	Marine sediments, Jiaozhou Bay, China	Hybrids	[272]
**518**	*Xylaria* sp. SCSIO156	Marine sediment, South China Sea	Hybrids	[273]
**519–522**	*Phoma* sp.	Giant jellyfish *Nemopilema nomurai*	Hybrids	[274]
**523**	*Aspergillus oryzae*	Red Sea sediments collected off Jeddah, Saudi Arabia	Hybrids	[275]
**524**	*Aspergillus versicolor*	Marine sponge *Petrosia* sp.	Lipopeptide	[276]
**525**	*Aspergillus terreus* SCSGAF0162	The tissue of the gorgonian *Echinogorgia aurantiaca*, Sanya, Hainan Province, China	Cyclic tetrapeptide	[277]
**526–527**	*Aspergillus clavatus* C2WU	Hydrothermal vent crab *Xenograpsus testudinatus*, Kueishantao, Taiwan	Cyclodepsipeptides	[278]
**528–534**	*Penicillium purpurogenum* G59	Soil sample collected at the tideland of Bohai Bay, China	Lipopeptides	[279]
**535**	*Aspergillus*	Brown algal species belonging to the genus *Sargassum* collected off Helgoland, North Sea, Germany	Cyclotripeptide	[280]
**536**	*Aspergillus versicolor* SCSIO 41016	Marine sponge	Diketopiperazine alkaloids	[281]
**537**	*Aspergillus sydowii* MCCC 3A00324	Deep sea sediment (2246 meters), South Atlantic Ocean	Hybrids	[282]
**538**	*Penicillium citrinum*	Deep-sea sediment sample	Pentacyclic alkaloid	[283]
**539–540**	*Aspergillus* sp.	Marine-submerged decaying wood	Lipopeptidyl benzophenones	[284]
**2.4.2. Hybrids of polyketides and terpenoids (or steroids or isoprenyls)**				
**541**	*Aspergillus versicolor* CNC 327	Caribbean green alga *Penicillus capitatus*	Sesquiterpenoid nitrobenzoyl ester	[285]
**542**	*Gymnacella dankaliensis*	Sponge *Halichondria japonica*, Osaka Bay, Japan	Hybrid	[252]

Compound	Producing strain	Strain source	Architectural feature	Ref.
**543**	*Hypoxylon croceum* M97-25	Driftwood in a mangrove estuary in the Everglades/Florida	Sordarin derivative	[286]
**544**	*Xylariaceous* LL-07H239		Eremophilane sesquiterpene	[287]
**545**	*Chaetomium globosum*	Fresh algal sample, Qingdao coastline, *Polysiphonia urceolata*	Hybrids	[288]
**546–547**	*Aspergillus ustus* 8009	Marine sponge *Suberites domuncula*, Adriatic Sea	Drimane sesquiterpenoids	[289]
**548–549**	*Phoma* sp.	Marine sponge *Ectyplasia perox*, the Caribbean Sea, Dominica	Prenylated polyketides	[290]
**550**	*Paraconiothyrium* sp. 193H12	Marine sponge *Ectyplasia perox*, the Caribbean Sea, Dominica	Hybrid	[291]
**551**	*Aspergillus insuetus* (OY-207)	The Mediterranean sponge Psammocinia sp., Sdot-Yam, Israel	Drimane sesquiterpenoids	[249]
**552**	*Aspergillus ustus*	Rhizosphere soil of the mangrove plant *Acrostichum aureurm*, Guangxi Province, China	Drimane sesquiterpenoids	[292]
**553–554**	*Aspergillus ustus* 094102	Rhizosphere soil of the mangrove plant Bruguiera gymnorrhiza, Wenchang, Hainan Province of China	Drimane sesquiterpenoids	[293]
**555**	*Penicillium* sp. F00120	Sediment of the northern South China Sea	Sesquiterpene quinone	[294]
**556**	*Penicillium* sp. C9408-3	Deep sea sediment sample collected at a depth of 5115 m	Breviane spiroditerpenoid	[295]
**557**	*Neosartorya laciniosa* KUFC 7896	Coastal forest soil, Samaersarn island, Chonburi Province, Thailand	Hybrids	[296]
**558–560**	*Aspergillus versicolor* OUPS-N136	Sea urchin *Anthocidaris crassispana* collected in Tanabe Bay in Wakayama, Japan	Hybrids	[297]

Compound	Producing strain	Strain source	Architectural feature	Ref.
**561**	*Cryptosphaeria* sp. CNL-523	Unidentified ascidian collected in the Bahamas	Ester-substituted sesquiterpenoid	[298]
**562**	*Penicillium concentricum* ZLQ-69	Water sample taken from the coast of the Bohai Sea in Binzhou, Shandong Province, China	Meroterpene	[299]
**563–566**	*Aspergillus flavus* CF13-11	Marine sediment collected from the Bohai Sea	Drimane sesquiterpene esters	[300]
**567**	*Paecilomyces* sp.	Saprophytic bark of mangrove from the Taiwan Strait	Hybrids	[301]
**568–569**	*Penicillium expansum* 091006	Surface-sterilized roots of the mangrove plant *Excoecaria agallocha*, Wenchang, Hainan Province, China	Hybrids	[302]
**570–571**	*Penicillium expansum* 091006	Surface-sterilized roots of the mangrove plant *Excoecaria agallocha*, Wenchang, Hainan Province, China	Hybrids	[303]
**572**	*Aspergillus ustus* 094102	Rhizosphere soil of the mangrove plant Bruguiera gymnorrhiza, Wenchang, Hainan Province of China	Isochromane derivative	[293]
**573**	*Nigrospora* sp. MA75	Stem of the semi-mangrove plant *Pongamia pinnata*	Hybrids	[304]
**574–575**	*Stachylidium* sp. 220	Sponge *Callyspongia* sp. cf. *C. flammea*, Bear Island, Sydney, Australia	Phthalide derivatives	[305]
**576–577**	*Stachylidium* sp. 220	Sponge *Callyspongia* sp. cf. *C. flammea*, Bear Island, Sydney, Australia	Phthalimidine derivatives	[306]
**578–588**	*Alternaria* sp. JJY-32	Sponge *Callyspongia* sp. collected off the coast of Hainan Island, China	Hybrids	[307]
**589**	*Neosartorya laciniosa* KUFC 7896	Coastal forest soil, Samaersarn island, Chonburi Province, Thailand	Meroditerpene	[296]

Compound	Producing strain	Strain source	Architectural feature	Ref.
**590**	*Penicillium* sp. ZLN29	Sediments collected, Jiaozhou Bay, China	Penicillide derivative	[308]
**591**	*Penicillium Canescentia* MMS35	Seawater sample gathered on the French Atlantic coast near the Loire River estuary	Chlorinated sesquiterpenoid	[309]
**592**	*Penicillium* sp. FJ-1	Marine plant of *Avicennia marina*, Fujian, China	Hybrids	[220]
**593–594**	*Aspergillus terreus* OUCMDZ-1925	Viscera of *C. haematocheilus* grown in the waters of the Yellow River Delta	Hybrids	[310]
**595–596**	*Aspergillus flavus* OUCMDZ-2205	Prawn *Penaeus vannamei* from the Lianyungang Sea area, Jiangsu Province of China	Indole-diterpenoids	[311]
**597**	*Stachybotrys* sp. MF347	Driftwood sample collected at Helgoland, North Sea, Germany	Hybrids	[312]
**598–600**	*Mucor irregularis* QEN-189	Mangrove plant *Rhizophora stylosa*, Hainan Island, China	Indole-diterpenes	[313]
**2.4.3. Hybrids of peptides and terpenoids (or isoprenyls)**				
**601**	*Aspergillus ustus* NSC-F038		Hybrids	[314]
**602–604**	*Aspergillus* sp.	Mussel Mytilus edulis, Noto Peninsula, Sea of Japan	Indole alkaloids	[315]
**605**	*Aspergillus* sp.	Mussel Mytilus edulis, Noto Peninsula, Sea of Japan	Prenylated indole alkaloid	[316]
**606–609**	*Aspergillus fumigatus*	Holothurian S. japonicus, Lingshan Island, Qingdao, China	Prenylated indole diketopiperazine	[317]
**610–612**	*Aspergillus sydowi* PFW1-13	Driftwood sample beach of Baishamen, Hainan, China	Diketopiperazine alkaloids	[318]
**613**	*Aspergillus sclerotiorum* PT06-1	Sediments collected in the Putian salt field, Fujian Province of China	Hybrids	[319]

Compound	Producing strain	Strain source	Architectural feature	Ref.
**614–615**	*Aspergillus fumigatus* YK-7	Sea mud of intertidal zone collected from Yingkou, China	Hybrids	[320]
**616–617**	*Aspergillus westerdijkiae* DFFSCS013	Marine sediment sample collected in the South China Sea	Prenylated indole alkaloids	[321]
**618**	*Penicillium crustosum* HDN153086	Antarctic sediment, Prydz Bay	Hybrids	[322]
**2.4.4. Other hybrids**				
**619**	*Penicillium citrinum*	Red alga *Actinotrichia fragilis,* Hedo Cape, Okinawa Island	Hybrids	[323,324]
**620**	*Gliocladium* sp. YUP08	Sea mud collected in Rushan, Shandong province, China	Hybrids	[325,326]
**621–622**	*Gliocladium* sp. YUP08	Sea mud collected in Rushan, Shandong province, China	Piperazine-2,5-dione derivatives	[326]
**623**	*Aspergillus effuses* H1-1	The mud under mangroves along the coast of Fujian province, China	Spiro-polyketide-diketopiperazine hybrid	[327]
**624–625**	*Neosartorya fischeri*	Marine mud in the intertidal zone of Hainan Province of China	Hybrids	[328]
**626**	*Aspergillus versicolor* HDN08-60	Sediments collected in the South China Sea	Hybrids	[329]
**2.5. Others**				
**627–628**	*Penicillium* sp.	Marine alga *Enteromorpha Intestinalis*	Others	[330]
**629–630**	*Penicillium* sp.	Sponge *Axinella verrucose,* Mediterranean Sea	Others	[331]
**631**	*Penicillium* sp.	Mangrove	Pyrrolyl 4-quinolinone alkaloid	[332]
**632**	*Fusarium incarnatum* (HKI0504)	Mangrove plant *Aegiceras corniculatum*	Others	[125]
**633**	*Acremonium strictum*	Unidentified Choristida sponge collected from Korean waters	Others	[333]
**634**	*Penicillium aurantiogriseum*	Marine mud of the Bohai Sea	Others	[334]

Compound	Producing strain	Strain source	Architectural feature	Ref.
**635**	*Penicillium* sp. F23−2	Deep sea	Others	[159]
**636–637**	*Penicillium paneum* SD-44	Sediment sample collected from the South China Sea	Others	[335]
**638–640**	*Aspergillus violaceus* WZXY-m64-17	Sponge	Others	[336]
**641**	*Aspergillus terreus* [CFCC 81836]		Others	[337]
**642**	*Aspergillus niger* BRF-074	Sediments of the Northeastern coast of Brazil	Others	[338]
